# Do the basic crystal chemistry principles agree with a plethora of recent quantum chemistry data?

**DOI:** 10.1107/S2052252518008254

**Published:** 2018-07-20

**Authors:** Elena Levi, Doron Aurbach, Carlo Gatti

**Affiliations:** a Department of Chemistry, Bar-Ilan University, Ramat-Gan 5290002, Israel; b CNR-ISTM Istituto di Scienze e Tecnologie Molecolari, via Golgi 19, Milano I-20133, Italy; c Istituto Lombardo Accademia di Scienze e Lettere, via Brera 28, Milano I-20121, Italy

**Keywords:** chemical bonding, bond order, bond lengths, bond critical point, electron density, conservation principles

## Abstract

The main descriptors of chemical bonding such as bond order (BO) and electron density at the bond critical point, ρ_c_, are customarily used to understand the crystal and electronic structure of materials, as well as to predict their reactivity and stability. They can be obtained in the framework of crystal chemistry and quantum chemistry approaches, which are mostly applied as alternatives to each other. This paper verifies the convergence of the two approaches by analyzing a plethora of quantum chemistry data available in the literature. The exponential correlation between the electron descriptors [BO*_ij_* and ρ_c(*ij*)_] and the length of chemical bonds, *R_ij_*, which is basic in crystal chemistry, was confirmed for 72 atom pairs, regardless of the nature of their interactions (ionic/covalent, metal–metal, *etc*.). The difference between the BO*_ij_* (*R_ij_*) correlations obtained in this work and those accepted in crystal chemistry for the same atomic pairs does not exceed the dispersion of quantum chemistry data, confirming the qualitative validity of the BO conservation principle. Various examples are presented to show that knowledge of the exponential parameters ensures a surprisingly simple determination of two basic electron descriptors in any complex compound with known interatomic distances. In particular, the BO analysis for 20 Re_6_-cluster complexes illustrates the BO conservation for systems with delocalized electrons. Despite the significant transfer of electron density from the Re–Re to the Re–ligand bonds, the total number of Re valence electrons used in bonding remains close to the formal value of seven electrons.

## Abstract 2

Electron descriptors such as bond order and electron density at the bond critical point are key quantities in establishing the characterization of chemical bonding. This paper analyzes a plethora of quantum chemistry data available in the literature to confirm exponential correlations between these electron descriptors and the interatomic distances for any type of chemical bond. This is particularly relevant for extending the crystal chemistry bond order conservation principle to systems with delocalized electrons.

## 1. Introduction   

Chemical bonding is a fundamental concept in materials science. The interaction between two atoms can be quantitatively described by several bond descriptors. Those of particular relevance, because of their direct physical meaning, simply or universally, are bond order BO*_ij_*, which is the number of electron pairs shared between atoms *i* and *j* (Pauling, 1960 ▸), and electron density (ED) at the bond critical point (ρ_c_), which should be related to each other (Bader, 1994 ▸). These descriptors are widely used to understand the crystal and electronic structure of materials (Koritsanszky & Coppens, 2001 ▸; Gatti, 2005 ▸; Gatti & Macchi, 2012 ▸), and to predict their reactivity and stability (a classic example of the direct correlation between bond order and the average bond energy for the C—C, C—O and C—N pairs is presented in Fig. S1 in the supporting information). For a given *ij* atom pair, these electron descriptors are closely related to the bond length, *R_ij_*, but an exact formulation of this relationship is lacking and the proposed correlations essentially depend on the sampled distances and atomic pairs. For example, Bader *et al.* (1982 ▸) suggested the linear ρ_c(*ij*)_ (*R_ij_*) correlation, however, a variety of studies (see, for instance, Espinosa *et al.*, 2002 ▸; Dominiak *et al.*, 2006 ▸; Gibbs *et al.*, 2014 ▸) showed the power or (single or double) exponential ρ_c(*ij*)_ (*R_ij_*) distribution. The question about the BO*_ij_*(*R_ij_*) relationship is even more complicated. The empirical Pauling approach (Pauling, 1960 ▸; Brown, 2009 ▸) to the determination of BO*_ij_* suggests the exponential fitting: 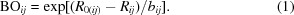



Here *R*_0(*ij*)_ and *b_ij_* are the bond valence parameters, which are transferable for a given atom pair in different compounds. *R*_0(*ij*)_ describes the effective repulsion between *i* and *j* atoms, whereas *b_ij_* is often related to the softness of the bond (Chen & Adams, 2017 ▸). Equation (1) is widely accepted in crystal chemistry, where it is used to check the reliability of structure solutions, estimate cation oxidation states or possible lattice strains, analyze ionic motion or surface phenomena *etc.* (Shustorovich, 1990 ▸; Brown, 2009 ▸; Levi & Aurbach, 2014 ▸; Adams & Rao, 2014 ▸). Although there have been a number of attempts to theoretically justify equation (1), and to calculate the *R*_0(*ij*)_ and *b_ij_* from quantum chemistry considerations (Mohri, 2003 ▸, 2005 ▸; Hardcastle & Laffoon, 2012 ▸; Adams, 2013 ▸), this approach remains empirical and is regarded by a large part of the scientific community as old-fashioned and simplistic. Moreover, correlation (1) was claimed inapplicable to compounds with delocalized electrons, particularly for metal–metal bonds (Cotton *et al.*, 2005 ▸; Brown, 2009 ▸).

The fitting of the *R*_0(*ij*)_ and *b_ij_* values is commonly performed to satisfy the rule of local electroneutrality, which states that the bond valence sum, BVS = ΣBO*_ij_*, around atom *i* should be equal to its valence, *V_i_*

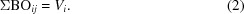



This means that the fitting of the bond valence parameters in crystal chemistry is based on the formal values of shared electrons. In contrast, various calculation methods based on quantum mechanical properties [Wiberg BO, Wiberg (1968 ▸); Mayer BO, Mayer (1983 ▸); natural BO, Glendening & Weinhold (1998 ▸); fuzzy BO, Mayer & Salvador (2004 ▸); delocalization index, Bader & Stephens (1975 ▸); Fradera *et al.* (1999 ▸); Matito *et al.* (2005 ▸) *etc*.] are related to the effective number of shared electron pairs, which might differ significantly from the formal values, especially in the case of metal–metal bonds (Roos *et al.*, 2007 ▸). For example, the effective BO for the quadruple Re—Re bond (formal BO = 4) in K_2_Re_2_Cl_8_·H_2_O is equal to 3.2 v.u. (Ponec *et al.*, 2010 ▸). In this case, equation (2) is irrelevant and only the data of quantum chemistry calculations should be used to establish the character of BO*_ij_*–*R_ij_* correlation (Levi & Aurbach, 2011 ▸; Levi *et al.*, 2013*a* ▸,*b*, 2014 ▸; Singh *et al.*, 2016 ▸). Unfortunately, the data obtained for the same compounds by different quantum chemistry methods give high BO dispersions and, in many cases, show the absence of a clear BO*_ij_*–*R_ij_* relationship. This is not surprising, since the BO values are known to be largely affected by the more or less accurate treatment of electron correlation and by the more or less exact form adopted for the pair density and/or the exchange-correlation density.

Despite the high dispersion, it is logical to suggest that analysis of an extensive set of quantum chemistry data will clarify the issue by showing the presence or absence of qualitatively acceptable correlations between the electron descriptors, ρ_c(*ij*)_ and BO*_ij_*, and the length of chemical bonds, *R_ij_*. The result is of some interest for our understanding of the universal features of chemical bonding. Moreover, if such correlations exist, knowledge of their parameters will allow for a surprisingly simple calculation of two basic electron descriptors for any atomic arrangement. Thus, this paper analyzes the numerous quantum chemistry data available in the literature in order to verify not only the empirical validity of equation (1) for different types of chemical bonds, but also whether the ED at the bond critical points, ρ_c(*ij*)_, similarly decays with increasing bond length, *R_ij_*. By analogy with the Pauling equation, we will express this decay in the following form: 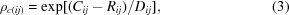
where *C_ij_* and *D_ij_* are constants for a given atom pair in different compounds.

Our aim is also to empirically quantify the relationships between the two basic descriptors ρ_c(*ij*)_ and BO*_ij_*, which should be in some way related to each other. To date, different types of the BO*_ij_*–ρ_c(*ij*)_ correlation have been proposed (Bader *et al.*, 1983 ▸; Howard & Lamarche, 2003 ▸; Tsirelson *et al.*, 2007 ▸).

It is worth emphasizing that in this study we are interested in the uniform character of chemical bonds evident from Pauling’s principles, while differences in the bond nature such as closed-shell or open-shell, weak or strong interactions, will be rather out of the scope of this paper. These differences can be described further and in more detail by additional ED topological parameters such as Laplacians of the ED and potential and kinetic energy densities at the bond critical point [see, for instance, Espinosa *et al.* (1998 ▸, 2002 ▸) and Mata *et al.* (2010 ▸)].

## 2. Methods   

The determination of the bond order parameters, *R*_0(*ij*)_ and *b_ij_*, as well as the *C_ij_* and *D_ij_* constants (listed in Table S1 of the supporting information) was based on the exponential fitting of the BO*_ij_*– and the ρ_c(*ij*)_–distance curves presented in Figs. S2 and S3. All of the data [BO*_ij_*, ρ_c(*ij*)_ and *R_ij_*] for these curves were taken from the original quantum chemistry calculations found in more than 1000 references. Note that no special criterion was used to include quantum chemistry data in the BO*_ij_* and ρ_c(*ij*)_ analysis. Figs. S2 and S3 show that possible ‘bad’ quantum chemistry data result in higher BO*_ij_* and ρ_c(*ij*)_ dispersion, but do not change the exponential character of established correlations. The constants, *R*_0(*ij*)_, *b_ij_*, *C_ij_* and *D_ij_*, obtained from the exponential fitting, were used to calculate BO*_ij_* and ρ_c(*ij*)_ using equations (1) and (3), respectively. To confirm the validity of the exponential correlations, the BO*_ij_* and ρ_c(*ij*)_ values calculated were compared with the original quantum chemistry data. It should be noted that the estimates for ρ_c(*ij*)_ values using equation (3) assume the presence of a bond path, hence, a bond critical point (BCP), between the associated atom pairs. Whether such a path is present or not can in general only be determined through the ∇ρ vector field. In the case of the data used below, bond path and BCP occurrence are ensured by the original works (see the references in the supporting information).

## 3. Results and discussion   

### 3.1. Analysis of quantum chemistry data   

For our analysis we chose 72 atom pairs with completely different types of interactions, from ionic/covalent, hydrogen to metal–metal bonds (Table S1). The ρ_c(*ij*)_ and BO*_ij_* values, available in the literature for a large spectrum of compounds, from simple binaries to complex organic and metal–organic complexes (see the references in the supporting information), were presented as a function of the bond length, *R_ij_*, separately for each pair (see examples in Figs. S2 and S3, respectively). An important requirement for an unbiased analysis was a wide *R_ij_* range, because the wider the *R_ij_* range, the more general the established correlation is. Indeed, in the narrow *R_ij_* range, an exponential relationship can be easily confused with a linear one. As can be seen from Table S1, for all pairs, the quantum chemistry data agree reasonably well with exponential correlations (1) and (3), although in a few cases, the number of available data was insufficient to properly establish the character of this correlation. Based on the exponential fitting of the BO*_ij_*– and the ρ_c(*ij*)_–distance curves, the BO (or bond valence) parameters, *R*_0(*ij*)_ and *b_ij_*, as well as the *C_ij_* and *D_ij_* constants were determined for most of the pairs (Table S1).

For comparison, Fig. S3 also presents the BO curves (marked in red) used in crystal chemistry. Since there are a number of different bond valence parameters [*R*_0(*ij*)_ and *b_ij_*] proposed in the literature for the same atom pairs (Brown, 2016 ▸), we chose those closest to the parameters obtained in this work using quantum calculation data. As can be seen for many atom pairs, crystal chemistry and quantum chemistry curves are very similar, and their differences do not exceed the dispersion of quantum chemistry data. The curve distinctions may be caused by different parameters of exponential decay (*b_ij_*) which are difficult to determine properly by crystal chemistry methods (in most cases, *b_ij_* is accepted as constant and equal to 0.37 Å). Despite this *b_ij_* uncertainty, the similarity of the curves shows that equation (2) is approximately valid for most of the atom pairs, *i.e.* the effective number of shared electron pairs is very close to the formal one. As demonstrated below, the exception is the metal–metal bonds with a more complicated rule of local electroneutrality (Levi & Aurbach, 2011 ▸; Levi *et al.*, 2013*a* ▸,*b*, 2014 ▸; Singh *et al.*, 2016 ▸).

**Figure 1:**
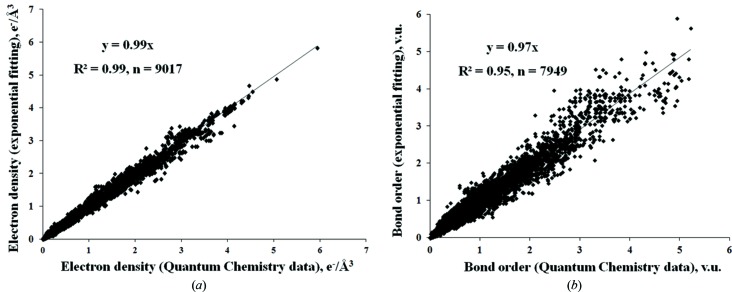
Crystal chemistry *versus* quantum chemistry results. The electron descriptors (*a*) ρ_c(*ij*)_ and (*b*) BO*_ij_*, were obtained in this work by exponential fitting of equations (3) and (1), respectively, against the same descriptors calculated by quantum chemistry methods (literature data). *n* is the number of data points.

Interestingly, there is a clear correlation (and even relatively close absolute values) between the BO parameter *R*_0(*ij*)_ and the ED constant *C_ij_* (Fig. S4). As expected from the structure of equations (1) and (3), both increase with the cumulative size of the *i* and *j* atoms (Fig. S5). In contrast, we did not see any visible correlation between *b_ij_* and *D_ij_*, but the absence of such correlation may be caused by the relatively high dispersion of the BO*_ij_* data. Using the parameters listed in Table S1, we calculated ρ_c(*ij*)_ using equation (3) and BO*_ij_* using equation (1), and then compared them with the literature data (Fig. 1 ▸). Linear relationships between calculated values and the initial quantum chemistry data confirm the existence of the exponential correlation: *R* ^2^ is equal to 95% for BO*_ij_* calculated using equation (1) and 99% for ρ_c(*ij*)_ calculated using equation (3). It is evident from Fig. 1 ▸ that the original ρ_c(*ij*)_ values are almost quantitatively reproduced by the correlations obtained, whereas the agreement of the original BO*_ij_* with their calculated values is at best qualitative.

The existence of such correlations is not surprising, given the exponential decay of atomic and molecular wavefunctions (or orbitals), and may be qualitatively justified on the basis of very simple reasoning. Model expressions for the ED between two atoms show that the ED, at any point along the internuclear axis (hence also at the BCP), depends on quantities that all decay exponentially with bond distance [see equations (2 *a*) and (2*c*) in Gatti & Fantucci (1993 ▸)], including the overlap integral between basis functions centered on the nuclei of the two atoms [see equation A.9 in Szabo & Ostlund (1982 ▸)]. Delocalization indices, and therefore the BO*_ij_*, are also given in terms of the products of molecular or natural orbital integral overlaps, yet defined over the Bader’s domains of each of the two bonded atoms [*e.g.* Poater *et al.* (2005 ▸) and equations 1.48 and 1.49 in Gatti & Macchi (2012 ▸)]. However, one may surmise that, in such a case, the overlap integrals between different orbitals might decay exponentially with the internuclear distance for a pair of orbitals centered on the nuclei of two interacting atoms, while the orbital self-overlaps should remain almost constant *versus R_ij_*, and not significantly affect the BO*_ij_*.

A combination of equations (1) and (3) results in the power correlation between BO*_ij_* and ρ_c(*ij*)_ for a given atom pair: 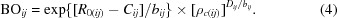



The relationships between ρ_c(*ij*)_ and BO*_ij_* for a number of atom pairs are presented in Fig. S6, which shows they are quasi-linear for most of the pairs, while the slope of the lines seems to agree with the type of interactions. For example, the low values of the ED at ρ_c(*ij*)_ for the metal–metal pairs can be explained by the diffuse nature of TM–TM bonds (TM = transition metal) (Farrugia & Macchi, 2010 ▸). Thus, in spite of the quasi-linear correlation and the similar dependence of the interatomic distances, the ED at ρ_c(*ij*)_ and BO*_ij_* represent different bonding features and cannot always replace each other in descriptions of atom interactions.

Interestingly, other parameters of the ED topology, which are not related to Pauling’s principles, may also be presented as functions of the interatomic distances or BO (see Fig. S7 and the short comments in the supporting information). As was mentioned in the *Introduction* [Sec sec1], these parameters are used to describe the differences in the nature of the bonding interactions, as it evolves with the bonding distance.

In spite of the apparent triviality and simplicity of our results, it is hard to overestimate the importance of equations (1) and (3) for materials science. They allow for the following.

(i) Easy access to a qualitative estimate of two basic electron descriptors for any kind of atomic interactions in any complex compound with known interatomic distances (see below and the examples in the supporting information). The calculations can be performed in the program *Excel*, and they take only a few minutes instead of several days.

(ii) An empirical proof of the existence of universal qualitative exponential correlations between the bond descriptors BO*_ij_* or ρ_c(*ij*)_ and the length of a chemical bond, *R_ij_*.

(iii) A general correlation between two basic electron descriptors [equation (4)].

(iv) Possible rationalization of the exponential correlations between interatomic distances and some other physical descriptors of chemical bonds, *e.g.* stretching frequencies and force constants (Harvey, 1996 ▸; Da Re *et al.*, 2010 ▸; Kraka *et al.*, 2010 ▸). Indeed, although the BOs cannot be determined experimentally, it was shown that they are directly related to the vibrational properties of chemical bonds, and, respectively, to the force constants (Cremer & Kraka, 2010 ▸; Hardcastle & Wachs, 1990 ▸, 1991 ▸). To exemplify the linear correlation between the BOs and the square of the stretching frequencies, we used the interatomic distances and Raman spectroscopy data for the Mo–O bonds in various molybdates (Fig. S8) (Hardcastle & Wachs, 1990 ▸).

We can also expect reasonable accuracy of the results obtained using equations (1) and (3). To illustrate this, the first example in the supporting information draws a comparison between ρ_c(*ij*)_ obtained from equation (3) and by two calculation modes used in the original quantum chemistry work (Pyziak *et al.*, 2015 ▸): multipolar model I (more precise and close to the experimental data) and a standard independent atom model II. According to this comparison, the relative difference between ρ_c_ obtained from equation (3) and from model I for a given bond is, with exception for very weak intermolecular interactions (O⋯H, H⋯H, O⋯O), effectively smaller than the ρ_c_-difference between the two models for the same bond. All other examples in the supporting information demonstrate strong agreement between the results of the exponential fitting and the quantum chemistry data for different organic and metal–organic complexes. An additional criterion of the chemical validity of the BO analysis is a correct balance in the electron book-keeping. The last example in the supporting information presents such book-keeping for simple U_3_O_3_ and U_4_O_4_ clusters. The BO sum obtained from equation (1) was compared with the sum of the Wiberg bond indices in the original work (Tsipis *et al.*, 2008 ▸). As demonstrated, the BO sum for the U atoms (U—U and U—O bonds) is closer to the expected value of eight valence electrons than the sum of the Wiberg indices.

In addition, the next section[Sec sec3.2] presents an example of the BO analysis for a series of organometallic complexes based on Re_6_-clusters. We chose these complexes to illustrate the general principles of crystal chemistry [exponential valence-length correlation and bond-order conservation expressed by equations (1) and (2), respectively], because for years it was commonly accepted that these principles were not valid for a metal–metal bond (Cotton *et al.*, 2005 ▸). Moreover, in contrast to dinuclear Re_2_-cluster complexes, the quantum chemistry analysis of the effective BOs for the Re_6_-cluster compounds has, to the best of our knowledge, never been performed.

### 3.2. The BO analysis for a series of the Re_6_-cluster complexes   

**Figure 2:**
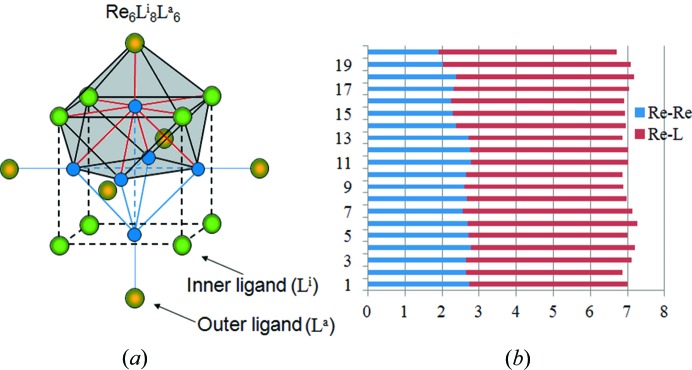
Structural and bonding features of the Re_6_-cluster compounds. (*a*) Octahedral Re_6_-cluster surrounded by eight inner and six outer ligands. The coordination polyhedron around an individual Re atom is marked in grey. (*b*) Validity of the conservation principle for the Re atoms in the Re_6_-cluster complexes. The numbers in the vertical scale correspond to the respective compounds in Table S2 in the supporting information, while the abscissa values correspond to the BO sum for the various compounds.

Metal–metal bonds in (TM)_*n*_-clusters are a widespread feature of inorganic and organometallic complexes. These bonds are commonly formed between TM atoms in low oxidation states when the TM valence electrons, unused in metal–ligand bonding, become available for additional metal–metal interactions. For example, in the Re_6_-cluster compounds, Re_6_ *L* ^i^_8_ *L* ^a^_6_ (*L* ^i^ = inner or bridging ligand, *L* ^a^ = outer, apical or terminal ligand) (Fig. 2 ▸ *a*), three valence electrons of the Re atom are used to form five metal–ligand bonds, while the remaining four electrons participate in four TM–TM interactions (formal BO_Re—Re_ = 1).

Since the TM atom in a cluster compound is bonded not only to the ligands but also to adjacent TM atoms (Fig. 2 ▸ *a*), the conservation principle expressed by equation (2) transforms into the following, 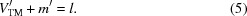



Here *V*′_TM_ and *m′* are the effective valences used by the TM to form TM–L and TM–TM bonds, respectively, while *l* is the total number of the TM electrons participating in all of the bonds.

If we know the interatomic distances, *R*_TM–L_ and *R*_TM–TM_, as well as the bond valence parameters for the TM–*L* and TM–TM pairs, the validity of equation (5) can be easily verified by using the valence–length correlations for all bonds of a given TM atom: 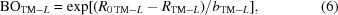


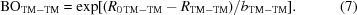



The BOs calculated by equations (6) and (7) can be used to calculate the number of valence electrons (effective valences) that participate in the TM–*L* and TM–TM bonding: 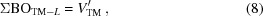


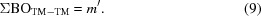



The results of the BO calculations for 20 Re_6_-cluster complexes are presented in Fig. 2 ▸(*b*) (the details can be found in Table S2 and the bond valence parameters used in these calculations are listed in Table S3). As shown, *V*′_TM_ and *m′* differ considerably from the formal values, *V*_TM_ and *m* (by at least one electron). However, for the Re_6_-cluster complexes, in accordance with equation (5), the deficiency of the ED for the TM–TM bonds, *m′* − *m*, is compensated for by their excess from the TM–*L* bonds, *V*′_TM_ − *V*_TM_, to preserve the local electroneutrality of the TM atom (*l* = 7 for Re): 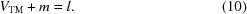



It was shown that the striking difference between formal and effective BOs of the TM–TM and TM–*L* bonds in cluster compounds is caused by a steric conflict between unusually short metal–metal bonds and a rigid ligand environment (Levi *et al.*, 2013*b* ▸; Singh *et al.*, 2016 ▸). The conflict results in lattice strain, while the redistribution of the ED between the TM–TM and TM–*L* bonds ensures their partial or full relaxation. Thus, the BO analysis of cluster compounds is especially interesting because it allows a correlation between structural features and deformability of the TM clouds.

## 4. Summary   

Systematic analysis of recent quantum chemistry data performed in this work confirms, at a qualitative level, the exponential character of the correlations between electron descriptors, such as BO*_ij_* or ρ_c(*ij*)_, and the length of chemical bonds, *R_ij_*. This character is universal because it is valid for any type of interaction (ionic, covalent *etc.*), including metal–metal bonds. It was shown that the BO*_ij_*–*R_ij_* correlations, based on the quantum chemistry data for a given *i–j* atom pair, are very close to those known in crystal chemistry in the framework of the bond valence model (BVM). The difference between them does not exceed the unavoidable dispersion of the quantum chemistry data, arising from the different degree of approximation of the wavefunctions from which they are derived. This result qualitatively confirms the BO conservation principle, which is the basis of the BVM. It also means that proper parameters of exponential correlations can be estimated based on a combination of structural and quantum chemistry data.

Thus, the work validates the BVM application to any type of compound, and we hope it will encourage materials scientists to apply the model to compounds with metal–metal bonds. The BVM is based on tabulated bond valence parameters. Our study shows that a similar database of empiric topological parameters, which will relate the ρ_c(*ij*)_ (and possibly other topological functions) to the interatomic distances, can be created based on a systematic analysis of the literature data. Such a database should allow for prediction of the ED topology in any complex compound.

## Supplementary Material

Additional data on the correlations between electron descriptors and interatomic distances for different atomic pairs, examples of the electron descriptor calculations and references for the quantum chemistry data. DOI: 10.1107/S2052252518008254/lc5099sup1.pdf
