# Supplementary material for: Do the basic crystal chemistry principles agree with a plethora of recent quantum chemistry data?
Source: IUCrJ. 2018 Jul 20;5(Pt 5):542–7. doi: 10.1107/S2052252518008254 (PMC6126658; doi:10.1107/S2052252518008254)
Supplement: Supplementary file 1 [file m-05-00542-sup1.pdf]

IUCrJ

Volume 5 (2018)

Supporting information for article:

Do the basic crystal chemistry principles agree with a plethora of recent quantum chemistry data?

Elena Levi, Doron Aurbach and Carlo Gatti

Supporting Information

Do the Basic Crystal Chemistry Principles Agree with a Plethora of Recent Quantum Chemistry Data?

Elena Levi*, Doron Aurbach and Carlo Gatti*

Table of Contents

1. Figure S1. Direct correlation between bond order and the average bond energy for three important atom pairs.......... 2

2. Figure S2. Exponential correlations between electron density, $\rho_{c\ ij}$, and the interatomic distances. The points are quantum chemistry data available in literature.......... 3-6

3. Figure S3. Exponential correlations between effective bond order, BO_{ij}, and the interatomic distances. The points are the quantum chemistry data available in literature. The crystal chemistry curves marked in red are presented for comparison.......... 7-10

4. Figure S4. Correlation between the *BO* parameter $R_{0\ ij}$ and electron density constant *Cij* .. 11

5. Figure S5. Bond order parameter $R_{0\ ij}$ (a) and electron density constant *Cij* (b) *vs*. the sum of the covalent radii of *i* and j atoms 11

6. Figure S6. Relationship between electron density and bond order for some atom pairs. 12

7. Figure S7. Additional parameters of the ED topology, which are not related to the Pauling's principles. A - Laplacian of the electron density as a function of the interatomic distance for the C-C, Fe-C, Cr-Cr and Fe-O bonds. B - Energy densities of the Cr-Cr bonds as a function of their *BOs*. 12

8. Figure S8. Linear correlation between the *BOs* of the Mo-O bonds calculated by Eq. 1 and the square of the stretching frequencies of various molybdates obtained in Raman spectroscopy… 13

9. Table S1. The results of the exponential fitting of the quantum chemistry data by Eqs 1 and 3: Bond valence parameters and electron density constants for typical atomic pairs. *n* is a number of data points.......... 14-17

10. Table S2. The results of the *BO* calculations for the Re_6-cluster complexes 18

11. Table S3. Bond valence parameters used in the *BO* calculations for the Re_6-cluster complexes… 19

12. Electron descriptors calculated by Eqs 1 and 3 (Examples) 20-25

13. References 26-42

Figure S1. Direct correlation between bond order and the average bond energy for three important atom pairs (The data from ref.[1]).

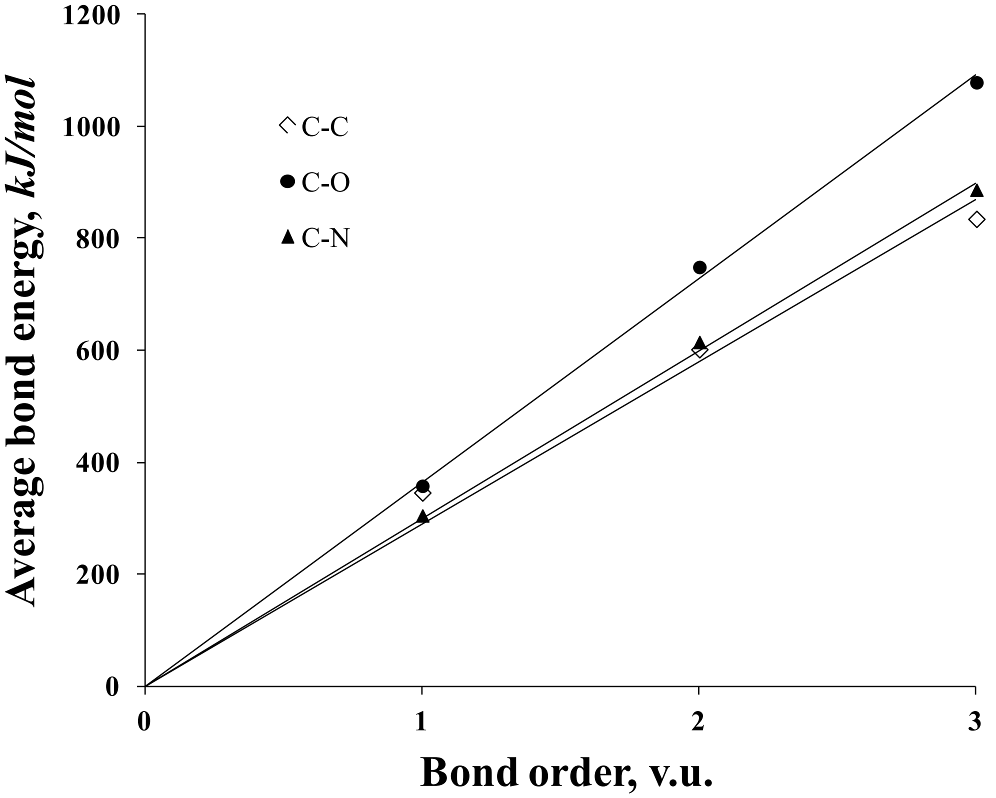

Figure S2. Exponential correlations between electron density, $\rho_{c\ ij}$, and the interatomic distances. The points are quantum chemistry data available in literature.

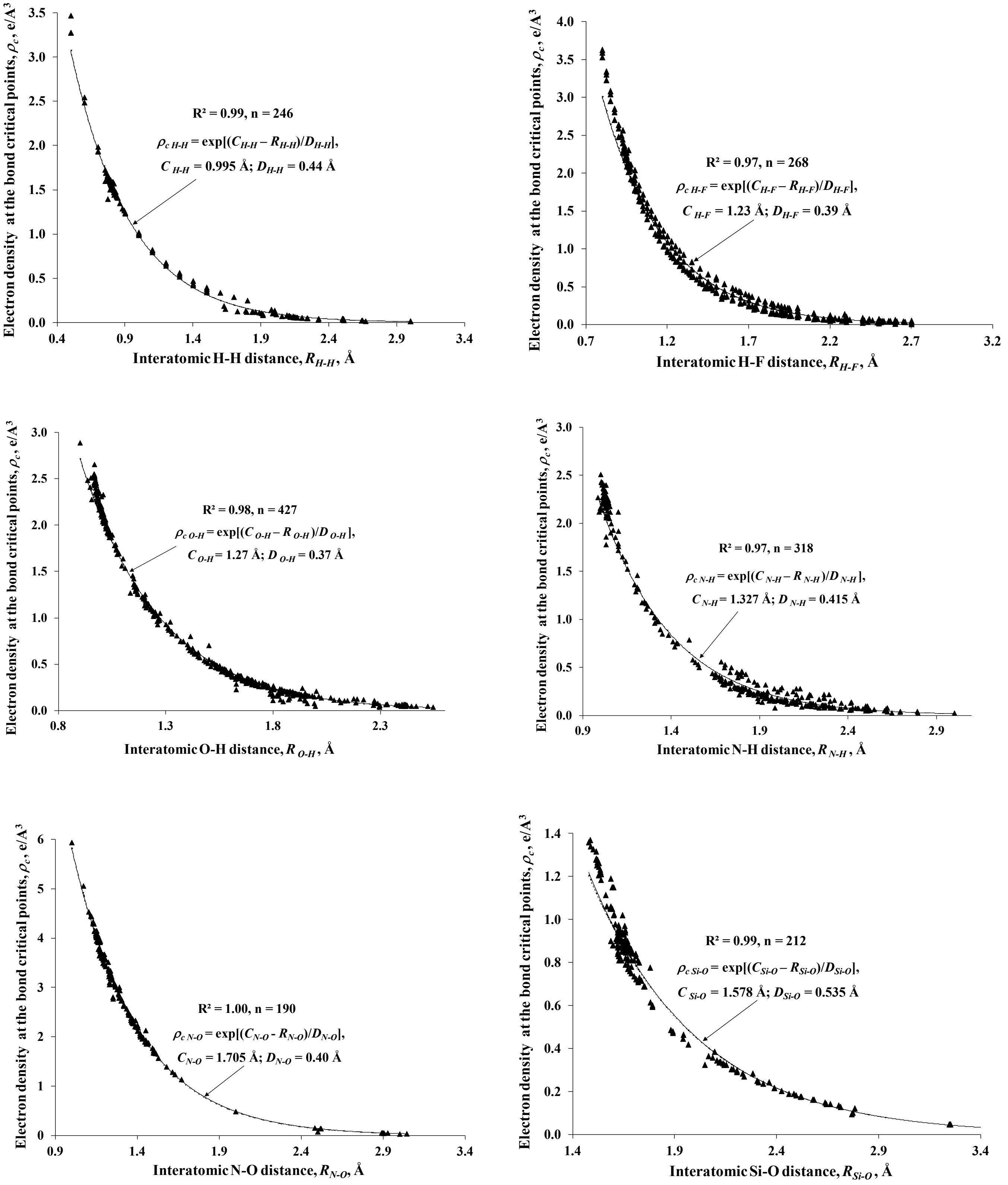

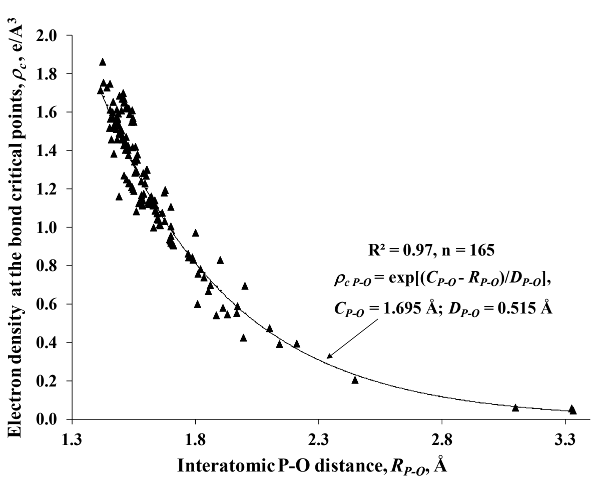

Electron density at the bond critical points, ρ_c, e/A^3
R² = 0.97, n = 165
$\rho_{c\,P\text{-}O} = \exp[(C_{P\text{-}O} - R_{P\text{-}O})/D_{P\text{-}O}]$,
$C_{P\text{-}O}$ = 1.695 Å; $D_{P\text{-}O}$ = 0.515 Å
Interatomic P-O distance, $R_{P\text{-}O}$, Å

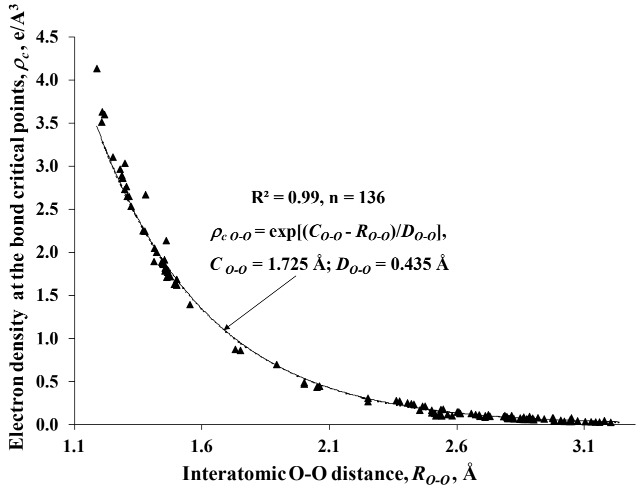

Electron density at the bond critical points, ρ_c, e/A^3
R² = 0.99, n = 136
$\rho_{c\,O\text{-}O} = \exp[(C_{O\text{-}O} - R_{O\text{-}O})/D_{O\text{-}O}]$,
$C_{O\text{-}O}$ = 1.725 Å; $D_{O\text{-}O}$ = 0.435 Å
Interatomic O-O distance, $R_{O\text{-}O}$, Å

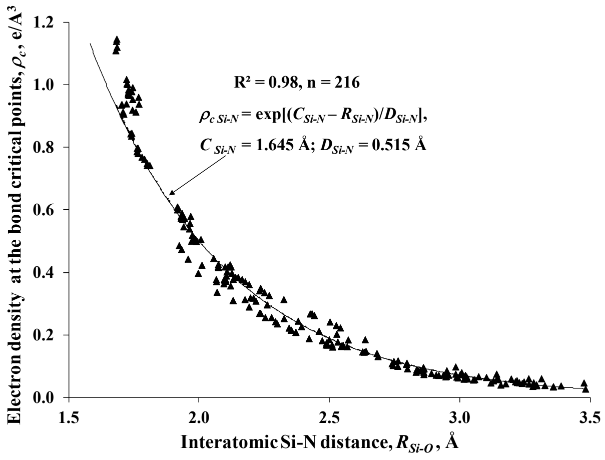

Electron density at the bond critical points, ρ_c, e/A^3
R² = 0.98, n = 216
$\rho_{c\,Si\text{-}N} = \exp[(C_{Si\text{-}N} - R_{Si\text{-}N})/D_{Si\text{-}N}]$,
$C_{Si\text{-}N}$ = 1.645 Å; $D_{Si\text{-}N}$ = 0.515 Å
Interatomic Si-N distance, $R_{Si\text{-}O}$, Å

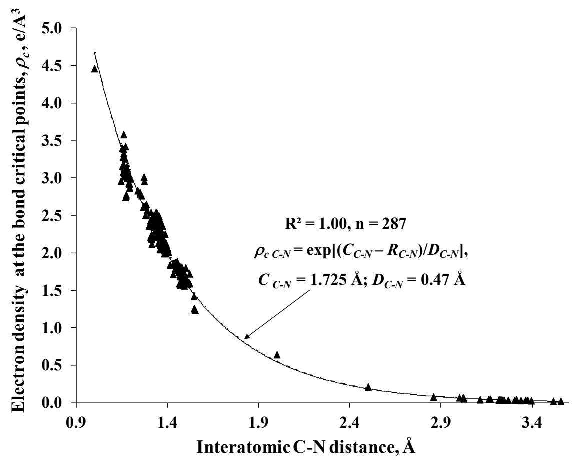

Electron density at the bond critical points, ρ_c, e/A^3
R² = 1.00, n = 287
$\rho_{c\,C\text{-}N} = \exp[(C_{C\text{-}N} - R_{C\text{-}N})/D_{C\text{-}N}]$,
$C_{C\text{-}N}$ = 1.725 Å; $D_{C\text{-}N}$ = 0.47 Å
Interatomic C-N distance, Å

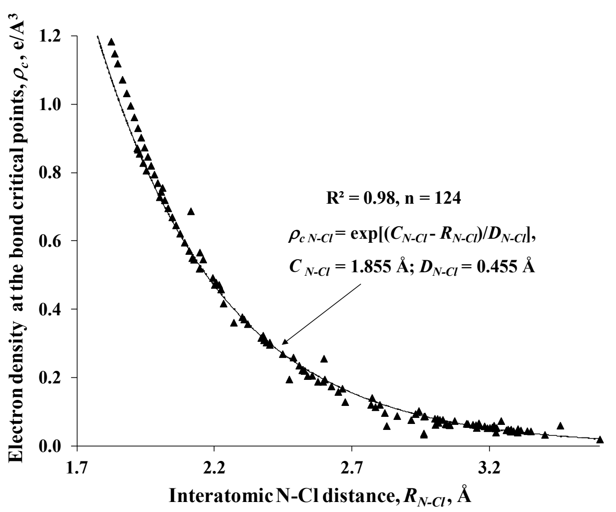

Electron density at the bond critical points, ρ_c, e/A^3
R² = 0.98, n = 124
$\rho_{c\,N\text{-}Cl} = \exp[(C_{N\text{-}Cl} - R_{N\text{-}Cl})/D_{N\text{-}Cl}]$,
$C_{N\text{-}Cl}$ = 1.855 Å; $D_{N\text{-}Cl}$ = 0.455 Å
Interatomic N-Cl distance, $R_{N\text{-}Cl}$, Å

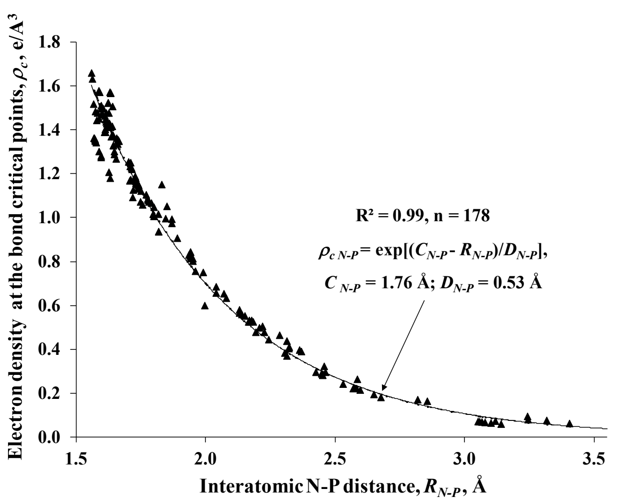

Electron density at the bond critical points, ρ_c, e/A^3
R² = 0.99, n = 178
$\rho_{c\,N\text{-}P} = \exp[(C_{N\text{-}P} - R_{N\text{-}P})/D_{N\text{-}P}]$,
$C_{N\text{-}P}$ = 1.76 Å; $D_{N\text{-}P}$ = 0.53 Å
Interatomic N-P distance, $R_{N\text{-}P}$, Å

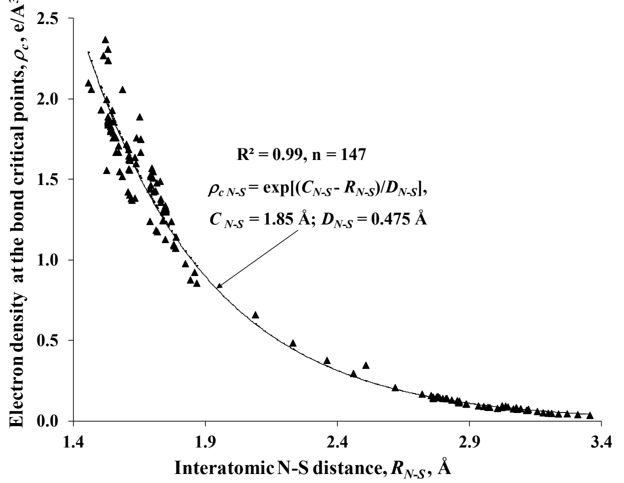
R² = 0.99, n = 147
$\rho_{c\,N\text{-}S} = \exp[(C_{N\text{-}S} - R_{N\text{-}S})/D_{N\text{-}S}]$,
$C_{N\text{-}S}$ = 1.85 Å; $D_{N\text{-}S}$ = 0.475 Å
Electron density at the bond critical points, ρ_c, e/A^3
Interatomic N-S distance, $R_{N\text{-}S}$, Å

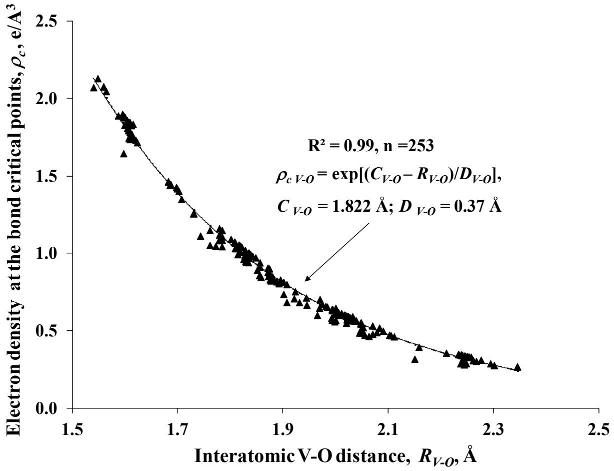
R² = 0.99, n =253
$\rho_{c\,V\text{-}O} = \exp[(C_{V\text{-}O} - R_{V\text{-}O})/D_{V\text{-}O}]$,
$C_{V\text{-}O}$ = 1.822 Å; $D_{V\text{-}O}$ = 0.37 Å
Electron density at the bond critical points, ρ_c, e/A^3
Interatomic V-O distance, $R_{V\text{-}O}$, Å

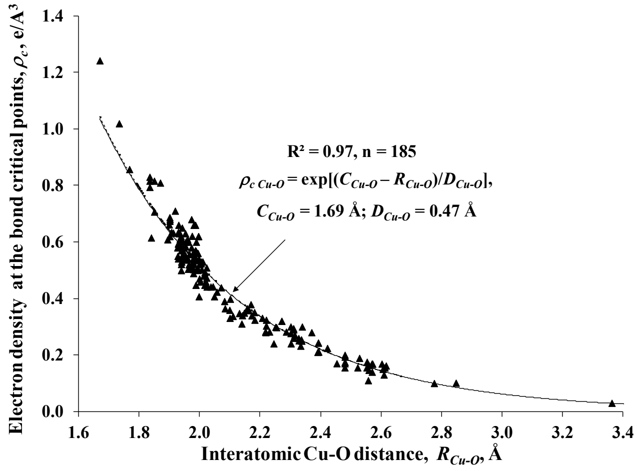
R² = 0.97, n = 185
$\rho_{c\,Cu\text{-}O} = \exp[(C_{Cu\text{-}O} - R_{Cu\text{-}O})/D_{Cu\text{-}O}]$,
$C_{Cu\text{-}O}$ = 1.69 Å; $D_{Cu\text{-}O}$ = 0.47 Å
Electron density at the bond critical points, ρ_c, e/A^3
Interatomic Cu-O distance, $R_{Cu\text{-}O}$, Å

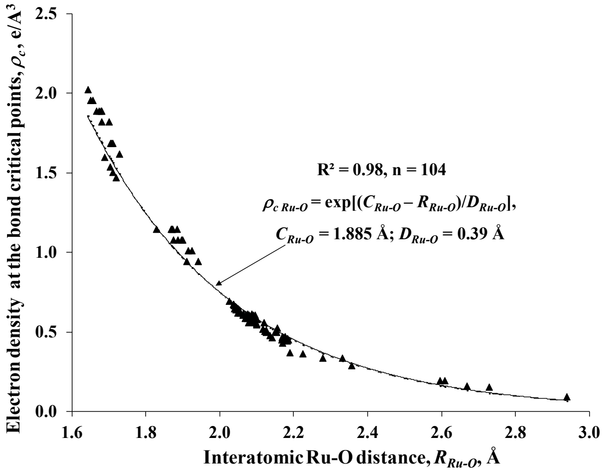
R² = 0.98, n = 104
$\rho_{c\,Ru\text{-}O} = \exp[(C_{Ru\text{-}O} - R_{Ru\text{-}O})/D_{Ru\text{-}O}]$,
$C_{Ru\text{-}O}$ = 1.885 Å; $D_{Ru\text{-}O}$ = 0.39 Å
Electron density at the bond critical points, ρ_c, e/A^3
Interatomic Ru-O distance, $R_{Ru\text{-}O}$, Å

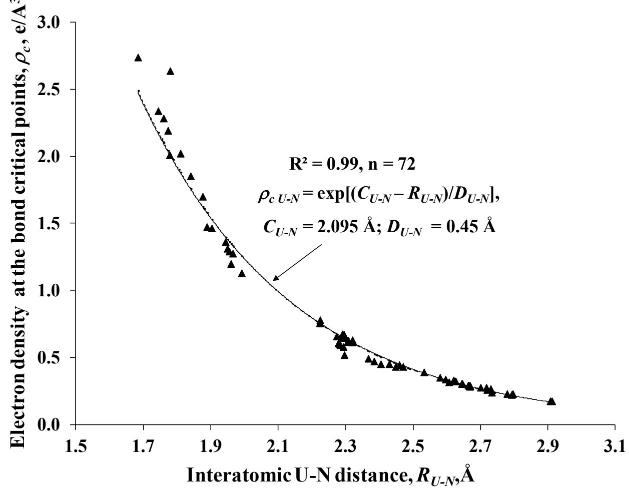
R² = 0.99, n = 72
$\rho_{c\,U\text{-}N} = \exp[(C_{U\text{-}N} - R_{U\text{-}N})/D_{U\text{-}N}]$,
$C_{U\text{-}N}$ = 2.095 Å; $D_{U\text{-}N}$ = 0.45 Å
Electron density at the bond critical points, ρ_c, e/A^3
Interatomic U-N distance, $R_{U\text{-}N}$,Å

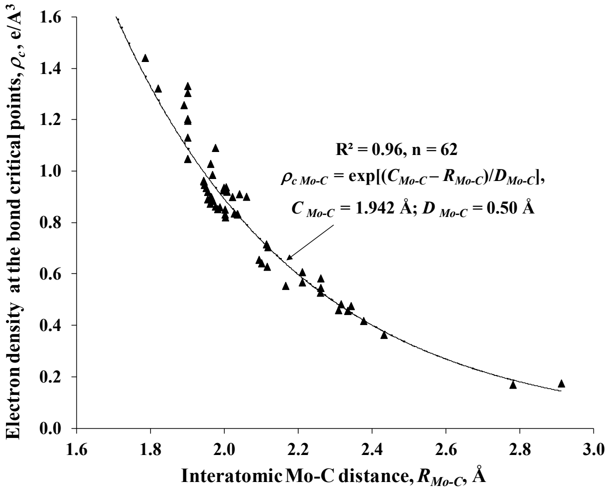
R² = 0.96, n = 62
$\rho_{c\,Mo\text{-}C} = \exp[(C_{Mo\text{-}C} - R_{Mo\text{-}C})/D_{Mo\text{-}C}]$,
$C_{Mo\text{-}C}$ = 1.942 Å; $D_{Mo\text{-}C}$ = 0.50 Å
Electron density at the bond critical points, ρ_c, e/A^3
Interatomic Mo-C distance, $R_{Mo\text{-}C}$, Å

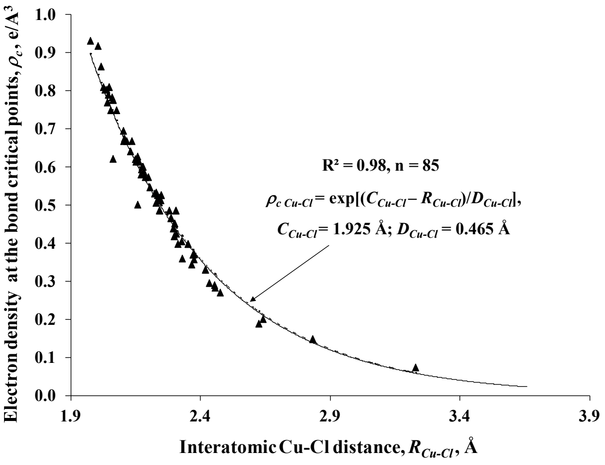
Electron density at the bottom critical points, ρ_c, e/A^3
Interatomic Cu-Cl distance, $R_{Cu\text{-}Cl}$, Å
$R^2 = 0.98$, n = 85
$\rho_{c\,Cu\text{-}Cl} = \exp[(C_{Cu\text{-}Cl} - R_{Cu\text{-}Cl})/D_{Cu\text{-}Cl}]$,
$C_{Cu\text{-}Cl}$ = 1.925 Å; $D_{Cu\text{-}Cl}$ = 0.465 Å

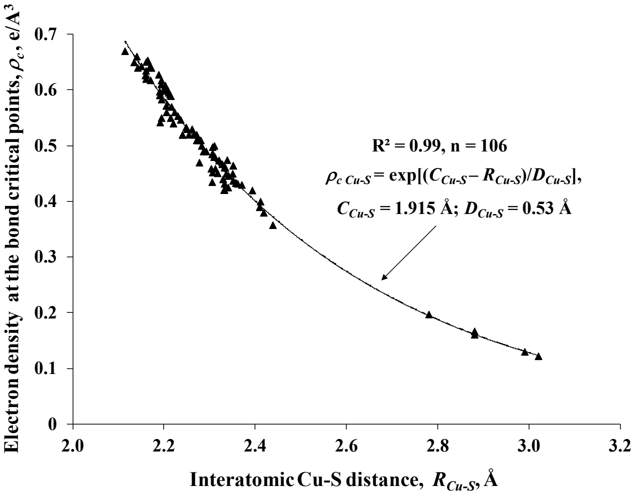
Electron density at the bond critical points, ρ_c, e/A^3
Interatomic Cu-S distance, $R_{Cu\text{-}S}$, Å
$R^2 = 0.99$, n = 106
$\rho_{c\,Cu\text{-}S} = \exp[(C_{Cu\text{-}S} - R_{Cu\text{-}S})/D_{Cu\text{-}S}]$,
$C_{Cu\text{-}S}$ = 1.915 Å; $D_{Cu\text{-}S}$ = 0.53 Å

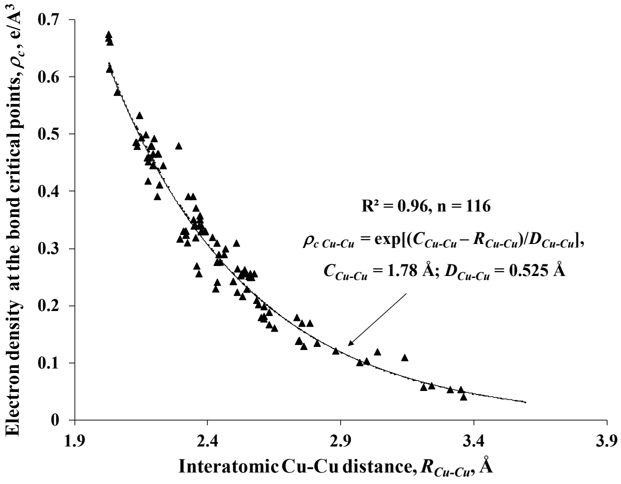
Electron density at the bond critical points, ρ_c, e/A^3
Interatomic Cu-Cu distance, $R_{Cu\text{-}Cu}$, Å
$R^2 = 0.96$, n = 116
$\rho_{c\,Cu\text{-}Cu} = \exp[(C_{Cu\text{-}Cu} - R_{Cu\text{-}Cu})/D_{Cu\text{-}Cu}]$,
$C_{Cu\text{-}Cu}$ = 1.78 Å; $D_{Cu\text{-}Cu}$ = 0.525 Å

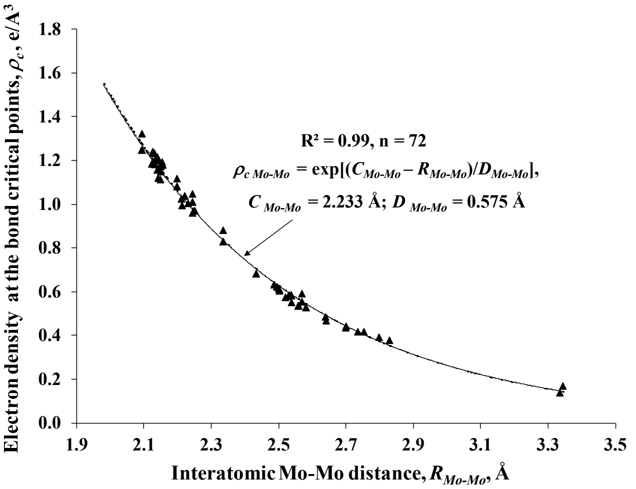
Electron density at the bond critical points, ρ_c, e/A^3
Interatomic Mo-Mo distance, $R_{Mo\text{-}Mo}$, Å
$R^2 = 0.99$, n = 72
$\rho_{c\,Mo\text{-}Mo} = \exp[(C_{Mo\text{-}Mo} - R_{Mo\text{-}Mo})/D_{Mo\text{-}Mo}]$,
$C_{Mo\text{-}Mo}$ = 2.233 Å; $D_{Mo\text{-}Mo}$ = 0.575 Å

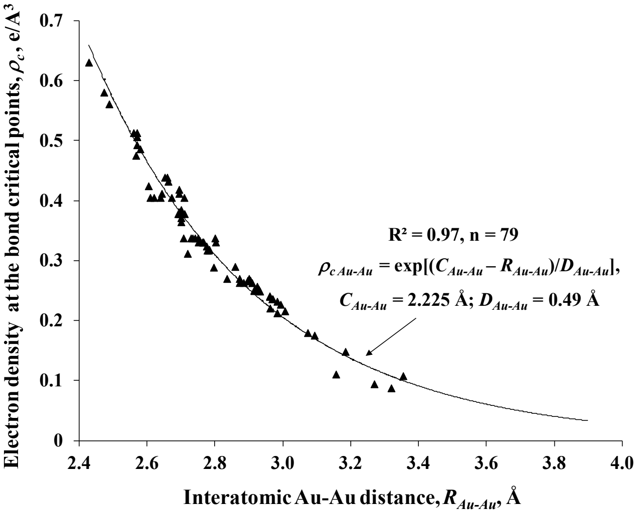
Electron density at the bond critical points, ρ_c, e/A^3
Interatomic Au-Au distance, $R_{Au\text{-}Au}$, Å
$R^2 = 0.97$, n = 79
$\rho_{c\,Au\text{-}Au} = \exp[(C_{Au\text{-}Au} - R_{Au\text{-}Au})/D_{Au\text{-}Au}]$,
$C_{Au\text{-}Au}$ = 2.225 Å; $D_{Au\text{-}Au}$ = 0.49 Å

Figure S3. Exponential correlations between effective bond order, BO_{ij}, and the interatomic distances. The points are the quantum chemistry data available in literature. The crystal chemistry curves marked in red are presented for comparison.

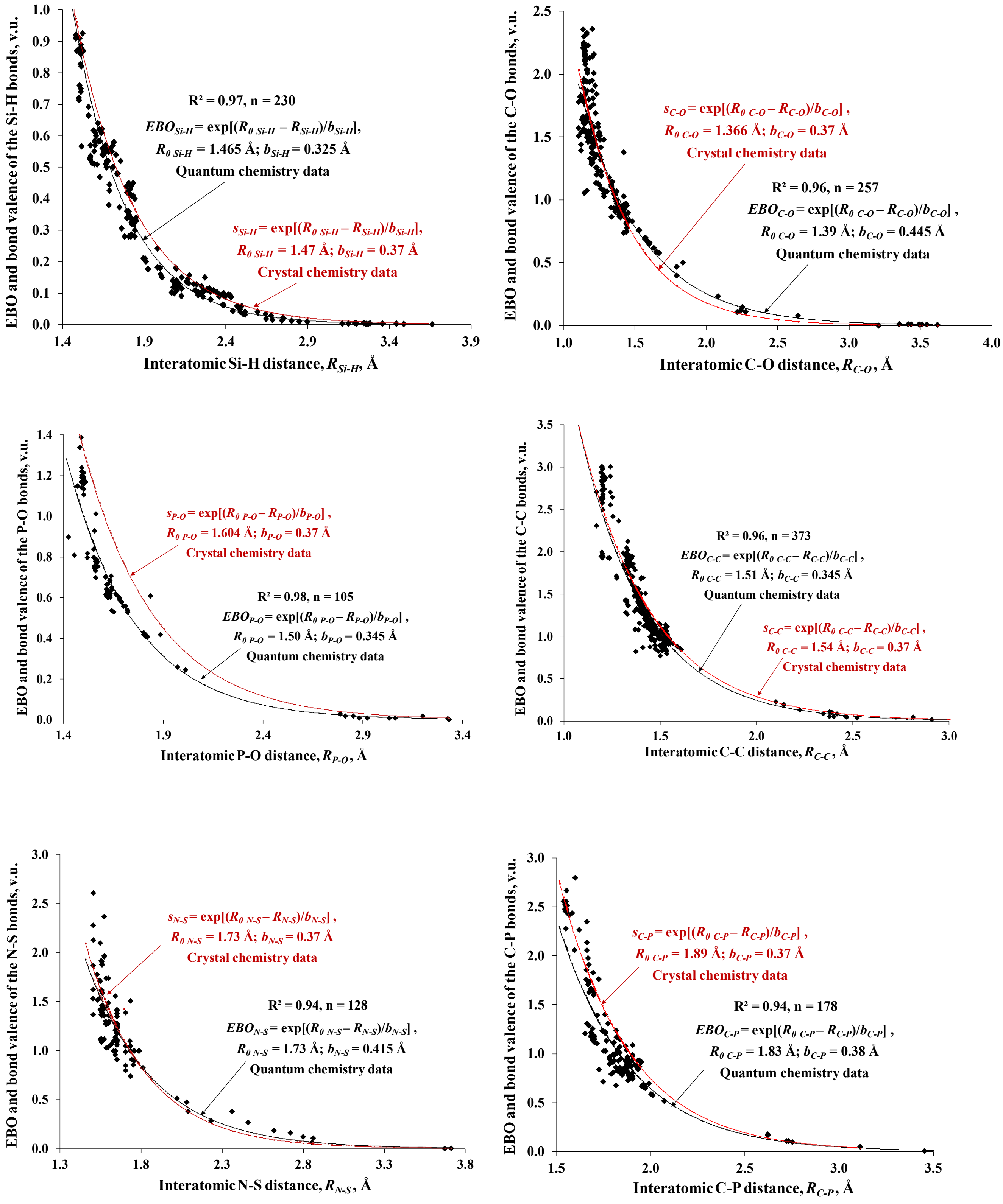

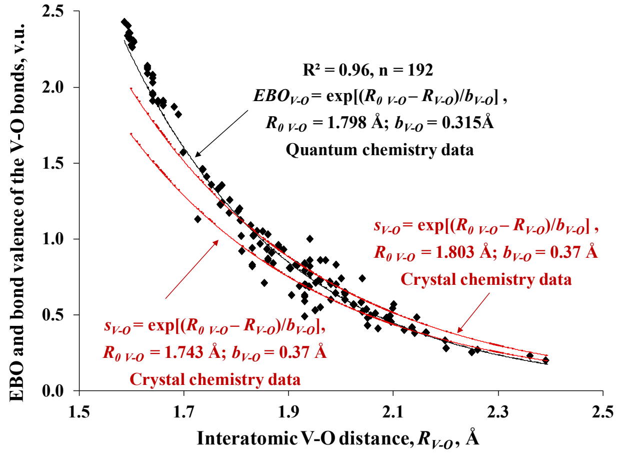

R^2 = 0.96, n = 192
$EBO_{V\text{-}O} = \exp[(R_{0\ V\text{-}O} - R_{V\text{-}O})/b_{V\text{-}O}]$,
$R_{0\ V\text{-}O}$ = 1.798 Å; $b_{V\text{-}O}$ = 0.315Å
Quantum chemistry data
$s_{V\text{-}O} = \exp[(R_{0\ V\text{-}O} - R_{V\text{-}O})/b_{V\text{-}O}]$,
$R_{0\ V\text{-}O}$ = 1.803 Å; $b_{V\text{-}O}$ = 0.37 Å
Crystal chemistry data
$s_{V\text{-}O} = \exp[(R_{0\ V\text{-}O} - R_{V\text{-}O})/b_{V\text{-}O}]$,
$R_{0\ V\text{-}O}$ = 1.743 Å; $b_{V\text{-}O}$ = 0.37 Å
Crystal chemistry data
EBO and bond valence of the V-O bonds, v.u.
Interatomic V-O distance, $R_{V\text{-}O}$, Å

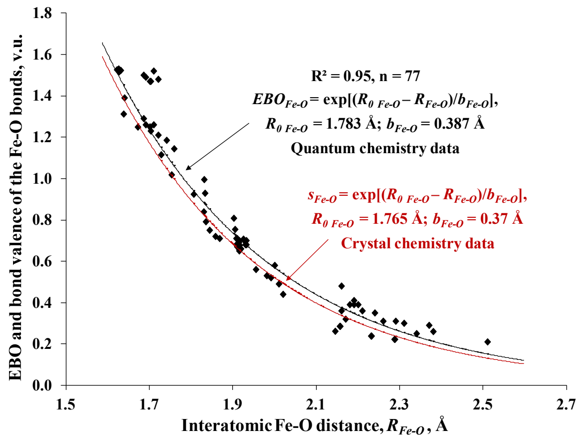

R^2 = 0.95, n = 77
$EBO_{Fe\text{-}O} = \exp[(R_{0\ Fe\text{-}O} - R_{Fe\text{-}O})/b_{Fe\text{-}O}]$,
$R_{0\ Fe\text{-}O}$ = 1.783 Å; $b_{Fe\text{-}O}$ = 0.387 Å
Quantum chemistry data
$s_{Fe\text{-}O} = \exp[(R_{0\ Fe\text{-}O} - R_{Fe\text{-}O})/b_{Fe\text{-}O}]$,
$R_{0\ Fe\text{-}O}$ = 1.765 Å; $b_{Fe\text{-}O}$ = 0.37 Å
Crystal chemistry data
EBO and bond valence of the Fe-O bonds, v.u.
Interatomic Fe-O distance, $R_{Fe\text{-}O}$, Å

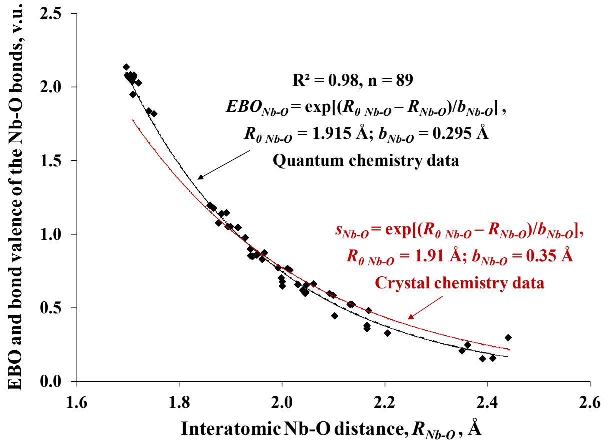

R^2 = 0.98, n = 89
$EBO_{Nb\text{-}O} = \exp[(R_{0\ Nb\text{-}O} - R_{Nb\text{-}O})/b_{Nb\text{-}O}]$,
$R_{0\ Nb\text{-}O}$ = 1.915 Å; $b_{Nb\text{-}O}$ = 0.295 Å
Quantum chemistry data
$s_{Nb\text{-}O} = \exp[(R_{0\ Nb\text{-}O} - R_{Nb\text{-}O})/b_{Nb\text{-}O}]$,
$R_{0\ Nb\text{-}O}$ = 1.91 Å; $b_{Nb\text{-}O}$ = 0.35 Å
Crystal chemistry data
EBO and bond valence of the Nb-O bonds, v.u.
Interatomic Nb-O distance, $R_{Nb\text{-}O}$, Å

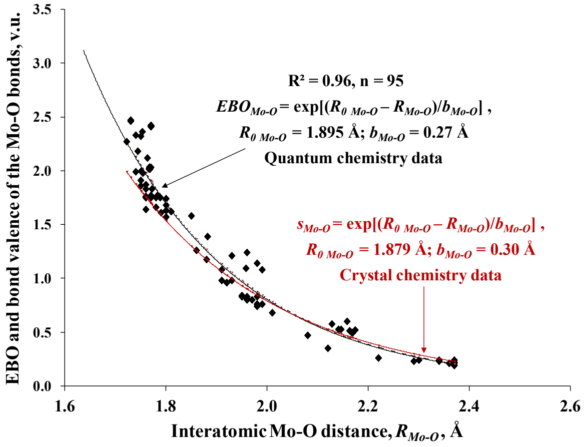

R^2 = 0.96, n = 95
$EBO_{Mo\text{-}O} = \exp[(R_{0\ Mo\text{-}O} - R_{Mo\text{-}O})/b_{Mo\text{-}O}]$,
$R_{0\ Mo\text{-}O}$ = 1.895 Å; $b_{Mo\text{-}O}$ = 0.27 Å
Quantum chemistry data
$s_{Mo\text{-}O} = \exp[(R_{0\ Mo\text{-}O} - R_{Mo\text{-}O})/b_{Mo\text{-}O}]$,
$R_{0\ Mo\text{-}O}$ = 1.879 Å; $b_{Mo\text{-}O}$ = 0.30 Å
Crystal chemistry data
EBO and bond valence of the Mo-O bonds, v.u.
Interatomic Mo-O distance, $R_{Mo\text{-}O}$, Å

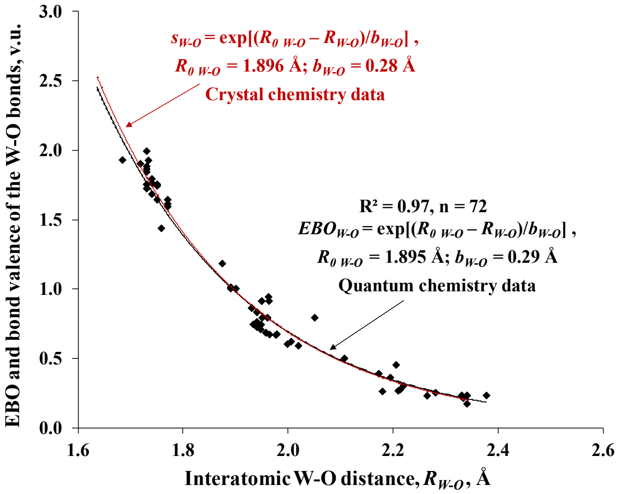

$s_{W\text{-}O} = \exp[(R_{0\ W\text{-}O} - R_{W\text{-}O})/b_{W\text{-}O}]$,
$R_{0\ W\text{-}O}$ = 1.896 Å; $b_{W\text{-}O}$ = 0.28 Å
Crystal chemistry data
R^2 = 0.97, n = 72
$EBO_{W\text{-}O} = \exp[(R_{0\ W\text{-}O} - R_{W\text{-}O})/b_{W\text{-}O}]$,
$R_{0\ W\text{-}O}$ = 1.895 Å; $b_{W\text{-}O}$ = 0.29 Å
Quantum chemistry data
EBO and bond valence of the W-O bonds, v.u.
Interatomic W-O distance, $R_{W\text{-}O}$, Å

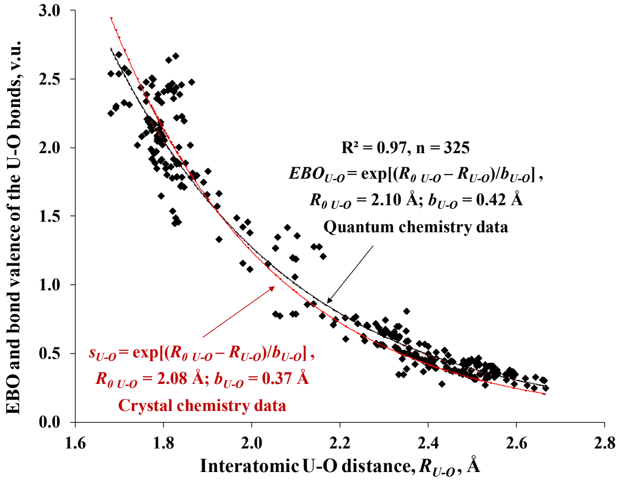

R^2 = 0.97, n = 325
$EBO_{U\text{-}O} = \exp[(R_{0\ U\text{-}O} - R_{U\text{-}O})/b_{U\text{-}O}]$,
$R_{0\ U\text{-}O}$ = 2.10 Å; $b_{U\text{-}O}$ = 0.42 Å
Quantum chemistry data
$s_{U\text{-}O} = \exp[(R_{0\ U\text{-}O} - R_{U\text{-}O})/b_{U\text{-}O}]$,
$R_{0\ U\text{-}O}$ = 2.08 Å; $b_{U\text{-}O}$ = 0.37 Å
Crystal chemistry data
EBO and bond valence of the U-O bonds, v.u.
Interatomic U-O distance, $R_{U\text{-}O}$, Å

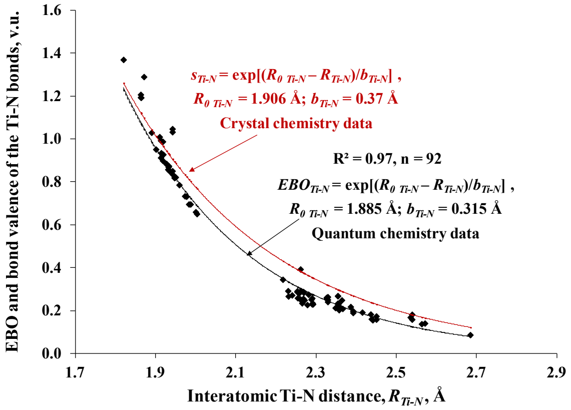

s_{Ti-N} = exp[($R_{0\ Ti-N}$ – R_{Ti-N})/b_{Ti-N}] ,
$R_{0\ Ti-N}$ = 1.906 Å; b_{Ti-N} = 0.37 Å
Crystal chemistry data
R^2 = 0.97, n = 92
EBO_{Ti-N} = exp[($R_{0\ Ti-N}$ – R_{Ti-N})/b_{Ti-N}] ,
$R_{0\ Ti-N}$ = 1.885 Å; b_{Ti-N} = 0.315 Å
Quantum chemistry data
EBO and bond valence of the Ti-N bonds, v.u.
Interatomic Ti-N distance, R_{Ti-N}, Å

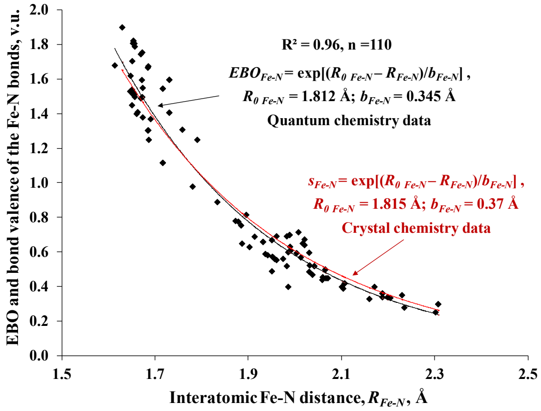

R^2 = 0.96, n =110
EBO_{Fe-N} = exp[($R_{0\ Fe-N}$ – R_{Fe-N})/b_{Fe-N}] ,
$R_{0\ Fe-N}$ = 1.812 Å; b_{Fe-N} = 0.345 Å
Quantum chemistry data
s_{Fe-N} = exp[($R_{0\ Fe-N}$ – R_{Fe-N})/b_{Fe-N}] ,
$R_{0\ Fe-N}$ = 1.815 Å; b_{Fe-N} = 0.37 Å
Crystal chemistry data
EBO and bond valence of the Fe-N bonds, v.u.
Interatomic Fe-N distance, R_{Fe-N}, Å

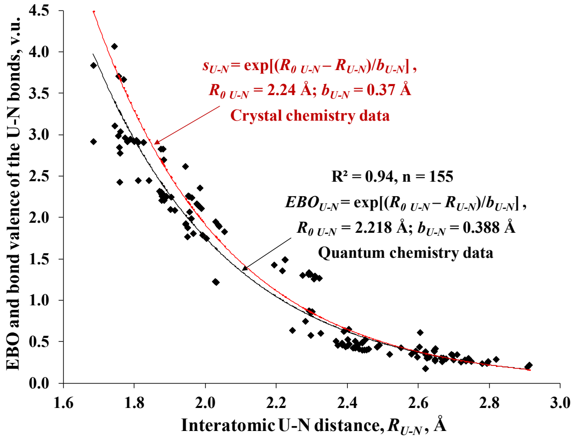

s_{U-N} = exp[($R_{0\ U-N}$ – R_{U-N})/b_{U-N}] ,
$R_{0\ U-N}$ = 2.24 Å; b_{U-N} = 0.37 Å
Crystal chemistry data
R^2 = 0.94, n = 155
EBO_{U-N} = exp[($R_{0\ U-N}$ – R_{U-N})/b_{U-N}] ,
$R_{0\ U-N}$ = 2.218 Å; b_{U-N} = 0.388 Å
Quantum chemistry data
EBO and bond valence of the U-N bonds, v.u.
Interatomic U-N distance, R_{U-N}, Å

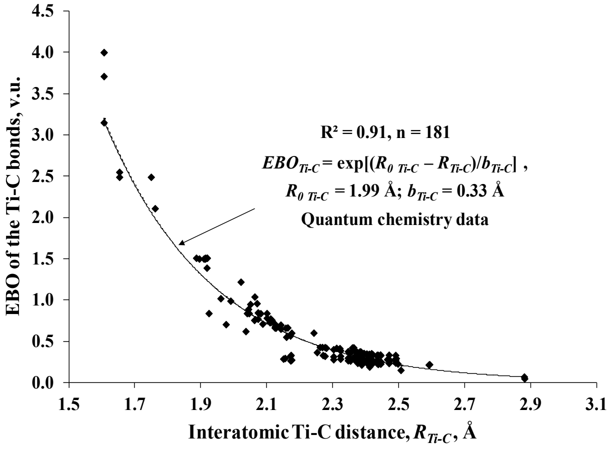

R^2 = 0.91, n = 181
EBO_{Ti-C} = exp[($R_{0\ Ti-C}$ – R_{Ti-C})/b_{Ti-C}] ,
$R_{0\ Ti-C}$ = 1.99 Å; b_{Ti-C} = 0.33 Å
Quantum chemistry data
EBO of the Ti-C bonds, v.u.
Interatomic Ti-C distance, R_{Ti-C}, Å

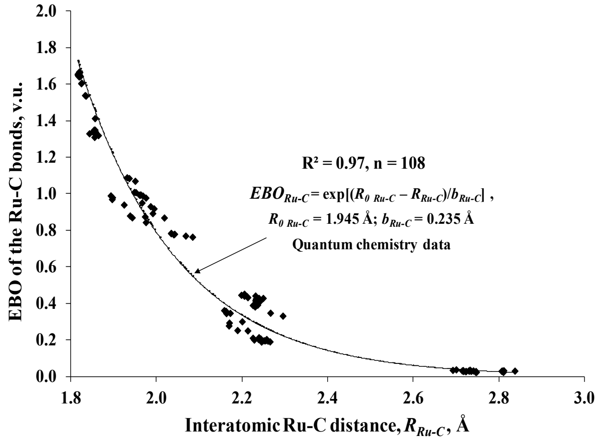

R^2 = 0.97, n = 108
EBO_{Ru-C} = exp[($R_{0\ Ru-C}$ – R_{Ru-C})/b_{Ru-C}] ,
$R_{0\ Ru-C}$ = 1.945 Å; b_{Ru-C} = 0.235 Å
Quantum chemistry data
EBO of the Ru-C bonds, v.u.
Interatomic Ru-C distance, R_{Ru-C}, Å

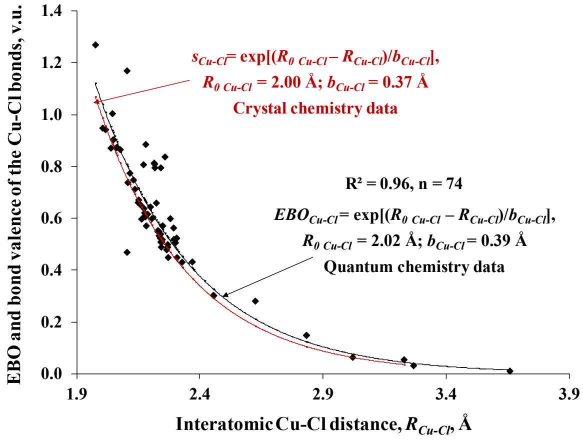

s_{Cu-Cl} = exp[($R_{0\ Cu-Cl}$ – R_{Cu-Cl})/b_{Cu-Cl}],
$R_{0\ Cu-Cl}$ = 2.00 Å; b_{Cu-Cl} = 0.37 Å
Crystal chemistry data
R^2 = 0.96, n = 74
EBO_{Cu-Cl} = exp[($R_{0\ Cu-Cl}$ – R_{Cu-Cl})/b_{Cu-Cl}],
$R_{0\ Cu-Cl}$ = 2.02 Å; b_{Cu-Cl} = 0.39 Å
Quantum chemistry data
EBO and bond valence of the Cu-Cl bonds, v.u.
Interatomic Cu-Cl distance, R_{Cu-Cl}, Å

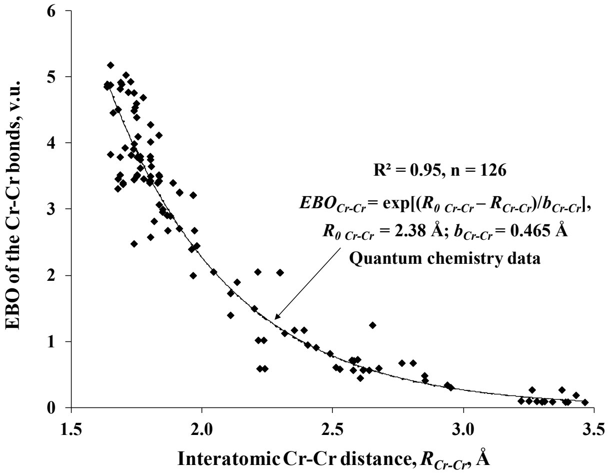
R² = 0.95, n = 126
$EBO_{Cr\text{-}Cr} = \exp[(R_{0\ Cr\text{-}Cr} - R_{Cr\text{-}Cr})/b_{Cr\text{-}Cr}]$,
$R_{0\ Cr\text{-}Cr}$ = 2.38 Å; $b_{Cr\text{-}Cr}$ = 0.465 Å
Quantum chemistry data
EBO of the Cr-Cr bonds, v.u.
Interatomic Cr-Cr distance, $R_{Cr\text{-}Cr}$, Å

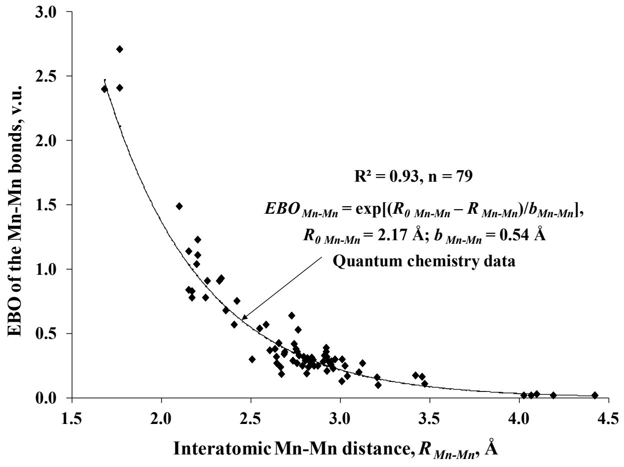
R² = 0.93, n = 79
$EBO_{Mn\text{-}Mn} = \exp[(R_{0\ Mn\text{-}Mn} - R_{Mn\text{-}Mn})/b_{Mn\text{-}Mn}]$,
$R_{0\ Mn\text{-}Mn}$ = 2.17 Å; $b_{Mn\text{-}Mn}$ = 0.54 Å
Quantum chemistry data
EBO of the Mn-Mn bonds, v.u.
Interatomic Mn-Mn distance, $R_{Mn\text{-}Mn}$, Å

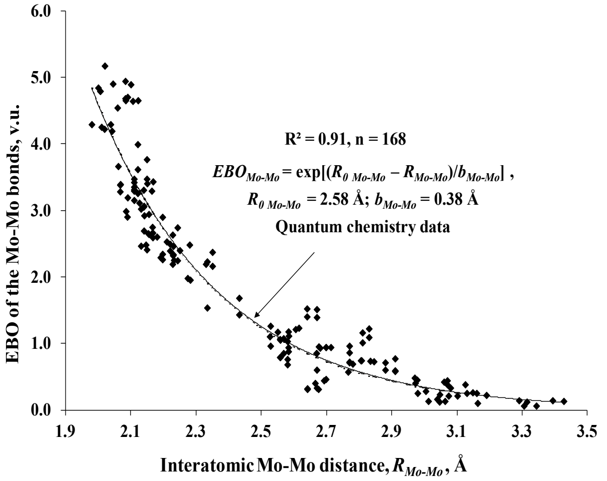
R² = 0.91, n = 168
$EBO_{Mo\text{-}Mo} = \exp[(R_{0\ Mo\text{-}Mo} - R_{Mo\text{-}Mo})/b_{Mo\text{-}Mo}]$,
$R_{0\ Mo\text{-}Mo}$ = 2.58 Å; $b_{Mo\text{-}Mo}$ = 0.38 Å
Quantum chemistry data
EBO of the Mo-Mo bonds, v.u.
Interatomic Mo-Mo distance, $R_{Mo\text{-}Mo}$, Å

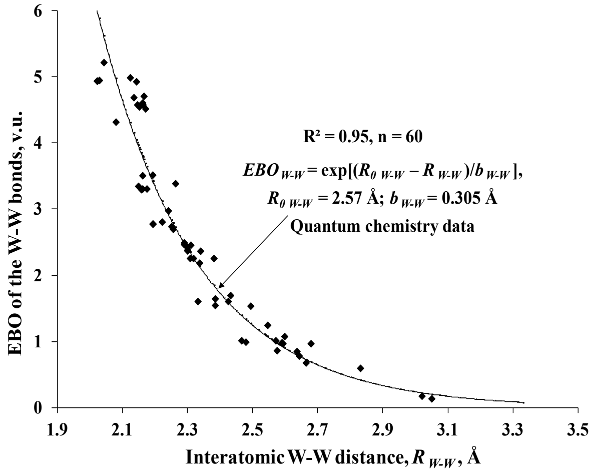
R² = 0.95, n = 60
$EBO_{W\text{-}W} = \exp[(R_{0\ W\text{-}W} - R_{W\text{-}W})/b_{W\text{-}W}]$,
$R_{0\ W\text{-}W}$ = 2.57 Å; $b_{W\text{-}W}$ = 0.305 Å
Quantum chemistry data
EBO of the W-W bonds, v.u.
Interatomic W-W distance, $R_{W\text{-}W}$, Å

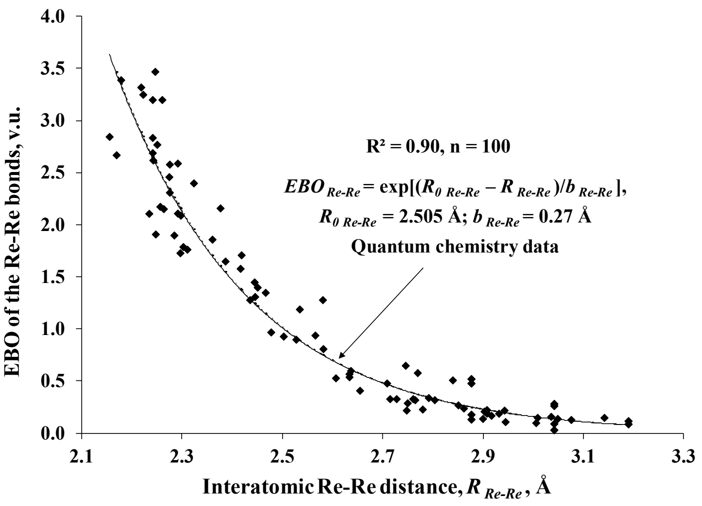
R² = 0.90, n = 100
$EBO_{Re\text{-}Re} = \exp[(R_{0\ Re\text{-}Re} - R_{Re\text{-}Re})/b_{Re\text{-}Re}]$,
$R_{0\ Re\text{-}Re}$ = 2.505 Å; $b_{Re\text{-}Re}$ = 0.27 Å
Quantum chemistry data
EBO of the Re-Re bonds, v.u.
Interatomic Re-Re distance, $R_{Re\text{-}Re}$, Å

Figure S4. Correlation between the *BO* parameter $R_{0\ ij}$ and electron density constant *Cij*.

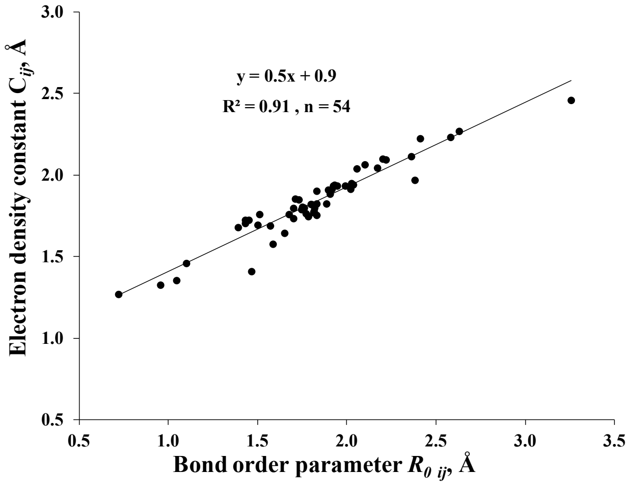

Figure S5. Bond order parameter $R_{0\ ij}$ (A) and electron density constant *Cij* (B) *vs*. the sum of the covalent radii of *i* and j atoms.

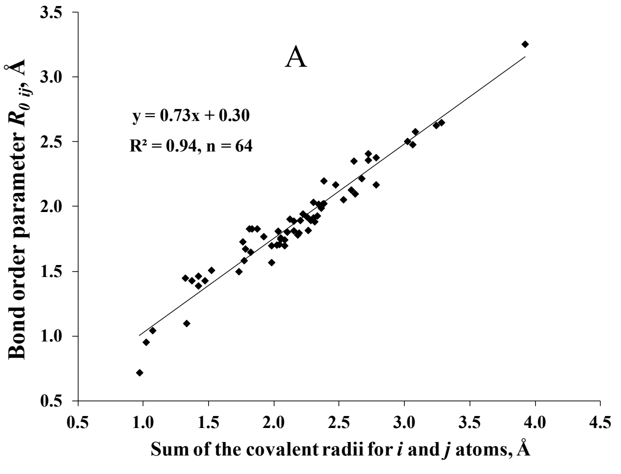

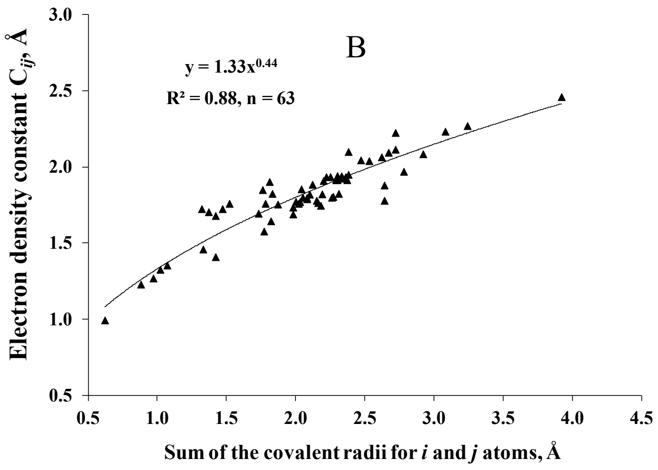

Figure S6. Relationship between electron density and bond order for some atom pairs.

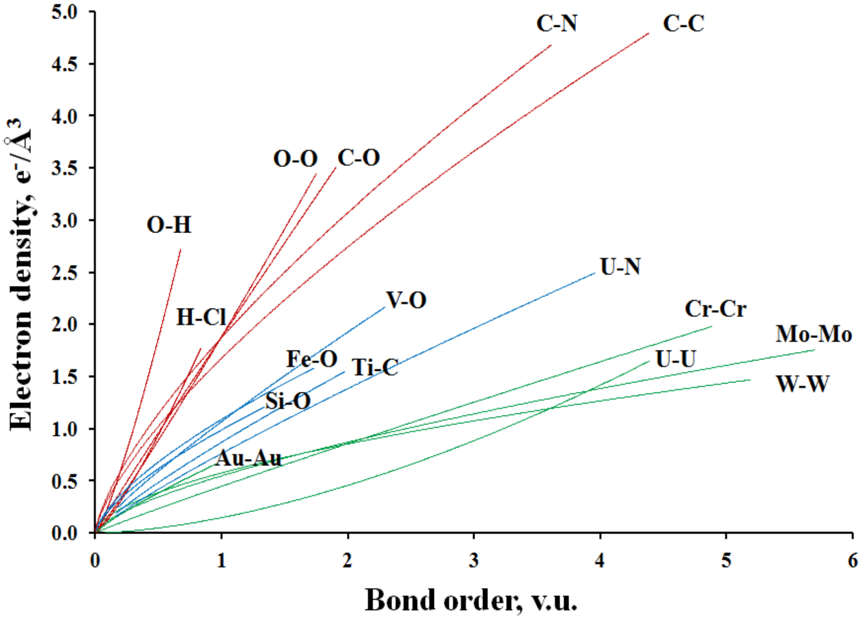

Figure S7. Additional parameters of the ED topology, which are not related to the Pauling's principles. A - Laplacian of the electron density as a function of the interatomic distance for the C-C, Fe-C, Cr-Cr and Fe-O bonds. B - Energy densities of the Cr-Cr bonds as a function of their *BOs*.

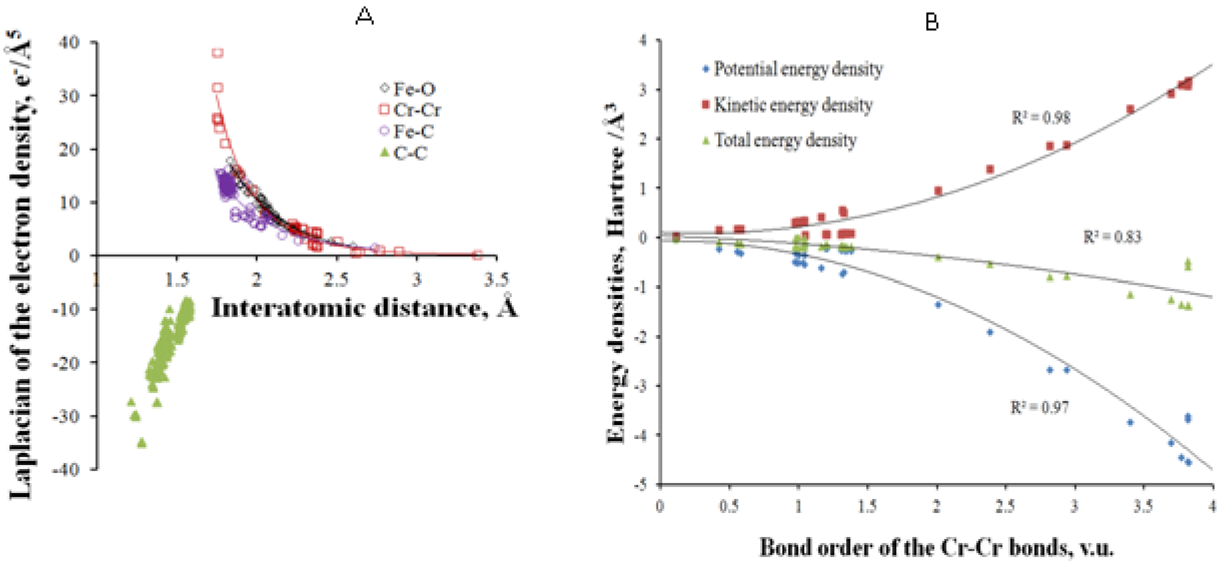

These parameters are widely used to describe the differences in the nature of chemical bonds. For example, as can be seen in Fig. S7A, in contrast to ionic or mixed ionic/covalent interactions, the Laplacian of the pure covalent C-C bonds is clearly negative. The key parameter to define the bond character is then the ratio between absolute value of the potential and kinetic energy

densities at the bond critical point [2], which is in turn related to the Laplacian value at this same position by the local expression of the quantum virial theorem [3]. For the ionic bonds this ratio is smaller then 1 (Laplacian >0), for the pure covalent bonds the ratio is larger than 2 (Laplacian <0), while the mixed interactions have the ratio between 1 and 2 (Laplacian >0). According to this parameter, the Cr-Cr interactions can be characterized as mixed ionic/covalent ones (Fig. S7B).

Figure S8. Linear correlation between the *BOs* of the Mo-O bonds calculated by Eq. 1 and the square of the stretching frequencies of various molybdates obtained in Raman spectroscopy. (The interatomic distances and stretching frequencies are taken from ref.[4])

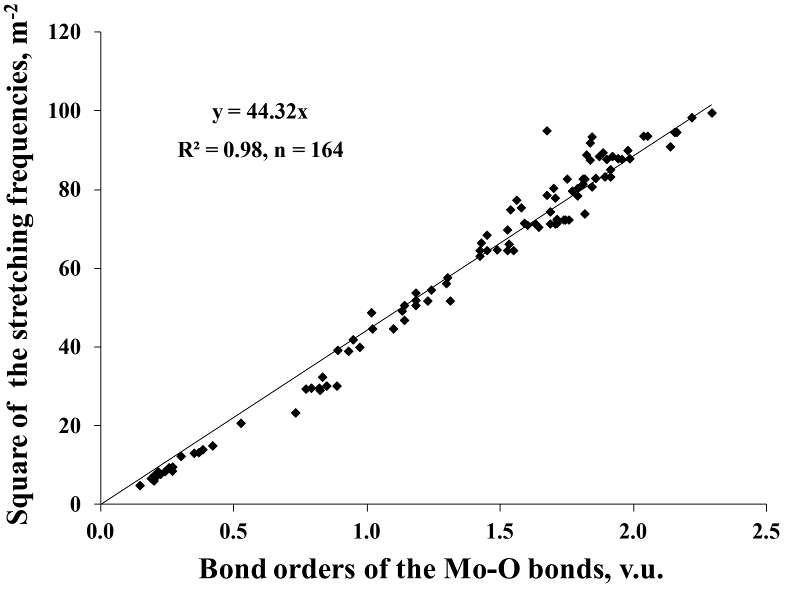

Table S1. The results of the exponential fitting of the quantum chemistry data by Eqs 1 and 3: Bond valence parameters and electron density constants for typical atomic pairs. *n* is a number of data points.

N	Bond	Bond valence parameters, Å		Quality of exponential fitting		Electron density constants, e/Å^3		Quality of exponential fitting		Ref.
		$R_{0\,ij}$	b_{ij}	R^2	*n*	C_{ij}	D_{ij}	R^2	*n*	
1	H-H					0.995	0.44	0.99	246	[5]
2	H-F					1.23	0.39	0.97	268	[2b, 5b, 5o, 6]
3	O-H	0.72	0.46	0.96	187	1.27	0.37	0.98	427	[5b, 5f, 7]
4	N-H	0.955	0.41	0.96	126	1.327	0.415	0.97	318	[5b, 5e, 7a, 7e, 7g, 7k, 7m, 7p, 7r-u, 7x, 7ae, 8]
5	C-H	1.045	0.365	0.98	75	1.355	0.435	0.99	398	[5b, 5d-f, 5o, 7s, 8a, 8b, 8i, 9]
6	Si-H	1.465	0.325	0.97	230	1.41	0.475	0.98	147	[8u, 10]
7	H-Cl	1.10	0.56	0.93	125	1.46	0.455	0.98	198	[5m, 6b, 6d, 7m, 7s, 8n, 8p, 8s, 8u, 11]
8	C-O	1.39	0.445	0.96	257	1.68	0.46	0.99	345	[5b, 5d, 5f, 7h, 7i, 7s, 8a, 8e, 8i, 9b, 9e, 9f, 9j, 9m, 12]
9	N-O	1.43	0.37	0.86	240	1.705	0.40	1.00	187	[5b, 7g, 7w, 7z, 8j, 8u, 9d, 9l, 11r, 12z, 12ae, 13]
10	Si-O	1.585	0.365	0.90	103	1.578	0.535	0.99	212	[6e, 8p, 8u, 10f, 10q, 10u, 10v, 13l, 14]
11	P-O	1.50	0.345	0.98	105	1.695	0.515	0.98	165	[8j, 12s, 13l, 14k, 14v, 15]
12	O-O	1.45	0.47	0.92	83	1.725	0.435	0.99	136	[5b, 5d, 7j, 7ab, 13m, 14d, 16]
13	Si-N	1.65	0.375	0.97	170	1.645	0.515	0.98	216	[8q, 10q, 10t, 10u, 14e, 14i, 14j, 14o, 14u, 14w, 14y, 17]
14	N-C	1.43	0.335	0.97	200	1.725	0.47	1.00	287	[5b, 5d, 7g-i, 7s, 8a-g, 8i, 8j, 9b, 9d, 9i, 9l, 9o, 12k, 12n, 12r, 12t, 12aj, 12am, 13e, 13k, 13o-q, 16n, 17f, 17l, 18]
15	C-C	1.51	0.345	0.96	373	1.76	0.485	0.99	786	[5b-d, 5f, 7h, 7i, 7p, 7s, 8a, 8e, 8i, 9d-g, 9i-m, 12d, 12j, 12k, 12n, 12r, 12t, 12al, 18c, 18e-g, 18x, 19]
16	Si-C	1.83	0.37	0.90	263	1.755	0.495	0.96	185	[10f, 10i, 10o, 10p, 10u-w, 10z, 14a, 14f, 14i, 14j, 17j, 17p, 17t,

										18p, 19o, 20]
17	N-Cl	1.71	0.585	0.93	66	1.855	0.455	0.98	124	[11d, 11n, 11r, 13m, 21]
18	N-P	1.675	0.335	0.95	157	1.76	0.53	0.99	178	[8k, 8o, 12aj, 14i, 15c, 15e, 15l, 15p, 17g, 17m, 18m, 18t, 22]
19	N-S	1.73	0.415	0.94	128	1.85	0.475	0.99	147	[5d, 8b, 8j, 14i, 15e, 16j, 23]
20	C-P	1.83	0.38	0.94	178	1.825	0.58	0.94	225	[8j, 8k, 9g, 9i, 9m, 9o, 9q, 12n, 12o, 15l-o, 18j, 18m, 18t, 18v, 20ac, 22o, 22r, 24]
21	C-S	1.83	0.325	0.97	203	1.903	0.533	0.98	220	[5d, 5g, 7g, 7h, 8b, 8u, 12n, 12ac, 12al, 14k, 18g, 18i, 18l, 18s, 18v, 19m, 23k, 25]
22	Ti-O	1.818	0.29	0.93	141	1.80	0.385	0.95	51	[9g, 11f, 12g, 12s, 16p, 26]
23	V-O	1.798	0.315	0.96	192	1.822	0.37	0.99	253	[7i, 7w, 12ak, 13b, 16e, 16p, 23d, 26k, 27]
24	Cr-O	1.76	0.315	0.93	164	1.80	0.38	0.97	61	[16p, 26h, 26j, 26k, 28]
25	Mn-O	1.75	0.34	0.95	53	1.805	0.38	0.99	71	[12t, 16p, 26h, 26k, 29]
26	Fe-O	1.783	0.387	0.95	77	1.747	0.41	0.94	156	[12m, 16p, 26h, 28i, 28l, 29b, 29f, 29n, 30]
27	Co-O	1.77	0.315	0.97	42	1.765	0.385	0.96	99	[7u, 13w, 16p, 26k, 29c, 29f, 30i, 30n, 31]
28	Cu-O	1.57	0.47	0.87	149	1.69	0.47	0.97	185	[7u, 9d, 12t, 16a, 16b, 16p, 26k, 27g, 29b, 29h, 31c, 32]
29	Nb-O	1.915	0.295	0.98	89	1.915	0.39	0.95	34	[19l, 26k, 27a, 27h, 33]
30	Mo-O	1.895	0.27	0.96	95	1.91	0.37	0.99	58	[12x, 15a, 26k, 27a, 28h, 33c, 34]
31	Ru-O	1.905	0.355	0.95	46	1.885	0.39	0.98	104	[26k, 30k, 34c, 35]
32	W-O	1.895	0.29	0.97	72					[26j, 26k, 27a, 28l, 33c, 34h, 34m, 36]
33	U-O	2.10	0.42	0.97	325	2.065	0.42	0.97	151	[8b, 25e, 26l, 27a, 37]
34	Pu-O	2.055	0.385	0.98	38	2.04	0.39	0.99	34	[7f, 37m, 37q, 37v, 37aa, 38]
35	Ti-N	1.885	0.315	0.97	92	1.825	0.47	0.98	86	[26i-k, 39]
36	Cr-N	1.805	0.285	0.92	109	1.82	0.42	0.97	93	[26j, 26k, 28e, 28g, 28l, 40]
37	Fe-N	1.812	0.345	0.96	110	1.771	0.45	0.96	83	[13c, 13d, 13u, 26k, 30d, 30g, 30i, 30k, 30o, 30p, 41]

38	Co-N					1.76	0.46	0.95	139	[12j, 19k, 26k, 28l, 30i, 31b-e, 31i-l, 40f, 41n, 42]
39	Cu-N	1.70	0.395	0.94	134	1.735	0.475	0.91	158	[9d, 12t, 18c, 18h, 29h, 31c, 32g, 32h, 32m, 32r-u, 32z, 32ac-ah, 32ak-am, 34c, 40f, 41n, 43]
40	Mo-N	1.923	0.325	0.92	116	1.935	0.43	0.99	69	[12x, 12z, 19c, 26j, 26k, 34c, 34g, 34l, 36a, 44]
41	W-N	1.93	0.275	0.95	140	1.94	0.43	0.98	53	[12ag, 18b, 23f, 26k, 36a, 44f, 45]
42	U-N	2.218	0.388	0.94	155	2.095	0.45	0.99	72	[8b, 8g, 37f, 37h, 37n-q, 37w, 37x, 37ac, 37ad, 46]
43	Ti-C	1.99	0.33	0.91	181	1.935	0.39	0.95	96	[9a, 9g, 9h, 11f, 12g, 19i, 19p, 26e, 26i, 26j, 37n, 39c, 39e, 47]
44	Cr-C	1.89	0.345	0.90	157					[9a, 12d, 12f, 12j, 12w, 12aa, 12ah, 19e, 19f, 26j, 28g, 40e, 47a, 48]
45	Mn-C	1.815	0.315	0.95	47	1.78	0.485	0.94	126	[9j, 9k, 9m, 12i, 12u, 12x, 19f, 19l, 47a, 49]
46	Fe-C	1.745	0.36	0.90	146	1.79	0.53	0.95	188	[9c, 9k, 12d, 12f, 12h, 12i, 12m, 12aa, 19f, 22b, 41i, 47a, 47b, 47g, 47j, 48f, 50]
47	Co-C	1.705	0.46	0.89	70					[9f, 12c, 12d, 31e, 42i, 47a, 47i, 47j, 49d, 50g, 51]
48	Ni-C					1.773	0.48	0.97	77	[10i, 12aa, 48f, 52]
49	Cu-C	1.70	0.347	0.82	122	1.798	0.46	0.98	42	[12d, 18c, 18d, 32ac, 43b, 43d, 43h, 43m, 48i, 49d, 53]
50	Mo-C	2.035	0.365	0.94	109	1.942	0.50	0.96	62	[12v-z, 12ah, 19c, 26j, 34b, 44e, 47g, 48g, 54]
51	Ru-C	1.945	0.235	0.97	108	1.935	0.45	0.93	95	[12ab, 22b, 35c, 49e, 55]
52	W-C	2.025	0.29	0.91	230	1.95	0.465	0.92	85	[12ag, 12ah, 26j, 28g, 36b, 45c, 48g, 50c, 54f, 54l, 54m, 55f, 56]
53	U-C	2.36	0.475	0.91	162	2.115	0.46	0.88	52	[37c, 37n, 46g, 46h, 46k, 46q, 47c, 54b, 57]
54	Cu-Cl	2.02	0.39	0.96	74	1.925	0.465	0.98	85	[32ag, 43f, 43h, 43k, 53j, 58]
55	Ag-Cl	2.17	0.43	0.96	41	2.045	0.475	0.99	41	[53j, 58b, 58d]
56	Au-Cl	2.20	0.42	0.97	55	2.10	0.52	1.00	53	[11q, 53j, 58b, 58d, 58e, 59]
57	Fe-P	2.13	0.485	0.90	41					[9c, 12i, 12w, 22b, 41b, 41d, 41g, 50i, 50m, 50n, 60]

58	Mo-P	2.353	0.29	0.93	52					[12v-z, 15a, 44b, 44g, 54c, 54j, 61]
59	Ni-S					1.915	0.55	0.91	43	[25i, 28l, 52d, 62]
60	Cu-S	2.02	0.38	0.92	47	1.915	0.53	0.99	106	[18h, 32ah, 40f, 58a, 63]
61	V-V	2.48	0.395	0.93	61					[49d, 64]
62	Cr-Cr	2.38	0.465	0.95	126	1.97	0.49	0.91	38	[40c, 48g, 49d, 64e-g, 65]
63	Mn-Mn	2.17	0.54	0.93	79					[9j, 12i, 12q, 12u, 49a, 49b, 49d, 64e, 64f, 65i, 65w, 65ac, 66]
64	Fe-Fe					1.88	0.60	0.97	41	[12h, 12i, 13d, 49b, 49d, 50j, 64e-g, 65a, 65i, 66f, 67]
65	Cu-Cu					1.78	0.525	0.96	116	[32f, 32k, 32z, 49d, 53e, 58b-d, 59c, 63f, 68]
66	Nb-Nb	2.65	0.408	0.88	41					[49d, 64g, 64h, 67c, 69]
67	Mo-Mo	2.58	0.38	0.91	168	2.233	0.575	0.99	72	[12v, 12y, 34c, 34e, 34f, 34j, 34l, 34o, 48g, 49d, 54c, 54n, 54o, 61, 64g, 65m, 65n, 65p, 65v, 65z, 67c, 69b, 70]
68	Ru-Ru					2.085	0.58	0.96	43	[34c, 49d, 55a, 55c, 55h, 65b, 67c, 69b, 71]
69	W-W	2.628	0.375	0.96	81	2.27	0.65	0.99	18	[12ag, 36c, 48g, 56e, 56h, 64g, 65n, 65v, 65z, 70c, 70e, 70k, 70l, 70v, 72]
70	Re-Re	2.505	0.27	0.90	100					[48g, 65n, 65r, 70s, 70v, 73]
71	Au-Au	2.41	0.41	0.91	49	2.225	0.49	0.97	79	[42k, 53n, 58b, 58d, 59c, 59d, 68h, 74]
72	U-U	3.255	0.658	0.86	95	2.46	0.44	0.96	25	[37e, 37g, 46r, 57d, 65z, 70k, 75]

Table S2. The results of the *BO* calculations for the Re_6-cluster complexes.

N	Compound	VEC*	Bond length, Å			Bond order, v.u.			Total electron count for Re, v.u.		Cryst. Struct. ref.
			Re-Re	Re-L^i	Re-L^a	Re-Re	Re-L^i	Re-L^a	Expect	Calcul	
1	$[Bu_4N][Re_6S_8Cl_4(py)_2]$	23	2.596	2.401	N: 2.161<br>Cl: 2.400	0.68	0.92	N: 0.58<br>Cl: 0.60	7	7.00	[76]
2	$[Ph_4P]_3[Re_6S_8(CN)_6]$	23	2.606	2.411	2.150	0.66	0.89	0.65	7	6.87	[77]
3	$Cs_6Re_6S_{15}$	24	2.605	2.403	2.451	0.66	0.92	0.80	7	7.10	[78]
4	$[Bu_4N]_4[Re_6S_8Br_6]$	24	2.592	2.395	2.595	0.69	0.94	0.68	7	7.19	[79]
5	$[Bu_4N]_4[Re_6S_8I_6]$	24	2.598	2.403	2.787	0.68	0.92	0.62	7	7.00	[79]
6	$[Re_6S_8(PEt_3)_4(Br)_2]$	24	2.599	2.404	P: 2.466<br>Br: 2.550	0.67	0.91	P: 0.98<br>Br: 0.76	7	7.26	[80]
7	$[Re_6S_8(PEt_3)_6]Br_2*CH_2Cl_2$	24	2.614	2.405	2.483	0.64	0.91	0.94	7	7.13	[80]
8	$Cs_3Na[Re_6S_8(CN)_6]$	24	2.602	2.411	2.119	0.67	0.90	0.71	7	6.97	[81]
9	$[Ph_4P]_4[Re_6S_8(CN)_6]*(CH_3CN)_2$	24	2.608	2.404	2.166	0.65	0.91	0.62	7	6.88	[82]
10	$[Bu_4N]_4[Re_6S_8Cl_6]$	24	2.603	2.404	2.450	0.66	0.91	0.55	7	6.86	[79]
11	$[Bu_4N]_2[Re_6S_8Cl_4(cpy)_2]$	24	2.593	2.400	N: 2.212<br>Cl: 2.423	0.69	0.92	N: 0.53<br>Cl: 0.59	7	7.03	[76]
12	$[Bu_4N][Re_6S_8Cl_3(py)_3]$	24	2.594	2.401	N: 2.187<br>Cl: 2.424	0.69	0.92	N: 0.57<br>Cl: 0.59	7	7.02	[76]
13	$[Bu_4N]_2[Re_6S_8Cl_4(ppy)_2]$	24	2.598	2.410	N: 2.216<br>Cl: 2.435	0.68	0.90	N: 0.53<br>Cl: 0.57	7	6.86	[83]
14	$Cs_6Re_6Se_{15}$	24	2.634	2.523	2.574	0.59	0.94	0.82	7	6.95	[84]
15	$Re_6Se_8(PEt_3)_4(I)_2*2CHCl_3$	24	2.641	2.521	P: 2.481<br>I: 2.747	0.58	0.94	P: 0.95<br>I: 0.69	7	6.94	[85]
16	$Re_6Se_8(PEt_3)_4(CN)_2$	24	2.647	2.522	P: 2.478<br>C: 2.104	0.56	0.94	P: 0.95<br>C: 0.75	7	6.91	[86]
17	$[Re_6Se_8(PEt_3)_4(CO)_2][SbF_6]_2$	24	2.640	2.518	P: 2.484<br>C: 2.060	0.58	0.95	P: 0.94<br>C: 0.85	7	7.04	[87]
18	$Re_6Se_8(PEt_3)_5(CN)(BPh4)$	24	2.634	2.509	P: 2.478<br>C: 2.143	0.59	0.98	P: 0.95<br>C: 0.67	7	7.18	[86]
19	Re_6Te_{15}	24	2.676	2.692	2.710	0.51	1.02	0.98	7	7.09	[88]
20	$[Mn(salen)]_{4n}[Re_6Te_8(CN)_6]_n$	24	2.693	2.694	2.105	0.47	1.02	C: 0.75	7	6.71	[89]

* VEC = the total number of valence electrons of the cluster.

Table S3. Bond valence parameters used in this work.

i-j pair	Bond valence parameter, Å	
	$R_{0\ ij}$	b_{ij}
Re-Re	2.495	0.265
Re-N	1.98	0.37
Re-C	2.007	0.335
Re-Cl	2.23	0.37
Re-S	2.37	0.37
Re-Br	2.45	0.37
Re-P	2.46	0.37
Re-Se	2.50	0.37
Re-I	2.61	0.37

12. Electron descriptors calculated by Eqs 1 and 3 (Examples)

12.1. Electron density at the bond critical points in 4-[[4-(methoxy)-3-quinolinyl]thio]-3-thiomethylquinoline (See the fragment of the crystal structure)**.**

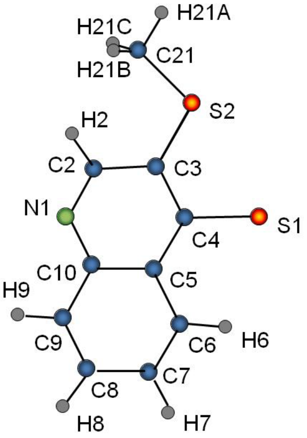

*Multipolar model.
**Independent Atom Model.

Bond	R_{ij}, Å	ρ_c, e/Å^3 Eq. 3 This work	ρ_c, e/Å^3 Ref. [90]		Relative difference, %	
			Model I*	Model II**	This work - Model I	Model I – Model II
S1-C4	1.782	1.25	1.28	1.01	2.3	21.1
S1-C13	1.770	1.28	1.27	1.03	0.8	18.9
S2-C3	1.758	1.31	1.30	1.05	0.8	19.2
S2-C21	1.808	1.20	1.26	0.98	4.8	22.2
C2-C3	1.428	1.98	2.00	1.41	1.0	29.5
C3-C4	1.390	2.14	2.12	1.50	0.9	29.2
C4-C5	1.426	1.99	1.96	1.41	1.5	28.1
C5-C6	1.421	2.01	2.00	1.43	0.5	28.5
C6-C7	1.376	2.20	2.23	1.53	1.3	31.4
C7-C8	1.416	2.03	2.07	1.44	1.9	30.4
C8-C9	1.378	2.20	2.18	1.53	0.9	29.8
C9-C10	1.417	2.03	2.06	1.43	1.5	30.6
N1-C2	1.317	2.38	2.53	1.86	5.9	26.5
N1-C10	1.367	2.14	2.19	1.71	2.3	21.9
N11-C12	1.317	2.38	2.52	1.85	5.6	26.6
N11-C20	1.374	2.11	2.13	1.69	0.9	20.7
C21-H21A	1.068	1.93	1.73	1.29	11.6	25.4
C21-H21B	0.986	2.34	1.84	1.45	27.2	21.2
C21-H21C	0.995	2.29	1.85	1.44	23.8	22.2
C2-H2	1.081	1.88	1.87	1.24	0.5	33.7
C6-H6	1.072	1.92	1.85	1.26	3.8	31.9
C7-H7	1.080	1.88	1.76	1.24	6.8	29.5
C8-H8	1.079	1.89	1.72	1.24	9.9	27.9
C9-H9	1.083	1.87	1.75	1.24	6.9	29.1
O1…H22B	2.020	0.13	0.04	0.04	225.0	0
H6…H16	2.201	0.08	0.08	0.03	0	62.5
O1…O1	3.178	0.06	0.04	0.03	50.0	25.0

12.2. Electron density at the bond critical points in 5,8,9,10-Tetrahydro-6H-1,3-dioxolo[4,5-h]pyrrolo[2,1-b]-[3]benzazepine-11-carbonitrile

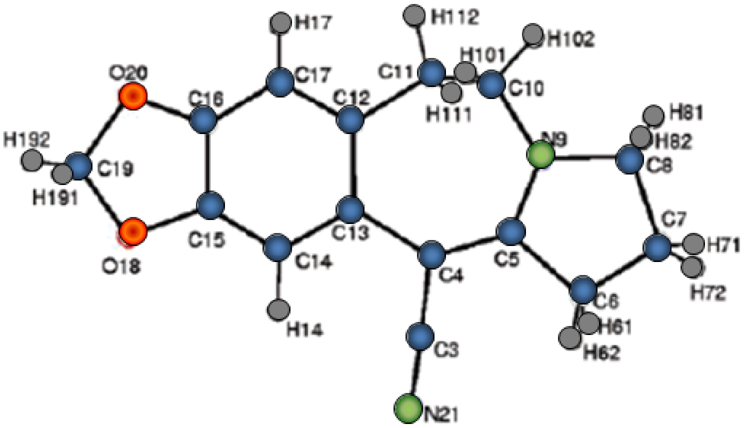

Bond	R_{ij}, Å	ρ_c, e/Å^3 Eq. 3 This work	ρ_c, e/Å^3 Ref. [91]	Relative difference, %
C19-O20	1.427	1.73	1.75	1.1
C19-O18	1.435	1.70	1.65	3.0
C16-O20	1.373	1.95	1.92	1.6
C15-O18	1.378	1.93	1.90	1.6
C15-C16	1.385	2.17	2.19	0.9
C17-C16	1.376	2.21	2.18	1..4
C14-C15	1.375	2.21	2.15	2.8
C14-C13	1.422	2.01	1.99	1.0
C17-C12	1.411	2.05	1.96	4.6
C13-C12	1.411	2.05	1.97	4.1
C12-C11	1.503	1.70	1.70	0
C4-C13	1.480	1.78	1.66	7.2
C4-C3	1.424	2.00	1.82	9.9
C4-C5	1.389	2.15	2.05	4.9
C10-C11	1.534	1.59	1.58	0.6
C6-C5	1.513	1.66	1.59	4.4
C6-C7	1.538	1.58	1.57	0.6
C8-C7	1.530	1.61	1.60	0.6
C3-N21	1.165	3.29	3.42	3.8
C10-N9	1.455	1.78	1.73	2.9
C8-N9	1.465	1.74	1.63	6.7
C5-N9	1.342	2.26	2.29	1.3

12.3. Electron density at the bond critical points in MeCp(CO)2Mn=C(OEt)Me.

The first and the second rows are related to the experimental and theoretical data, respectively, of the original work.

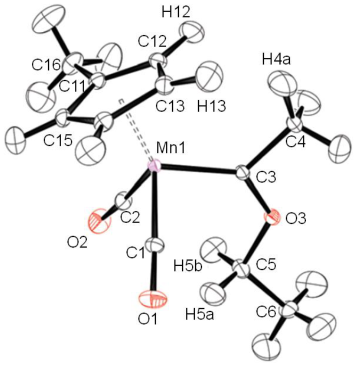

Bond	R_{ij}, Å	ρ_c, e/Å^3 Eq. 3 This work	ρ_c, e/Å^3 Ref.[92]	Relative difference, %
Mn1-C1	1.784	0.99	1.03	3.9
	1.781	1.00	1.03	2.9
Mn1-C2	1.780	1.00	1.01	1.0
	1.776	1.01	1.04	2.9
Mn1-C3	1.901	0.78	0.99	21.2
	1.900	0.78	0.89	12.4
Mn1-C11	2.167	0.45	0.41	9.8
	2.169	0.45	0.50	10.0
Mn1-C12	2.153	0.46	0.50	8.0
Mn1-C13	2.160	0.46	0.41	12.2
	2.151	0.47	0.49	4.1
Mn1-C14	2.160	0.46	0.40	15.0
	2.145	0.47	0.52	9.6
Mn1-C15	2.157	0.46	0.49	6.1
C1-O1	1.161	3.09	3.47	11.0
	1.158	3.11	3.09	0.6
C2-O2	1.164	3.07	3.49	12.0
	1.160	3.10	3.07	1.0
C3-C4	1.516	1.43	1.69	15.4
	1.508	1.45	1.78	18.5
C3-O3	1.334	2.12	2.15	1.4
	1.331	2.14	2.01	6.5
C5-O3	1.442	1.68	1.64	2.4
	1.436	1.70	1.63	4.3
C5-C6	1.508	1.68	1.77	5.1
	1.502	1.70	1.78	4.5
C11-C12	1.424	2.00	2.09	4.3
	1.417	2.03	2.01	1.0
C11-C15	1.430	1.97	2.07	4.8
	1.425	2.00	1.98	1.0
C11-C16	1.498	1.72	1.75	1.7
	1.487	1.76	1.80	2.2
C12-C13	1.427	1.99	2.01	1.0
	1.418	2.02	1.99	1.5
C13-C14	1.422	2.01	2.00	0.5
	1.417	2.03	2.00	1.5
C14-C15	1.420	2.02	2.04	1.0
	1.412	2.05	2.02	1.5

C12-H12a	1.083	1.87	1.82	2.7
	1.078	1.89	1.95	3.1
C13-H13a	1.083	1.87	1.86	0.5
	1.077	1.89	1.95	3.1
C14-H14a	1.083	1.87	1.85	1.1
	1.077	1.89	1.96	3.6
C15-H15a	1.083	1.87	1.87	0
	1.078	1.89	1.95	3.1
C16-H16a	1.093	1.83	1.78	2.8
	1.087	1.85	1.90	2.6
C16-H16b	1.093	1.83	1.76	4.0
	1.091	1.83	1.88	2.7
C16-H16c	1.093	1.83	1.74	5.2
	1.088	1.85	1.91	3.1
C1-H5a	2.410	0.09	0.08	12.5
	2.422	0.09	0.08	12.5
C2-H5b	2.530	0.07	0.09	22.2
	2.515	0.07	0.08	12.5
O1-H4a_4	2.520	0.03	0.02	50.0
O2-H5b_3	2.600	0.03	0.03	0
O2-H5a_3	2.515	0.03	0.02	50.0
H4a-O1_4	2.520	0.03	0.02	50.0
H5b-O2_3	2.600	0.03	0.03	0
H15a-O2_1	2.515	0.03	0.02	50.0
H4a-H12a	2.35	0.05	0.05	0
H4a-H13a	2.14	0.07	0.04	75.0
H4c-H6a_4	2.24	0.06	0.02	200.0
H5a-H12a_4	2.558	0.03	0.03	0
H6a-H4c_4	2.24	0.06	0.01	500.0
H6b-H15a_1	2.347	0.05	0.03	66.7
H6c-H13a_3	2.344	0.05	0.01	400.0
H16a-H16b_2	2.478	0.03	0.02	50.0
H16b-H16a_2	2.478	0.03	0.02	50.0
H13a-H6c_3	2.344	0.05	0.01	400.0
H15a-H6b_1	2.347	0.05	0.03	66.7

12.4. Bond orders in the W_2^{6+} paddlewheel complex.

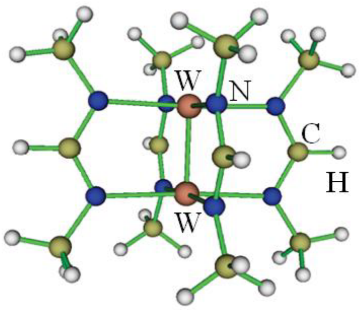

Bond	R_{ij}, Å	*BO*, v.u. Eq. 1 This work	Wiberg *BO*, v.u. Ref.[12ag]	Relative difference, %
W-W	2.175	3.35	3.31	1.2
W-N	2.131	0.48	0.53	9.4

12.5. Bond orders in $Mn_2C_8Me_6$.

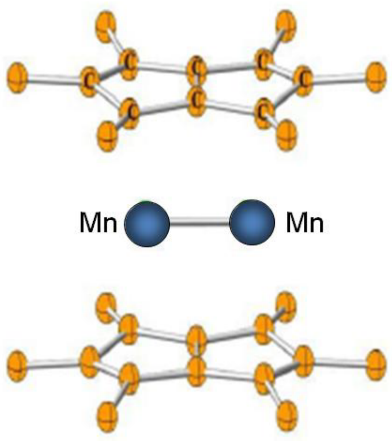

Bond	R_{ij}, Å	*BO*, v.u. Eq. 1 This work	*BO*, v.u. Ref.[49d]		Relative difference, %	
			DI	Wiberg	This work-Wiberg	Wiberg-DI
Mn1-Mn2	2.202	0.94	1.11	1.23	23.6	9.8
Mn1-C1	2.053	0.47	0.50	0.64	26.6	21.9
Mn1-C2	2.060	0.46	0.42	0.58	20.7	27.6
Mn1-C3	2.053	0.47	0.50	0.64	26.6	21.9
Mn1-C4	2.161	0.33	0.29	0.40	12.1	21.2
Mn1-C5	2.161	0.33	0.29	0.40	12.1	21.2

12.6. Bond orders in the planar uranium oxides.

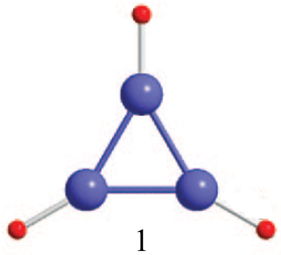

1

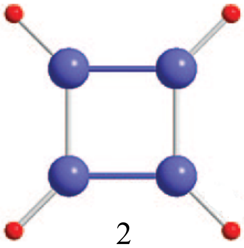

2

Balance of the valence electrons:
The sum of valence electrons for U atom should be equal to 8:
6 (uranium electrons) + 2 (oxygen electrons)
Each U is bonded to oxygen atom and two other U atoms. The sum of bond orders for the three bonds should be equal to 8 v.u.

N	Compound	Bond	R_{ij}, Å	*BO*, v.u. Eq. 1 This work	Discrepancy in the number of valence electrons, v.u.	Wiberg Bond Index, v.u. Ref. [93]	Discrepancy in the number of valence electrons, v.u.
1	cyclo-U_3O_3	U-U<br>U-O	2.539<br>1.833	2.898<br>1.888		2.735<br>2.028	
		BVS		7.684	0.316	7.498	0.502
2	cyclo-U_4O_4	U-U<br>U-O	2.539<br>1.830	2.898<br>1.902		2.478<br>2.059	
		BVS		7.698	0.302	7.015	0.985

[1] G. B. Kaufman, *Inorganic chemistry: principles of structure and reactivity*, ACS Publications, 1993.
[2] aE. Espinosa, E. Molins, C. Lecomte, *Chem. Phys. Lett.* 1998, *285*, 170-173; bE. Espinosa, I. Alkorta, J. Elguero, E. Molins, *J. Chem. Phys.* 2002, *117*, 5529-5542.
[3] R. F. W. Bader, *Atoms in Molecules: A Quantum Theory*, Clarendon Press, 1994.
[4] F. D. Hardcastle, I. E. Wachs, *J. Raman Spectrosc.* 1990, *21*, 683-691.
[5] aC. V. J. Skipper, T. K. A. Hoang, D. M. Antonelli, N. Kaltsoyannis, *Chem. Eur. J.* 2012, *18*, 1750-1760; bM. C. F. Wander, B. R. Bickmore, L. Lind, C. Andros, J. Hunt, H. Checketts, T. Goodell, *Am. Mineral.* 2015, *100*, 160-171; cD. J. Wolstenholme, C. F. Matta, T. S. Cameron, *J. Phys. Chem. A* 2007, *111*, 8803-8813; dJ. Oláh, F. Blockhuys, T. Veszprémi, C. Van Alsenoy, *Eur. J. Inorg. Chem.* 2006, *2006*, 69-77; eP. L. A. Popelier, *J. Phys. Chem. A* 1998, *102*, 1873-1878; fE. A. Zhurova, C. F. Matta, N. Wu, V. V. Zhurov, A. A. Pinkerton, *J. Am. Chem. Soc.* 2006, *128*, 8849-8861; gA. S. Goloveshkin, I. S. Bushmarinov, A. A. Korlyukov, M. I. Buzin, V. I. Zaikovskii, N. D. Lenenko, A. S. Golub, *Langmuir* 2015, *31*, 8953-8960; hC. V. J. Skipper, D. M. Antonelli, N. Kaltsoyannis, *J. Phys. Chem. C* 2012, *116*, 19134-19144; iI. E. Golub, O. A. Filippov, E. I. Gutsul, N. V. Belkova, L. M. Epstein, A. Rossin, M. Peruzzini, E. S. Shubina, *Inorg. Chem.* 2012, *51*, 6486-6497; jD. M. Camaioni, B. Ginovska-Pangovska, G. K. Schenter, S. M. Kathmann, T. Autrey, *J. Phys. Chem. A* 2012, *116*, 7228-7237; kP. R. Varadwaj, A. Varadwaj, G. H. Peslherbe, H. M. Marques, *J. Phys. Chem. A* 2011, *115*, 13180-13190; lL. Rincón, R. Almeida, *J. Phys. Chem. A* 1998, *102*, 9244-9254; mC. F. Matta, N. Castillo, R. J. Boyd, *J. Phys. Chem. A* 2005, *109*, 3669-3681; nN. Mandal, S. M. Pratik, A. Datta, *J. Phys. Chem. B* 2017, *121*, 825-834; oP. Panini, D. Chopra, *Cryst. Growth Des.* 2014, *14*, 3155-3168.
[6] aE. Espinosa, I. Alkorta, I. Mata, E. Molins, *J. Phys. Chem. A* 2005, *109*, 6532-6539; bL. Zhang, F. Ying, W. Wu, P. C. Hiberty, S. Shaik, *Chem. Eur. J.* 2009, *15*, 2979-2989; cS. J. Grabowski, *Crystals* 2015, *6*, 3; dN. J. M. Amezaga, S. C. Pamies, N. M. Peruchena, G. L. Sosa, *J. Phys. Chem. A* 2010, *114*, 552-562; eN. N. Chipanina, N. F. Lazareva, T. N. Aksamentova, E. P. Doronina, B. A. Shainyan, *J. Phys. Org. Chem.* 2014, *27*, 892-901; fR. Bianchi, A. Forni, T. Pilati, *Chem. Eur. J.* 2003, *9*, 1631-1638.
[7] aM. V. Vener, A. V. Manaev, A. N. Egorova, V. G. Tsirelson, *J. Phys. Chem. A* 2007, *111*, 1155-1162; bR. N. Singh, P. Rawat, *J. Mol. Struct.* 2013, *1054–1055*, 65-75; cM. M. Montero-Campillo, A. M. Lamsabhi, O. Mó, M. Yáñez, *J. Mol. Model.* 2013, *19*, 2759-2766; dA. T. Dubis, P. Stasiewicz, K. Pogorzelec-Glaser, A. Łapiński, *J. Phys. Org. Chem.* 2015, *28*, 652-662; eG. Villani, *PCCP* 2013, *15*, 19242-19252; fP. Li, W. Niu, T. Gao, H. Wang, *ChemPhysChem* 2014, *15*, 3078-3088; gS. B. Novaković, G. A. Bogdanović, B. Fraisse, N. E. Ghermani, N. Bouhmaida, A. Spasojević-de Biré, *J. Phys. Chem. A* 2007, *111*, 13492-13505; hP. Munshi, T. N. Guru Row, *Acta Cryst. B.* 2006, *62*, 612-626; iX. Xu, PhD thesis, Ecole Centrale Paris 2015; jM. C. Daza, J. Dobado, J. M. Molina, J. L. Villaveces, *PCCP* 2000, *2*, 4089-4094; kL. Guillaumes, P. Salvador, S. Simon, *J. Phys. Chem. A* 2014, *118*, 1142-1149; lM. Palusiak, S. Simon, M. Solà, *J. Org. Chem.* 2006, *71*, 5241-5248; mJ. Poater, X. Fradera, M. Sola, M. Duran, S. Simon, *Chem. Phys. Lett.* 2003, *369*, 248-255; nM. Palusiak, S. Simon, M. Sola, *Chem. Phys.* 2007, *342*, 43-54; oH. Raissi, A. F. Jalbout, M. A. Nasseria, M. Yoosefian, H. Ghiassi, A. J. Hameed, *Int. J. Quantum Chem* 2008, *108*, 1444-1451; pJ. L. Perez-Lustres, M. Bräuer, M. Mosquera, T. Clark, *PCCP* 2001, *3*, 3569-3579; qI. Majerz, *Mol. Phys.* 2007, *105*, 2305-2314; rH. Karabıyık, R. Sevinçek, H. Petek, M. Aygün, *J. Mol. Model.* 2011, *17*, 1295-1309; sP. R. Mallinson, G. T. Smith, C. C. Wilson, E. Grech, K. Wozniak, *J. Am. Chem. Soc.* 2003, *125*, 4259-4270; tA. Nash, J. Saßmannshausen, L. Bozec, H. L. Birch, N. H. de Leeuw, *J. Biomol. Struct. Dyn.* 2017, *35*, 1127-1137; uG. Saleh, PhD thesis, Milano, Italy 2014; vP. Roversi, F. Merati, R. Destro, M. Barzaghi, *Can. J. Chem.* 1996, *74*, 1145-1161; wL. Bertini, V. Barbieri, P. Fantucci, L. D. Gioia, G. Zampella, *Dalton Trans.* 2011, *40*, 7704-7712; xS. J. Grabowski, W. A. Sokalski, J. Leszczynski, *J. Phys. Chem. A* 2006, *110*, 4772-4779; yY. H. Mariam, R. N. Musin, *J. Phys. Chem. A* 2008, *112*, 134-145; zM. Lukomska, A. J. Rybarczyk-Pirek, M. Jablonski, M. Palusiak, *PCCP* 2015, *17*, 16375-16387; aaM. Vener, E. Levina, A. Astakhov, V. Tsirelson, *Chem. Phys. Lett.* 2015, *638*, 233-236; abV. V. Zhurov, A. A. Pinkerton, *Z. Anorg. Allg. Chem.* 2013, *639*, 1969-1978; acA. Nowroozi, E. Nakhaei, E. Masumian, *Struct. Chem.* 2014, *25*, 1415-1422; adA. Nowroozi, H. Raissi, H. Hajiabadi, P. M. Jahani, *Int. J. Quantum Chem* 2011, *111*, 3040-3047; aeK. Sutter, G. A. Aucar, J. Autschbach, *Chem. Eur. J.* 2015, *21*, 18138-18155.
[8] aV. G. Tsirelson, E. V. Bartashevich, A. I. Stash, V. A. Potemkin, *Acta Crystallographica Section B: Structural Science* 2007, *63*, 142-150; bD. Devarajan, PhD thesis, Philipps-Universität Marburg 2011; cJ. S. Al-Otaibi, *SpringerPlus* 2015, *4*, 586; dM. Kubicki, T. Borowiak, G. Dutkiewicz, M. Souhassou, C. Jelsch, C. Lecomte, *J. Phys. Chem. B* 2002, *106*, 3706-3714; eC. Merkens, F. Pan, U. Englert, *CrystEngComm* 2013, *15*, 8153-8158; fV. Legrand, S. Pillet, M. Souhassou, N. Lugan, C. Lecomte, *J. Am. Chem. Soc.* 2006, *128*, 13921-13931; gY.-R. Guo, Q. Wu, S. O. Odoh, G. Schreckenbach, Q.-J. Pan, *Inorg. Chem.* 2013, *52*, 9143-9152; hP. V. Bharatam, P. Iqbal, *J. Comput. Chem.* 2006, *27*, 334-343; iG. Rajalakshmi, V. R. Hathwar, P. Kumaradhas, *Acta Cryst. B.* 2014, *70*, 331-341; jJ. Dobado, H. Martínez-García, J. M. Molina, M. R. Sundberg, *J. Am. Chem. Soc.* 1998, *120*, 8461-8471; kJ. M. V. Franco, PhD thesis, Friedrich-Wilhelms-Universität Bonn, Germany 2015; lI. Mata, E. Molins, I.

Alkorta, E. Espinosa, *J. Phys. Chem. A* 2007, *111*, 6425-6433; mI. Rozas, *PCCP* 2007, *9*, 2782-2790; nK. K. Pandey, *New J. Chem.* 2016, *40*, 7831-7839; oM. D. Esrafili, F. Mohammadian-Sabet, *Can. J. Chem.* 2014, *92*, 1151-1156; pN. N. Chipanina, N. F. Lazareva, T. N. Aksamentova, A. Y. Nikonov, B. A. Shainyan, *Organometallics* 2014, *33*, 2641-2652; qM. Liu, Q. Li, S. Scheiner, *PCCP* 2017, *19*, 5550-5559; rS. J. Grabowski, *ChemPhysChem* 2014, *15*, 876-884; sY. V. Nelyubina, A. A. Korlyukov, K. A. Lyssenko, *ChemPhysChem* 2015, *16*, 676-681; tI. B. Dorofeeva, O. G. Tarakanov, *Russ. J. Struc. Chem.* 1984, *25*, 23-28; uV. Tognetti, L. Joubert, *J. Phys. Chem. A* 2011, *115*, 5505-5515; vM. Szaleniec, A. Drzewiecka-Matuszek, M. Witko, P. Hejduk, *J. Mol. Model.* 2013, *19*, 4487-4501.

[9] aP. Macchi, A. Sironi, in *The Quantum Theory of Atoms in Molecules*, Wiley-VCH Verlag GmbH & Co. KGaA, 2007, pp. 343-374; bX. Fradera, M. A. Austen, R. F. Bader, *J. Phys. Chem. A* 1999, *103*, 304-314; cL. F. Veiros, M. J. Calhorda, *Dalton Trans.* 2011, *40*, 11138-11146; dL. H. Dos Santos, A. Lanza, A. M. Barton, J. Brambleby, W. J. Blackmore, P. A. Goddard, F. Xiao, R. C. Williams, T. Lancaster, F. L. Pratt, *J. Am. Chem. Soc.* 2016, *138*, 2280-2291; eC. G. Gianopoulos, B. Zarychta, S. Cenedese, V. V. Zhurov, A. A. Pinkerton, *J. Phys. Chem. A* 2016, *120*, 4059-4070; fJ. Overgaard, H. F. Clausen, J. A. Platts, B. B. Iversen, *J. Am. Chem. Soc.* 2008, *130*, 3834-3843; gW. Scherer, P. Sirsch, D. Shorokhov, M. Tafipolsky, G. S. McGrady, E. Gullo, *Chem. Eur. J.* 2003, *9*, 6057-6070; hI. Vidal, S. Melchor, I. Alkorta, J. Elguero, M. R. Sundberg, J. A. Dobado, *Organometallics* 2006, *25*, 5638-5647; iJ. Hey, D. Leusser, D. Kratzert, H. Fliegl, J. M. Dieterich, R. A. Mata, D. Stalke, *PCCP* 2013, *15*, 20600-20610; jU. Flierler, M. Burzler, D. Leusser, J. Henn, H. Ott, H. Braunschweig, D. Stalke, *Angew. Chem. Int. Ed.* 2008, *47*, 4321-4325; kJ. M. Molina, J. A. Dobado, S. Melchor, *J. Mol. Struct. THEOCHEM* 2002, *589*, 337-347; lJ. M. Cole, PhD thesis, Durham University, England 1997; mY. A. Abramov, L. Brammer, W. T. Klooster, R. M. Bullock, *Inorg. Chem.* 1998, *37*, 6317-6328; nI. Alkorta, I. Rozas, J. Elguero, *Struct. Chem.* 1998, *9*, 243-247; oJ. A. Platts, S. T. Howard, *J. Chem. Soc., Perkin Trans.* 1997, 2241-2248; pJ. Poater, M. Solà, M. Duran, X. Fradera, *J. Phys. Chem. A* 2001, *105*, 2052-2063; qD. S. Yufit, J. A. Howard, M. G. Davidson, *J. Chem. Soc., Perkin Trans. 2* 2000, 249-253; rT. Q. Teodoro, R. L. A. Haiduke, *RSC Advances* 2014, *4*, 39853-39859; sN. Ramanathan, K. Sundararajan, K. Vidya, E. D. Jemmis, *Spectrochim. Acta, Part A* 2016, *157*, 69-78; tM. Zhang, Z. Liang, J. Ling, X. Ni, Z. Shen, *Dalton Trans.* 2015, *44*, 11182-11190.

[10] aG. S. McGrady, P. Sirsch, N. P. Chatterton, A. Ostermann, C. Gatti, S. Altmannshofer, V. Herz, G. Eickerling, W. Scherer, *Inorg. Chem.* 2009, *48*, 1588-1598; bC. Hauf, J. E. Barquera-Lozada, P. Meixner, G. Eickerling, S. Altmannshofer, D. Stalke, T. Zell, D. Schmidt, U. Radius, W. Scherer, *Z. Anorg. Allg. Chem.* 2013, *639*, 1996-2004; cI. Alkorta, I. Rozas, J. Elguero, *Int. J. Quantum Chem* 2002, *86*, 122-129; dW. Scherer, G. Eickerling, M. Tafipolsky, G. S. McGrady, P. Sirsch, N. P. Chatterton, *Chem. Commun.* 2006, 2986-2988; eA. Jana, D. Leusser, I. Objartel, H. W. Roesky, D. Stalke, *Dalton Trans.* 2011, *40*, 5458-5463; fS. Grabowsky, PhD thesis, Freie Universität Berlin 2010; gM. Dakkouri, V. Novikov, L. Vilkov, *J. Mol. Struct.* 2010, *978*, 234-245; hK. K. Pandey, C. Jones, *Organometallics* 2013, *32*, 3395-3403; iI. Corral, O. Mó, M. Yáñez, *New J. Chem.* 2003, *27*, 1657-1664; jM. Lein, J. A. Harrison, A. J. Nielson, *Dalton Trans.* 2011, *40*, 10731-10741; kW. Scherer, P. Meixner, J. E. Barquera-Lozada, C. Hauf, A. Obenhuber, A. Brück, D. J. Wolstenholme, K. Ruhland, D. Leusser, D. Stalke, *Angew. Chem. Int. Ed.* 2013, *52*, 6092-6096; lS. K. Ignatov, N. H. Rees, S. R. Dubberley, A. G. Razuvaev, P. Mountford, G. I. Nikonov, *Chem. Commun.* 2004, 952-953; mX. Ning, J. Wang, H. Wei, *J. Phys. Chem. A* 2016, *120*, 4167-4178; nT. Stahl, P. Hrobárik, C. D. F. Königs, Y. Ohki, K. Tatsumi, S. Kemper, M. Kaupp, H. F. Klare, M. Oestreich, *Chem. Sci.* 2015, *6*, 4324-4334; oM. I. Arz, G. Schnakenburg, A. Meyer, O. Schiemann, A. C. Filippou, *Chem. Sci.* 2016, *7*, 4973-4979; pK. Petrov, PhD thesis, Budapest University of Technology and Economics 2012; qM. Hagemann, A. Mix, R. J. Berger, T. Pape, N. W. Mitzel, *Inorg. Chem.* 2008, *47*, 10554-10564; rF. P. Arnold, *J. Organomet. Chem.* 2001, *617*, 647-655; sR. J. Gillespie, P. L. A. Popelier, *Chemical Bonding and Molecular Geometry: From Lewis to Electron Densities*, Oxford University Press, 2001; tT. Szilvási, PhD thesis, Budapest University of Technology and Economics 2015; uJ. Xu, Y.-H. Ding, *Aust. J. Chem.* 2014, *67*, 740-748; vT. Fukuda, H. Hashimoto, S. Sakaki, H. Tobita, *Angew. Chem.* 2016, *128*, 196-200; wI. Corral, O. Mó, M. Yáñez, *Int. J. Mass spectrom.* 2003, *227*, 401-412; xV. Montiel-Palma, O. Piechaczyk, A. Picot, A. Auffrant, L. Vendier, P. Le Floch, S. Sabo-Etienne, *Inorg. Chem.* 2008, *47*, 8601-8603; yS. F. Vyboishchikov, G. I. Nikonov, *Organometallics* 2007, *26*, 4160-4169; zW. Scherer, P. Meixner, K. Batke, J. E. Barquera-Lozada, K. Ruhland, A. Fischer, G. Eickerling, K. Eichele, *Angew. Chem. Int. Ed.* 2016, *55*, 11673-11677; aaM. Jabłoński, *Comput. Theor. Chem.* 2016, *1096*, 54-65; abQ.-Z. Li, H.-Y. Zhuo, H.-B. Li, Z.-B. Liu, W.-Z. Li, J.-B. Cheng, *J. Phys. Chem. A* 2014, *119*, 2217-2224; acD. J. Wolstenholme, P. D. Prince, G. S. McGrady, M. J. Landry, J. W. Steed, *Inorg. Chem.* 2011, *50*, 11222-11227; adM. D. Esrafili, F. Mohammadian-Sabet, *J. Mol. Model.* 2015, *21*, 60; aeA. Y. Khalimon, Z. H. Lin, R. Simionescu, S. F. Vyboishchikov, G. I. Nikonov, *Angew. Chem. Int. Ed.* 2007, *46*, 4530-4533; afY. Horbatenko, PhD thesis, Girona University 2013.

[11] aB. Bankiewicz, M. Palusiak, *Comput. Theor. Chem.* 2011, *966*, 113-119; bP. Gómez, O. Gálvez, R. Escribano, *PCCP* 2009, *11*, 9710-9719; cM. Jabłoński, M. Palusiak, *J. Phys. Chem. A* 2012, *116*, 2322-2332; dM. Domagała, P. Matczak, M. Palusiak, *Comput. Theor. Chem.* 2012, *998*, 26-33; eR. Flaig, T. Koritsanszky, B. Dittrich, A. Wagner, P. Luger, *J. Am. Chem. Soc.* 2002, *124*, 3407-3417; fJ. Saßmannshausen, *Dalton Trans.* 2014, *43*, 11195-11201; gP. Lipkowski, S. J. Grabowski, J. Leszczynski, *J. Phys. Chem. A* 2006, *110*, 10296-10302; hX. An, B. Jing, Q. Li, *J. Phys. Chem. A* 2010, *114*, 6438-6443; iJ. R. Mora, J. Lezama, E. Márquez, L. Escalante, T. Córdova, G. Chuchani, *J. Phys. Org. Chem.* 2011, *24*, 229-240; jW. Seifert, S. Schwetlick, J. Reinhold, E. Butter, *J. Cryst. Growth* 1984, *66*, 333-337; kY. G. Wang, C. Matta, N. H. Werstiuk, *J. Comput. Chem.* 2003, *24*, 1720-1729; lB. Bankiewicz, P. Matczak, M. Palusiak, *J. Phys. Chem. A* 2012, *116*, 452-459; mE. Matito, J. Poater, M. Solá, in *The Quantum Theory of Atoms in Molecules*, Wiley-VCH Verlag GmbH & Co. KGaA, 2007, pp. 399-423; nM. Vatanparast, *Comput. Theor. Chem.* 2014, *1048*, 77-83; oD. Dey, S. Bhandary, A. Sirohiwal, V. R. Hathwar, D. Chopra, *Chem. Commun.* 2016, *52*, 7225-7228; pY. Xia, A. S. Dudnik, V. Gevorgyan, Y. Li, *J. Am. Chem. Soc.* 2008, *130*, 6940-6941; qL. Koskinen, S. Jääskeläinen, P. Hirva, M. Haukka, *Solid State Sciences* 2014, *35*, 81-87; rG. V. Baryshnikov, S. V. Bondarchuk, B. F. Minaev, L. V. Kovtun, A. M. Malachov, *Bull. Cherkasy Univ., Chem. Sci.* 2013, 11-19.

[12] aG. La Penna, S. Furlan, M. Solà, *Theor. Chem. Acc.* 2011, *130*, 27; bC. L. Firme, *J. Brazilian Chem. Soc.* 2012, *23*, 513-521; cT. Papp, L. Kollár, T. Kégl, *J. Quantum Chem.* 2014, *2014*; dP. Macchi, A. Sironi, *Coord. Chem. Rev.* 2003, *238*, 383-412; eP. R. Varadwaj, A. Varadwaj, B.-Y. Jin, *PCCP* 2014, *16*, 17238-17252; fF. Cortés-Guzmán, R. F. Bader, *Coord. Chem. Rev.* 2005, *249*, 633-662; gI. Pápai, J. Mascetti, R. Fournier, *J. Phys. Chem. A* 1997, *101*, 4465-4471; hL. J. Farrugia, H. M. Senn, *J. Phys. Chem. A* 2010, *114*, 13418-13433; iL. J. Farrugia, C. Evans, H. M. Senn, M. M. Hänninen, R. Sillanpää, *Organometallics* 2012, *31*, 2559-2570; jC. F. Matta, R. J. Boyd, in *The Quantum Theory of Atoms in Molecules*, Wiley-VCH Verlag GmbH & Co. KGaA, 2007, pp. 1-34; kS. Grabowsky, D. Jayatilaka, R. F. Fink, T. Schirmeister, B. Engels, *Z. Anorg. Allg. Chem.* 2013, *639*, 1905-1921; lN. S. Lambic, R. D. Sommer, E. A. Ison, *J. Am. Chem. Soc* 2016, *138*, 4832-4842; mY. Lee, J. C. Peters, *J. Am. Chem. Soc.* 2011, *133*, 4438-4446; nS. T. Howard, O. Lamarche, *J. Phys. Org. Chem.* 2003, *16*, 133-141; oR. B. Viana, A. R. Guimarães, A. R. de Souza, A. B. F. da Silva, *J. Mol. Model.* 2014, *20*, 2074; pC. Gatti, in *The Quantum Theory of Atoms in Molecules*, Wiley-VCH Verlag GmbH & Co. KGaA, 2007, pp. 163-206; qL. J. Farrugia, P. R. Mallinson, B. Stewart, *Acta Cryst. B.* 2003, *59*, 234-247; rD. J. Duarte, M. S. Miranda, J. C. E. da Silva, J. F. Liebman, *Struct. Chem.* 2016, *27*, 663-670; sJ. Dobado, J. M. Molina, R. Uggla, M. R. Sundberg, *Inorg. Chem.* 2000, *39*, 2831-2836; tS. Pillet, M. Souhassou, C. Mathonière, C. Lecomte, *J. Am. Chem. Soc.* 2004, *126*, 1219-1228; uR. Bianchi, G. Gervasio, D. Marabello, *Inorg. Chem.* 2000, *39*, 2360-2366; vM. A. Alvarez, M. E. García, D. García-Vivó, S. Menéndez, M. A. Ruiz, *Organometallics* 2012, *31*, 7181-7190; wM. A. Alvarez, I. Amor, M. E. García, D. García-Vivó, M. A. Ruiz, J. Suárez, *Organometallics* 2010, *29*, 4384-4395; xM. E. García, D. García-Vivó, S. Melón, M. A. Ruiz, C. Graiff, A. Tiripicchio, *Inorg. Chem.* 2009, *48*, 9282-9293; yM. A. Alvarez, M. E. García, D. García-Vivó, M. E. Martínez, M. A. Ruiz, *Organometallics* 2011, *30*, 2189-2199; zG. F. Caramori, G. Frenking, *Theor. Chem. Acc.* 2008, *120*, 351-361; aaL. J. Farrugia, C. Evans, *J. Phys. Chem. A* 2005, *109*, 8834-8848; abJ. Małecki, T. Groń, H. Duda, *Polyhedron* 2012, *31*, 319-331; acJ. N. Latosińska, J. Seliger, V. Žagar, D. V. Burchardt, *J. Mol. Model.* 2012, *18*, 11-26; adD. Heift, Z. Benkő, H. Grützmacher, A. R. Jupp, J. M. Goicoechea, *Chem. Sci.* 2015, *6*, 4017-4024; aeO. Sizova, A. Y. Sokolov, L. Skripnikov, *Russ. J. Coord. Chem.* 2007, *33*, 800-808; afN. Deora, PhD thesis, Virginia Tech 2010; agG. M. Chiarella, C. Feng, C. A. Murillo, Q. Zhao, *Inorg. Chem.* 2016, *55*, 9471-9481; ahK. K. Pandey, A. Lledós, *J. Organomet. Chem.* 2010, *695*, 206-214; aiZ. S. Marković, J. M. Dimitrić-Marković, D. Milenković, N. Filipović, *Monatsh. Chem.* 2011, *142*, 145-152; ajV. Franco, J. Manuel, G. Schnakenburg, T. Sasamori, A. Espinosa Ferao, R. Streubel, *Chem. Eur. J.* 2015, *21*, 9650-9655; akF. Teixeira, R. Mosquera, A. Melo, C. Freire, M. N. l. DS Cordeiro, *Inorg. Chem.* 2016, *55*, 3653-3662; alH. Raissi, A. Jalbout, M. Nasseria, M. Yoosefian, H. Ghiassi, A. Hameed, *Int. J. Quantum Chem* 2008, *108*, 1444-1451; amS. P. Thomas, M. S. Pavan, T. G. Row, *Chem. Commun.* 2014, *50*, 49-51.

[13] aD. Bykov, PhD thesis, Bonn University 2013; bV. Barbieri, PhD thesis, Milan University 2012; cN. S. Emel'yanova, N. y. Y. Shmatko, N. A. Sanina, S. M. Aldoshin, *Comput. Theor. Chem.* 2015, *1060*, 1-9; dC.-R. Lee, I.-J. Hsu, H.-T. Chen, G.-H. Lee, Y. Wang, *C.R. Chim.* 2012, *15*, 237-249; eV. Shlyapochnikov, M. Tafipolsky, I. Tokmakov, E. Baskir, O. Anikin, Y. A. Strelenko, O. Luk'yanov, V. Tartakovsky, *J. Mol. Struct.* 2001, *559*, 147-166; fS. Berski, A. J. Gordon, *Chem. Phys. Lett.* 2012, *525*, 24-31; gS. Berski, A. J. Gordon, *J. Chem. Phys.* 2011, *135*, 094303; hS. Berski, A. J. Gordon, Z. Latajka, *J. Chem. Phys.* 2013, *138*, 134313; iS. V. Shishkina, A. I. Slabko, S. Berski, Z. Latajka, O. V. Shishkin, *J. Chem. Phys.* 2013, *139*, 124308; jV. G. Tsirelson, A. V. Shishkina, A. I. Stash, S. Parsons, *Acta Cryst. B.* 2009, *65*, 647-658; kA. J. Rybarczyk-Pirek, M. Łukomska-Rogala, S. Wojtulewski, M. Palusiak, *Cryst. Growth Des.* 2015, *15*, 5802-5815; lF. Hill, G. Gibbs, M. Boisen Jr, *Phys. Chem. Miner.*

1997, *24*, 582-596; mC. Trujillo, G. Sánchez-Sanz, I. Alkorta, J. Elguero, *New J. Chem.* 2015, *39*, 6791-6802; nD. Chesnut, *J. Am. Chem. Soc.* 1998, *120*, 10504-10510; oM. A. Samsonov, PhD thesis, Nizhny Novgorod State Technical University 2017; pL. K. Harper, A. L. Shoaf, C. A. Bayse, *ChemPhysChem* 2015, *16*, 3886-3892; qÁ. Vega-Vega, A. Largo, P. Redondo, C. Barrientos, *ACS Earth Space Chem.* 2017, *1*, 158-167; rE. A. Zhurova, A. I. Stash, V. G. Tsirelson, V. V. Zhurov, E. V. Bartashevich, V. A. Potemkin, A. A. Pinkerton, *J. Am. Chem. Soc.* 2006, *128*, 14728-14734; sA. Y. Rogachev, P. Burger, *PCCP* 2012, *14*, 1985-2000; tM. Sajid, A. Stute, A. J. P. Cardenas, B. J. Culotta, J. A. Hepperle, T. H. Warren, B. Schirmer, S. Grimme, A. Studer, C. G. Daniliuc, *J. Am. Chem. Soc.* 2012, *134*, 10156-10168; uM. J. Chalkley, J. C. Peters, *Angew. Chem.* 2016, *128*, 12174-12177; vI. Nieto-López, L. Hernández-García, J. Bonilla-Cruz, M. Sanchez, *J. Mol. Model.* 2014, *20*, 2149; wB. Wang, Y.-M. Lee, W.-Y. Tcho, S. Tussupbayev, S.-T. Kim, Y. Kim, M. S. Seo, K.-B. Cho, Y. Dede, B. C. Keegan, *Nat. Commun.* 2017, *8*, 14839; xM. Nahali, F. Gobal, *Mol. Phys.* 2011, *109*, 229-237; yM. Sander, A. Raj, O. Inderwildi, M. Kraft, S. Kureti, H. Bockhorn, *Carbon* 2009, *47*, 866-875; zR. Salzmann, M. Wojdelski, M. McMahon, R. H. Havlin, E. Oldfield, *J. Am. Chem. Soc.* 1998, *120*, 1349-1356; aaG. F. Caramori, A. G. Kunitz, D. F. Coimbra, L. C. Garcia, D. E. Fonseca, *J. Brazilian Chem. Soc.* 2013, *24*, 1487-1496; abG. Wu, J. Zhu, X. Mo, R. Wang, V. Terskikh, *J. Am. Chem. Soc.* 2010, *132*, 5143-5155; acM. L. Kuznetsov, V. Y. Kukushkin, *Dalton Trans.* 2017, *46*, 786-802; adO. Sizova, N. Ivanova, O. Lyubimova, V. Sizov, *Russ. J. Coord. Chem.* 2007, *33*, 523-529.

[14] aV. G. Avakyan, V. F. Sidorkin, E. F. Belogolova, S. L. Guselnikov, L. E. Gusel'nikov, *Organometallics* 2006, *25*, 6007-6013; bM. Prencipe, M. Tribaudino, F. Nestola, *Phys. Chem. Miner.* 2003, *30*, 606-614; cR. Bianchi, A. Forni, F. Cámara, R. Oberti, H. Ohashi, *Phys. Chem. Miner.* 2007, *34*, 519-527; dR. Bianchi, A. Forni, R. Oberti, *Phys. Chem. Miner.* 2005, *32*, 638-645; eR. Müller, PhD thesis, Technischen Universität Berlin 2014; fN. W. Mitzel, K. Vojinović, R. Fröhlich, T. Foerster, H. E. Robertson, K. B. Borisenko, D. W. Rankin, *J. Am. Chem. Soc.* 2005, *127*, 13705-13713; gQ. Wei, Q. Li, J. Cheng, W. Li, H.-B. Li, *RSC Advances* 2016, *6*, 79245-79253; hS. U. Ahmad, T. Szilvási, E. Irran, S. Inoue, *J. Am. Chem. Soc.* 2015, *137*, 5828-5836; iN. Kocher, PhD thesis, Bayerischen Julius-Maximilians-Universität Würzburg 2003; jV. F. Sidorkin, E. P. Doronina, *Organometallics* 2009, *28*, 5305-5315; kR. J. Gillespie, S. A. Johnson, *Inorg. Chem.* 1997, *36*, 3031-3039; lM. Nechaev, T. Aksamentova, M. Voronkov, N. Chipanina, O. Trofimova, Y. I. Bolgova, V. Turchaninov, *Russ. J. Gen. Chem.* 2009, *79*, 1086-1089; mN. N. Chipanina, N. F. Lazareva, T. N. Aksamentova, A. Y. Nikonov, B. A. Shainyan, *Organometallics* 2014, *33*, 2641-2652; nN. N. Chipanina, N. F. Lazareva, L. P. Oznobikhina, I. M. Lazarev, B. A. Shainyan, *Comput. Theor. Chem.* 2015, *1070*, 162-173; oJ. Dillen, *J. Phys. Chem. A* 2004, *108*, 4971-4977; pM. K. Hazrati, N. L. Hadipour, *Phys. Lett. A* 2016, *380*, 937-941; qY.-t. Liu, X. Wang, X.-y. Liu, X.-p. Li, Y.-q. Ji, *Chem. Res. Chin. Univ.* 2013, *29*, 351-354; rJ. D. Epping, S. Yao, M. Karni, Y. Apeloig, M. Driess, *J. Am. Chem. Soc.* 2010, *132*, 5443-5455; sD. Cortés-Arriagada, A. Toro-Labbé, *RSC Advances* 2016, *6*, 28500-28511; tC. M. Marchand, U. Pidun, G. Frenking, H. Grützmacher, *J. Am. Chem. Soc.* 1997, *119*, 11078-11085; uM. Liu, Q. Li, J. Cheng, W. Li, H.-B. Li, *J. Chem. Phys.* 2016, *145*, 224310; vJ.-F. Boily, *J. Phys. Chem. A* 2002, *106*, 4718-4724; wI. Alkorta, I. Rozas, J. Elguero, *J. Phys. Chem. A* 2001, *105*, 743-749; xR. Rodriguez, D. Gau, T. Troadec, N. Saffon-Merceron, V. Branchadell, A. Baceiredo, T. Kato, *Angew. Chem.* 2013, *125*, 9150-9153; yY. Xiong, S. Yao, R. Müller, M. Kaupp, M. Driess, *Nat. Chem.* 2010, *2*, 577-580; zS. S. Sen, *Angew. Chem. Int. Ed.* 2014, *53*, 8820-8822; aaR. Rodriguez, D. Gau, J. Saouli, A. Baceiredo, N. Saffon-Merceron, V. Branchadell, T. Kato, *Angew. Chem.* 2017, *129*, 3993-3997; abM. E. Alikhani, *J. Chem. Soc., Faraday Trans.* 1997, *93*, 3305-3308; acE. P. Doronina, V. F. Sidorkin, N. F. Lazareva, *J. Phys. Chem. A* 2015, *119*, 3663-3673; adP. Suman, S. Janardan, M. Y. Lone, P. Jha, K. Vijayakrishna, A. Sivaramakrishna, *Polyhedron* 2015, *99*, 266-274; aeI. Beletskii, A. Grebenyuk, V. Klimenko, *Theor. Exp. Chem.* 1995, *30*, 187-190; afY. I. Gorlov, V. Zaets, M. Terets, A. Chuiko, *Theor. Exp. Chem.* 1991, *27*, 17-19; agY. I. Gorlov, V. Zaets, A. Chuiko, *Theor. Exp. Chem.* 1989, *24*, 516-526.

[15] aM. Alonso, M. A. Alvarez, M. E. García, D. García-Vivó, M. A. Ruiz, *Dalton Trans.* 2014, *43*, 16074-16083; bJ. Pérez, E. Pérez, R. Carrascosa, A. Espinosa, L. García, J. L. Serrano, *J. Mol. Struct.* 2010, *968*, 52-58; cJ. M. V. Franco, T. Sasamori, G. Schnakenburg, A. E. Ferao, R. Streubel, *Chem. Commun.* 2015, *51*, 3878-3881; dG. Balázs, J. C. Green, D. M. P. Mingos, *Eur. J. Inorg. Chem.* 2007, *2007*, 2443-2453; eM. Gall, M. Breza, *J. Mol. Struct. THEOCHEM* 2009, *894*, 32-35; fE. Aubert, PhD thesis, Université Henri Poincaré - Nancy I 2003; gN. Y. Acelas, D. López, F. Mondragón, W. Tiznado, E. Flórez, *J. Mol. Model.* 2013, *19*, 2057-2067; hC. F. Matta, A. A. Arabi, T. A. Keith, *J. Phys. Chem. A* 2007, *111*, 8864-8872; iA. Mermer, T. Lis, P. Starynowicz, *RSC Advances* 2015, *5*, 96623-96638; jA. Hernandez-Laguna, C. Sainz-Diaz, Y. Smeyers, J. De Paz, E. Galvez-Ruano, *J. Phys. Chem.* 1994, *98*, 1109-1116; kD. Hou, Z. Yang, S. Pan, *J. Alloys Compd.* 2017, *706*, 589-595; lM. A. Austen, PhD thesis, Dalhousie University 2003; mI. Alkorta, J. Elguero, S. J. Grabowski, *PCCP* 2015, *17*, 3261-3272; nA. A. Restrepo-Cossio, H. Cano, F. Mari, C. A. Gonzalez, *Heteroat. Chem* 1997, *8*, 557-569; oM. Esseffar, A. Luna, O. Mo, M. Yanez, *J. Phys. Chem.* 1994, *98*, 8679-8686; pD. Mandal, K. Sen, A. K. Das, *J.*

Phys. Chem. A 2012, *116*, 8382-8396; qA. T. Carvalho, A. C. O'Donoghue, D. R. Hodgson, S. C. Kamerlin, *Org. Biom. Chem.* 2015, *13*, 5391-5398.

[16] aA. Poater Teixidor, PhD thesis, Universitat de Girona 2006; bC. J. Cramer, W. B. Tolman, K. H. Theopold, A. L. Rheingold, *PNAS* 2003, *100*, 3635-3640; cM. F. Qayyum, R. Sarangi, K. Fujisawa, T. D. P. Stack, K. D. Karlin, K. O. Hodgson, B. Hedman, E. I. Solomon, *J. Am. Chem. Soc.* 2013, *135*, 17417-17431; dC. Du, H. Lin, B. Lin, Z. Ma, T. Hou, J. Tang, Y. Li, *J. Mater. Chem. A* 2015, *3*, 23113-23119; eA. F. Cozzolino, D. Tofan, C. C. Cummins, M. Temprado, T. D. Palluccio, E. V. Rybak-Akimova, S. Majumdar, X. Cai, B. Captain, C. D. Hoff, *J. Am. Chem. Soc.* 2012, *134*, 18249-18252; fC. Rovira, K. Kunc, J. Hutter, P. Ballone, M. Parrinello, *J. Phys. Chem. A* 1997, *101*, 8914-8925; gV. Luana, A. Costales, P. Mori-Sanchez, A. M. Pendás, *J. Phys. Chem. B* 2003, *107*, 4912-4921; hG. V. Gibbs, R. Downs, D. F. Cox, N. L. Ross, M. Boisen, K. M. Rosso, *J. Phys. Chem. A* 2008, *112*, 3693-3699; iG. Gibbs, M. Boisen, K. Rosso, D. Teter, M. Bukowinski, *J. Phys. Chem. B* 2000, *104*, 10534-10542; jG. Gibbs, O. Tamada, M. Boisen, F. Hill, *Am. Mineral.* 1999, *84*, 435-446; kA. Pakiari, K. Eskandari, *J. Mol. Struct. THEOCHEM* 2007, *806*, 1-7; lC. Sharma, A. K. Singh, J. Joy, E. D. Jemmis, S. K. Awasthi, *RSC Advances* 2016, *6*, 91689-91693; mV. R. Hathwar, M. K. Thomsen, M. A. H. Mamakhel, M. Ø. Filsø, J. Overgaard, B. B. Iversen, *J. Phys. Chem. A* 2016, *120*, 7510-7518; nX. Fradera, M. Solà, *J. Comput. Chem.* 2002, *23*, 1347-1356; oY. Yang, *J. Phys. Chem. A* 2012, *116*, 10150-10159; pD. E. Trujillo-González, M. C. Ramírez-Romero, J. I. Rodríguez, E. A. Uribe, *Chem. Phys. Lett.* 2016, *649*, 103-110; qE. A. Zhurova, A. Martin, A. A. Pinkerton, *J. Am. Chem. Soc.* 2002, *124*, 8741-8750; rY. V. Nelyubina, K. A. Lyssenko, *Chem. Eur. J.* 2012, *18*, 12633-12636.

[17] aS. Kundu, C. Mohapatra, P. P. Samuel, J. Kretsch, M. Walawalkar, R. Herbst-Irmer, D. Stalke, S. De, D. Koley, H. W. Roesky, *Chem. Commun.* 2017, *53*, 192-195; bQ. Tang, Q. Li, *Comput. Theor. Chem.* 2014, *1050*, 51-57; cH. Xu, J. Cheng, X. Yang, Z. Liu, X. Bo, Q. Li, *RSC Advances* 2017, *7*, 21713-21720; dM. D. Esrafili, F. Mohammadian-Sabet, *Mol. Phys.* 2016, *114*, 83-91; eM. D. Esrafili, F. Mohammadian-Sabet, *Mol. Phys.* 2016, *114*, 1528-1538; fM. Liu, L. Yang, Q. Li, W. Li, J. Cheng, B. Xiao, X. Yu, *J. Mol. Model.* 2016, *22*, 192; gM. Marín-Luna, I. Alkorta, J. Elguero, *J. Organomet. Chem.* 2015, *794*, 206-215; hM. Marín-Luna, I. Alkorta, J. Elguero, *Theor. Chem. Acc.* 2017, *136*, 41; iS. J. Grabowski, *Crystals* 2017, *7*, 43; jY. Li, H. Zhu, D. M. Andrada, G. Frenking, H. W. Roesky, *Chem. Commun.* 2014, *50*, 4628-4630; kT. Müller, Y. Apeloig, I. Hemme, U. Klingebiel, M. Noltemeyer, *J. Organomet. Chem.* 1995, *494*, 133-142; lM. Novak, L. Dostál, M. Alonso, F. De Proft, A. Růžička, A. Lyčka, R. Jambor, *Chem. Eur. J.* 2014, *20*, 2542-2550; mY. Xiong, S. Yao, S. Inoue, E. Irran, M. Driess, *Angew. Chem. Int. Ed.* 2012, *51*, 10074-10077; nU. Burckhardt, G. L. Casty, T. D. Tilley, T. K. Woo, U. Rothlisberger, *Organometallics* 2000, *19*, 3830-3841; oY. Yang, *J. Phys. Chem. A* 2010, *114*, 13257-13267; pD. Dhara, T. Vijayakanth, M. K. Barman, K. P. K. Naik, N. Chrysochos, C. B. Yildiz, R. Boomishankar, C. Schulzke, V. Chandrasekhar, A. Jana, *Chem. Commun.* 2017, *53*, 8592-8595; qT. Ochiai, D. Franz, S. Inoue, *Chem. Soc. Rev.* 2016, *45*, 6327-6344; rS. Inoue, K. Leszczyńska, *Angew. Chem.* 2012, *124*, 8717-8721; sP. Jutzi, *Chem. Eur. J.* 2014, *20*, 9192-9207; tV. G. Avakyan, E. P. Doronina, S. L. Guselnikov, V. F. Sidorkin, *J. Organomet. Chem.* 2011, *696*, 1969-1974; uM. S. Gordon, M. T. Carroll, J. H. Jensen, L. P. Davis, L. W. Burggraf, R. M. Guidry, *Organometallics* 1991, *10*, 2657-2660; vG. Csonka, P. Hencsei, *J. Organomet. Chem.* 1993, *446*, 99-106; wG. Csonka, P. Hencsei, *J. Mol. Struct. THEOCHEM* 1993, *283*, 251-259; xV. F. Sidorkin, E. F. Belogolova, E. P. Doronina, *PCCP* 2015, *17*, 26225-26237; yV. Romanovs, V. Sidorkin, E. Belogolova, V. Jouikov, *Dalton Trans.* 2017; zG. W. Fester, J. r. Wagler, E. Brendler, U. Böhme, D. Gerlach, E. Kroke, *J. Am. Chem. Soc.* 2009, *131*, 6855-6864.

[18] aJ. Cioslowski, S. T. Mixon, *J. Am. Chem. Soc.* 1991, *113*, 4142-4145; bY. S. Won, Y. S. Kim, T. J. Anderson, L. L. Reitfort, I. Ghiviriga, L. McElwee-White, *J. Am. Chem. Soc.* 2006, *128*, 13781-13788; cC. Cabezas, C. Barrientos, A. Largo, J.-C. Guillemin, J. Alonso, *PCCP* 2016, *18*, 28538-28547; dA. A. Melekhova, A. S. Novikov, N. A. Bokach, M. S. Avdonceva, V. Y. Kukushkin, *Inorg. Chim. Acta* 2016, *450*, 140-145; eZ. Setifi, K. V. Domasevitch, F. Setifi, P. Mach, S. W. Ng, V. Petříček, M. Dušek, *Acta Cryst. C.* 2013, *69*, 1351-1356; fD. Kratzert, PhD thesis, Göttingen 2013; gD. E. Hibbs, C. J. Austin-Woods, J. A. Platts, J. Overgaard, P. Turner, *Chem. Eur. J.* 2003, *9*, 1075-1084; hJ. A. Dobado, R. Uggla, M. R. Sundberg, J. Molina, *Dalton Trans.* 1999, 489-496; iE. Espinosa, E. Molins, C. Lecomte, *Phys. Rev. B.* 1997, *56*, 1820; jL. Álvarez-Rodríguez, J. Brugos, J. A. Cabeza, P. García-Álvarez, E. Pérez-Carreño, D. Polo, *Chem. Commun.* 2017, *53*, 893-896; kX.-H. Li, R.-Z. Zhang, H.-L. Cui, Y.-L. Yong, *Chem. Heterocycl. Compd.* 2015, *51*, 153-158; lC. Bo, J. M. Poblet, M. Bénard, *Chem. Phys. Lett.* 1990, *169*, 89-96; mH. Jansen, F. B. Läng, J. C. Slootweg, A. W. Ehlers, M. Lutz, K. Lammertsma, H. Grützmacher, *Angew. Chem.* 2010, *122*, 5617-5620; nZ. Azizi, M. Ghambarian, M. A. Rezaei, M. Ghashghaee, *Aust. J. Chem.* 2015, *68*, 1438-1445; oP. J. Byrne, PhD thesis, University of St Andrews 2009; pM. Liu, Q. Li, W. Li, J. Cheng, *Struct. Chem.* 2017, *28*, 823-831; qB. Freitag, C. A. Fischer, J. Penafiel, G. Ballmann, H. Elsen, C. Färber, D. F. Piesik, S. Harder, *Dalton Trans.* 2017, *46*, 11192-11200 ; rA. Bauzá, T. J. Mooibroek, A. Frontera, *Chemical Record* 2016, *16*, 473-487; sA.

Ariafard, M. D. Asli, H. Aghabozorg, H. Aghabozorg, M. Monajjemi, *J. Mol. Struct. THEOCHEM* 2003, *636*, 49-56; tJ. M. V. Franco, G. Schnakenburg, A. E. Ferao, R. Streubel, *Dalton Trans.* 2016, *45*, 13951-13956; uG. von Frantzius, A. E. Ferao, R. Streubel, *Chem. Sci.* 2013, *4*, 4309-4322; vD. Mani, E. Arunan, *PCCP* 2013, *15*, 14377-14383; wS. J. Grabowski, *PCCP* 2014, *16*, 1824-1834; xM. D. Esrafili, N. Mohammadirad, M. Solimannejad, *Chem. Phys. Lett.* 2015, *628*, 16-20.

[19] aC. L. Firme, S. E. Galembeck, O. Antunes, P. M. Esteves, *J. Brazilian Chem. Soc.* 2007, *18*, 1397-1404; bK. Albahily, PhD thesis, Ottawa-Carleton Chemistry Institute 2011; cC. Lacaze-Dufaure, T. Mineva, N. Russo, *J. Comput. Chem.* 2001, *22*, 1557-1564; dT. Lu, F. Chen, *J. Phys. Chem. A* 2013, *117*, 3100-3108; eM. Tafipolsky, W. Scherer, K. Öfele, G. Artus, B. Pedersen, W. A. Herrmann, G. S. McGrady, *J. Am. Chem. Soc.* 2002, *124*, 5865-5880; fL. J. Farrugia, C. Evans, D. Lentz, M. Roemer, *J. Am. Chem. Soc.* 2008, *131*, 1251-1268; gA. I. Baranov, M. Kohout, *J. Comput. Chem.* 2011, *32*, 2064-2076; hV. César, L. C. Misal Castro, T. Dombray, J.-B. Sortais, C. Darcel, S. p. Labat, K. Miqueu, J.-M. Sotiropoulos, R. m. Brousses, N. Lugan, *Organometallics* 2013, *32*, 4643-4655; iS. Scheins, M. Messerschmidt, M. Gembicky, M. Pitak, A. Volkov, P. Coppens, B. G. Harvey, G. C. Turpin, A. M. Arif, R. D. Ernst, *J. Am. Chem. Soc.* 2009, *131*, 6154-6160; jS. K. Brayshaw, E. L. Sceats, J. C. Green, A. S. Weller, *PNAS* 2007, *104*, 6921-6926; kC. Gatti, *Z. Kristallogr.* 2005, *220*, 399-457; lH. Nuss, N. Claiser, S. Pillet, N. Lugan, E. Despagnet-Ayoub, M. Etienne, C. Lecomte, *Dalton Trans.* 2012, *41*, 6598-6601; mR. Destro, R. Soave, M. Barzaghi, L. Lo Presti, *Chem. Eur. J.* 2005, *11*, 4621-4634; nC. L. Firme, O. Antunes, P. M. Esteves, *J. Brazilian Chem. Soc.* 2009, *20*, 543-548; oC. L. Firme, O. Antunes, P. M. Esteves, *Chem. Phys. Lett.* 2009, *468*, 129-133; pP. Popelier, G. Logothetis, *J. Organomet. Chem.* 1998, *555*, 101-111; qJ. Henn, D. Leusser, D. Stalke, *J. Comput. Chem.* 2007, *28*, 2317-2324; rF. Feixas, E. Matito, J. Poater, M. Sola, *J. Phys. Chem. A* 2011, *115*, 13104-13113; sK. Remya, C. H. Suresh, *PCCP* 2015, *17*, 27035-27044.

[20] aM. Ray, Y. Nakao, H. Sato, H. Sakaba, S. Sakaki, *Organometallics* 2008, *28*, 65-73; bW. Scherer, P. Sirsch, M. Grosche, M. Spiegler, S. A. Mason, M. G. Gardiner, *Chem. Commun.* 2001, 2072-2073; cH. Ott, PhD thesis, Georg-August-Universität Göttingen 2009; dL. Olsson, D. Cremer, *Chem. Phys. Lett.* 1993, *215*, 433-443; eD. Stalke, in *The Chemical Bond I*, Springer, 2016, pp. 57-88; fC. Mohapatra, S. Kundu, A. N. Paesch, R. Herbst-Irmer, D. Stalke, D. M. Andrada, G. Frenking, H. W. Roesky, *J. Am. Chem. Soc.* 2016, *138*, 10429-10432; gE. D. Jemmis, R. Saradha, A. Saieswari, J. Elambalassery, *Indian J. Chem.* 2003, *42*, 2382-2391; hC. B. Yıldız, A. Azizoglu, *Struct. Chem.* 2012, *23*, 1777-1784; iL. C. Pavelka, M. A. Hanson, V. N. Staroverov, K. M. Baines, *Can. J. Chem.* 2014, *93*, 134-142; jM. Bendikov, Y. Apeloig, S. Bukalov, I. Garbuzova, L. Leites, *J. Phys. Chem. A* 2002, *106*, 4880-4885; kX. F. Duan, L. W. Burggraf, L. Huang, *Molecules* 2013, *18*, 8591-8606; lD. Lutters, C. Severin, M. Schmidtmann, T. Müller, *J. Am. Chem. Soc* 2016, *138*, 6061-6067; mY. Wang, M. Chen, Y. Xie, P. Wei, H. F. Schaefer III, G. H. Robinson, *J. Am. Chem. Soc.* 2015, *137*, 8396-8399; nP. V. Bharatam, R. Moudgil, D. Kaur, *Organometallics* 2002, *21*, 3683-3690; oJ.-H. Sheu, M.-D. Su, *Organometallics* 2012, *31*, 3101-3112; pJ.-j. Sui, J. Xu, Y.-h. Ding, *Dalton Trans.* 2016, *45*, 56-60; qA. E. Ketvirtis, D. K. Bohme, A. C. Hopkinson, *J. Phys. Chem. A* 1999, *103*, 11161-11171; rI. Billas, C. Massobrio, M. Boero, M. Parrinello, W. Branz, F. Tast, N. Malinowski, M. Heinebrodt, T. Martin, *J. Chem. Phys.* 1999, *111*, 6787-6796; sJ. M. Rintelman, M. S. Gordon, *J. Chem. Phys.* 2001, *115*, 1795-1803; tB. Niepötter, PhD thesis, Georg-August University School of Science, Göttingen 2015; uC. Eisenhut, T. Szilvási, G. Dübek, N. C. Breit, S. Inoue, *Inorg. Chem.* 2017, *56*, 10061-10069; vS. Roy, K. C. Mondal, T. Mondal, D. Koley, B. Dittrich, H. W. Roesky, *Dalton Trans.* 2015, *44*, 19942-19947; wD. Lutters, A. Merk, M. Schmidtmann, T. Müller, *Inorg. Chem.* 2016, *55*, 9026-9032; xV. Y. Lee, O. A. Gapurenko, Y. Ito, T. Meguro, H. Sugasawa, A. Sekiguchi, R. M. Minyaev, V. I. Minkin, R. H. Herber, H. Gornitzka, *Organometallics* 2016, *35*, 346-356; yG. Martinez-Guajardo, Z. Gómez-Saldoval, D. F. Jana, P. Calaminici, C. Corminboeuf, G. Merino, *PCCP* 2011, *13*, 20615-20619; zP. Jutzi, K. Leszczyńska, B. Neumann, W. W. Schoeller, H. G. Stammler, *Angew. Chem. Int. Ed.* 2009, *48*, 2596-2599; aaV. G. Avakyan, S. L. Guselnikov, V. F. Sidorkin, E. P. Doronina, L. E. Gusel'nikov, *Organometallics* 2009, *28*, 978-989; abP. Ghana, M. I. Arz, U. Das, G. Schnakenburg, A. C. Filippou, *Angew. Chem. Int. Ed.* 2015, *54*, 9980-9985; acF. Shojaie, *Comput. Theor. Chem.* 2017, *1114*, 55-64; adD. Gau, T. Kato, N. Saffon-Merceron, A. De Cózar, F. P. Cossío, A. Baceiredo, *Angew. Chem.* 2010, *122*, 6735-6738; aeD. Gau, R. Rodriguez, T. Kato, N. Saffon-Merceron, A. de Cózar, F. P. Cossío, A. Baceiredo, *Angew. Chem.* 2011, *123*, 1124-1128; afM. Marín-Luna, I. Alkorta, J. Elguero, *J. Phys. Chem. A* 2016, *120*, 648-656; agS.-W. Tang, F.-D. Wang, Y.-H. Li, F. Wang, S.-B. Yang, H. Sun, Y.-F. Chang, R.-S. Wang, *J. Mol. Model.* 2013, *19*, 5579-5586.

[21] aJ.-W. Zou, M. Huang, G.-X. Hu, Y.-J. Jiang, *RSC Advances* 2017, *7*, 10295-10305; bS. Berski, Z. Ciunik, K. Drabent, Z. Latajka, J. Panek, *J. Phys. Chem. B* 2004, *108*, 12327-12332; cJ. Joy, E. D. Jemmis, K. Vidya, *Faraday Discuss.* 2015, *177*, 33-50; dV. Oliveira, E. Kraka, D. Cremer, *Inorg. Chem.* 2017, *56*, 488-502; eH. Xu, J. Cheng, Q. Li, W. Li, *Mol. Phys.* 2016, *114*, 3643-3649; fD. C.

Georgiou, P. Butler, E. C. Browne, D. J. Wilson, J. L. Dutton, *Aust. J. Chem.* 2013, *66*, 1179-1188; gJ. J. Queralt, J. Andrés, M. Canle, J. H. Cobas, J. A. Santaballa, J. R. Sambrano, *Chem. Phys.* 2002, *280*, 1-14; hA. Walczak, B. Brycki, M. Kaczmarek, O. K. Poleshchuk, M. Ostafin, B. Nogaj, *Chem. Phys.* 2006, *325*, 429-436; iA. Petelski, D. J. Duarte, S. Pamies, N. Peruchena, G. Sosa, *Theor. Chem. Acc.* 2016, *135*, 65; jR. D. Parra, *Comput. Theor. Chem.* 2012, *998*, 183-192; kE. Bartashevich, E. Troitskaya, Á. M. Pendás, V. Tsirelson, *Comput. Theor. Chem.* 2015, *1053*, 229-237; lJ. Andrés, X. L. Armesto, M. Canle, M. V. García, D. R. Ramos, J. A. Santaballa, *Org. Biom. Chem.* 2003, *1*, 4323-4328; mM. D. Esrafili, P. Mousavian, *Chem. Phys. Lett.* 2017, *678*, 275-282.

[22] aV. Luana, A. M. Pendas, A. Costales, G. A. Carriedo, F. J. García-Alonso, *J. Phys. Chem. A* 2001, *105*, 5280-5291; bK. K. Pandey, P. Tiwari, P. Patidar, *J. Phys. Chem. A* 2012, *116*, 11753-11762; cK. K. Pandey, P. Tiwari, P. Patidar, *J. Organomet. Chem.* 2013, *740*, 135-140; dK. K. Pandey, P. Tiwari, P. Patidar, S. K. Patidar, R. Vishwakarma, P. K. Bariya, *J. Organomet. Chem.* 2014, *751*, 781-787; eK. K. Pandey, R. Vishwakarma, *J. Organomet. Chem.* 2016, *813*, 84-94; fI. Alkorta, J. Elguero, J. E. Del Bene, *J. Phys. Chem. A* 2013, *117*, 10497-10503; gA. B. Chaplin, J. A. Harrison, P. J. Dyson, *Inorg. Chem.* 2005, *44*, 8407-8417; hJ. E. Del Bene, I. Alkorta, J. Elguero, *J. Phys. Chem. A* 2015, *119*, 11701-11710; iZ. Benkő, R. Streubel, L. Nyulászi, *Dalton Trans.* 2006, 4321-4327; jÁ. Sánchez-González, S. Melchor, J. A. Dobado, B. Silvi, J. Andrés, *J. Phys. Chem. A* 2011, *115*, 8316-8326; kJ. E. Del Bene, I. Alkorta, G. Sánchez-Sanz, J. Elguero, *J. Phys. Chem. A* 2012, *116*, 9205-9213; lV. Jancik, F. Cortés-Guzmán, R. Herbst-Irmer, D. Matínez-Otero, *Chem. Eur. J.* 2017, *23*, 6964-6968; mM. Calichman, A. Derecskei-Kovacs, C. W. Allen, *Inorg. Chem.* 2007, *46*, 2011-2016; nK. Gholivand, H. R. Mahzouni, M. D. Esrafili, *Dalton Trans.* 2012, *41*, 1597-1608; oT. L. Chan, Z. Xie, *Chem. Commun.* 2016, *52*, 7280-7283; pJ. E. Del Bene, I. Alkorta, J. Elguero, G. Sánchez-Sanz, *J. Phys. Chem. A* 2017, *121*, 1362-1370; qL. Ascherl, A. Nordheider, K. S. A. Arachchige, D. B. Cordes, K. Karaghiosoff, M. Bühl, A. M. Slawin, J. D. Woollins, *Chem. Commun.* 2014, *50*, 6214-6216; rM. Demange, L. Boubekeur, A. Auffrant, N. Mézailles, L. Ricard, X. Le Goff, P. Le Floch, *New J. Chem.* 2006, *30*, 1745-1754; sL. F. Ji, A. Y. Li, Z. Z. Li, Z. X. Ge, *J. Mol. Model.* 2015, *22*, 1.

[23] aM. W. Wong, K. B. Wiberg, *J. Am. Chem. Soc.* 1992, *114*, 7527-7535; bK. Haupa, A. Bil, Z. Mielke, *J. Phys. Chem. A* 2015, *119*, 10724-10734; cD. Setiawan, E. Kraka, D. Cremer, *J. Phys. Chem. A* 2015, *119*, 9541-9556; dB. L. Tran, R. Thompson, S. Ghosh, X. Gao, C.-H. Chen, M.-H. Baik, D. J. Mindiola, *Chem. Commun.* 2013, *49*, 2768-2770; eK. K. Pandey, *J. Coord. Chem.* 1990, *22*, 307-313; fT. Pasinszki, G. Bazsó, M. Krebsz, G. Tarczay, *PCCP* 2009, *11*, 9458-9467; gA. J. Bridgeman, G. Cavigliasso, L. R. Ireland, J. Rothery, *Dalton Trans.* 2001, 2095-2108; hS. Feth, PhD thesis, Virginia 1996; iC. M. Breneman, L. W. Weber, *Can. J. Chem.* 1996, *74*, 1271-1282; jL. Lo Presti, A. M. Orlando, L. Loconte, R. Destro, E. Ortoleva, R. Soave, C. Gatti, *Cryst. Growth Des.* 2014, *14*, 4418-4429; kM. D. Esrafili, F. Mohammadian-Sabet, *Mol. Phys.* 2015, *113*, 95-103; lL. M. Azofra, I. Alkorta, S. Scheiner, *J. Phys. Chem. A* 2015, *119*, 535-541; mM. Macernis, *J. Chem.* 2017, *2017*.

[24] aM. F. Lucas, M. C. Michelini, N. Russo, E. Sicilia, *J. Chem. Theory Comput.* 2008, *4*, 397-403; bS. M. Bachrach, *J. Phys. Chem.* 1989, *93*, 7780-7784; cS. M. Bachrach, *J. Comput. Chem.* 1989, *10*, 392-406; dD. Heift, Z. Benkő, H. Grützmacher, *Dalton Trans.* 2014, *43*, 831-840; eA. Doddi, D. Bockfeld, T. Bannenberg, P. G. Jones, M. Tamm, *Angew. Chem.* 2014, *126*, 13786-13790; fL. Liu, D. A. Ruiz, F. Dahcheh, G. Bertrand, *Chem. Commun.* 2015, *51*, 12732-12735; gC. C. Chong, B. Rao, R. Ganguly, Y. Li, R. Kinjo, *Inorg. Chem.* 2017; hM. Yáñez, *J. Phys. Org. Chem.* 1998, *11*, 678-683; iR. A. Rajagopalan, PhD thesis, University of Regina 2014; jS. M. Bachrach, *J. Mol. Struct. THEOCHEM* 1992, *255*, 207-219; kL. Nyulaszi, T. Veszpremi, J. Reffy, *J. Phys. Chem.* 1995, *99*, 10142-10146; lG. v. Frantzius, A. E. Ferao, R. Streubel, *Chem. Sci.* 2013, *4*, 4309-4322; mS. Samiee, N. Kooti, H. Motamedi, R. W. Gable, F. A. Bagherjeri, *Polyhedron* 2015, *98*, 120-130; nH. Joo, PhD thesis, Auburn University, Alabama 2005.

[25] aP. R. Schreiner, H. P. Reisenauer, J. Romanski, G. Mloston, *Angew. Chem. Int. Ed.* 2009, *48*, 8133-8136; bH. S. Rzepa, *J. Chem. Theory Comput.* 2010, *7*, 97-102; cC. Guo, N. Hou, C. Wang, *J. Mol. Model.* 2016, *22*, 1-6; dC. Guo, C. Wang, Y.-h. Ding, *Struct. Chem.* 2014, *25*, 1023-1031; eC. Guo, Z. H. Cui, Y. H. Ding, *Int. J. Quantum Chem* 2013, *113*, 2213-2219; fA. M. Masdeu, A. Ruiz, S. Castillon, C. Claver, P. B. Hitchcock, P. A. Chaloner, C. Bo, J. M. Poblet, P. Sarasa, *Dalton Trans.* 1993, 2689-2696; gA. Vashchenko, A. Abramov, Y. L. Frolov, *J. Mol. Struct. THEOCHEM* 2002, *594*, 107-111; hB. Fresch, F. Remacle, *J. Phys. Chem. C* 2014, *118*, 9790-9800; iC.-R. Lee, L.-Y. Tan, Y. Wang, *J. Phys. Chem. Solids* 2001, *62*, 1613-1628; jI. Sovago, PhD thesis, University of Glasgow 2013; kM. V. Roux, M. Temprado, P. Jiménez, R. Notario, J. S. Chickos, A. F. L. Santos, M. A. Ribeiro da Silva, *J. Phys. Chem. A* 2007, *111*, 11084-11092.

[26] aR. Gaba, M. Bhandari, R. Kakkar, *Adv. Mat. Lett.* 2013, *4*, 769-778; bT. A. Manz, A. E. Fenwick, K. Phomphrai, I. P. Rothwell, K. T. Thomson, *Dalton Trans.* 2005, 668-674; cV. P. Indrakanti, PhD thesis, The Pennsylvania State University 2009; dE. A. Zhurova, V. G. Tsirelson, *Acta Cryst. B.* 2002, *58*, 567-575; eH. M. I. Pritchard, PhD thesis, University of London 2005; fM. Calatayud, P. Mori-

Sánchez, A. Beltrán, A. M. Pendás, E. Francisco, J. Andrés, J. Recio, *Phys. Rev. B.* 2001, *64*, 184113; gN. Seriani, *J. Phys.: Condens. Matter* 2010, *22*, 255502; hK. M. Rosso, J. R. Rustad, G. Gibbs, *J. Phys. Chem. A* 2002, *106*, 8133-8138; iM. Srebro, J. Autschbach, *Chem. Eur. J.* 2013, *19*, 12018-12033; jA. K. Phukan, A. K. Guha, *Inorg. Chem.* 2010, *49*, 9884-9890; kA. Robertazzi, J. A. Platts, *J. Biol. Inorg. Chem.* 2005, *10*, 854-866; lM. Zegke, PhD thesis, University of Edinburgh 2015; mD. Duarte Ruíz, Master's thesis, Universidad EAFIT 2015; nS. Dargouthi, S. Boughdiri, B. Tangour, *Acta Chim. Slov.* 2015, *62*, 445-451; oY. Gong, M. Zhou, S. X. Tian, J. Yang, *J. Phys. Chem. A* 2007, *111*, 6127-6130; pA. Marzouk, B. Madebène, M. E. Alikhani, *J. Phys. Chem. A* 2013, *117*, 4462-4471; qW. Jauch, M. Reehuis, *Acta Crystallogr. Sect. A: Found. Crystallogr.* 2005, *61*, 411-417; rR. A. Evarestov, in *Quantum Chemistry of Solids*, Springer, 2012, pp. 357-488.

[27] aA. J. Bridgeman, G. Cavigliasso, *Faraday Discuss.* 2003, *124*, 239-258; bP. Hejduk, M. Witko, K. Hermann, *Top. Catal.* 2009, *52*, 1105-1115; cJ. K. Bartley, J. S. J. Hargreaves, G. J. Hutchings, J. L. Rico, S. H. Taylor, R. P. K. Wells, D. J. Willock, in *Encyclopedia of Catalysis*, John Wiley & Sons, Inc., 2002; dK. Alexopoulos, P. Hejduk, M. Witko, M.-F. Reyniers, G. B. Marin, *J. Phys. Chem. C* 2010, *114*, 3115-3130; eS. Brandán, C. Socolsky, A. B. Altabef, *Z. Anorg. Allg. Chem.* 2009, *635*, 582-592; fC. Das, P. Adak, S. Mondal, R. Sekiya, R. Kuroda, S. I. Gorelsky, S. K. Chattopadhyay, *Inorg. Chem.* 2014, *53*, 11426-11437; gA. Egorova, V. Tsirel'son, *Russ. J. Inorg. Chem.* 2006, *51*, 941-948; hD. Masuzaki, T. Nagata, F. Mafune, *J. Phys. Chem. A* 2017, *121*, 2079-2085.

[28] aM. E. O'Reilly, PhD thesis, University of Florida 2013; bS. A. Brandán, in *A Structural and Vibrational Investigation into Chromylazide, Acetate, Perchlorate, and Thiocyanate Compounds*, Springer, 2013, pp. 21-56; cM. Sundberg, R. Uggla, R. Sillanpää, K. Zborowski, A. Sánchez-González, J. Matikainen, S. Kaltia, T. Hase, *Open Chem.* 2010, *8*, 486-493; dS. Veliah, K.-h. Xiang, R. Pandey, J. Recio, J. M. Newsam, *J. Phys. Chem. B* 1998, *102*, 1126-1135; eS. A. Brandán, *A Structural and Vibrational Study of the Chromyl Chlorosulfate, Fluorosulfate, and Nitrate Compounds*, Springer Netherlands, 2012; fM. Torrent Sucarrat, P. Gili Trujillo, M. Duran i Portas, M. Solà i Puig, *J. Chem. Phys.* 1996, *104*, 9499-9510; gC.-C. Wang, T.-H. Tang, Y. Wang, *J. Phys. Chem. A* 2000, *104*, 9566-9572; hR. J. Gillespie, I. Bytheway, T.-H. Tang, R. F. Bader, *Inorg. Chem.* 1996, *35*, 3954-3963; iZ. Sun, H. F. Schaefer, Y. Xie, Y. Liu, R. Zhong, *Theor. Chem. Acc.* 2015, *134*, 43; jS. A. Brandán, *J. Mol. Struct. THEOCHEM* 2009, *908*, 19-25; kA. B. Altabef, S. Brandán, *J. Mol. Struct.* 2010, *981*, 146-152; lY. Wang, *J. Chin. Chem. Soc.* 2014, *61*, 27-38; mZ. Sun, H. F. Schaefer, Y. Xie, Y. Liu, R. Zhong, *J. Comput. Chem.* 2014, *35*, 998-1009; nA. D. P. Alexiou, C. C. Decandio, S. D. N. Almeida, M. J. P. Ferreira, P. Romoff, R. C. Rocha, *J. Coord. Chem.* 2017, 1-13.

[29] aM. Jaccob, A. Ansari, B. Pandey, G. Rajaraman, *Dalton Trans.* 2013, *42*, 16518-16526; bP. R. Varadwaj, A. Varadwaj, H. M. Marques, *J. Phys. Chem. A* 2011, *115*, 5592-5601; cD. Rutkowska-Zbik, R. Tokarz-Sobieraj, M. Witko, *J. Chem. Theory Comput.* 2007, *3*, 914-920; dJ. He, Z.-G. Xu, Y.-X. Zeng, X. Xu, L. Yu, Q. Wang, H.-Y. Liu, *Acta Phys.-Chim. Sin.* 2012, *28*, 1658-1664; eA. S. Jepsen, M. Roberson, R. G. Hazell, *Chem. Commun.* 1998, 1599-1600; fG. V. Gibbs, R. Downs, D. F. Cox, K. M. Rosso, N. L. Ross, A. Kirfel, T. Lippmann, W. Morgenroth, T. D. Crawford, *J. Phys. Chem. A* 2008, *112*, 8811-8823; gR. D. Poulsen, A. Bentien, T. Graber, B. Iversen, *Acta Crystallogr. Sect. A: Found. Crystallogr.* 2004, *60*, 382-389; hC. Lecomte, E. Aubert, V. Legrand, F. Porcher, S. Pillet, B. Guillot, C. Jelsch, *Z. Kristallogr.* 2005, *220*, 373-384; iW. Jauch, M. Reehuis, *Phys. Rev. B.* 2003, *67*, 184420; jQ. Chen, L. Ni, Y.-Y. Hu, J. Yao, W.-L. Zhang, *Chin. J. Inorg. Chem.* 2014, *30*, 1481-1488; kM. J. Javan, Z. A. Tehrani, A. Fattahi, M. M. Hashemi, *J. Phys. Org. Chem.* 2012, *25*, 1198-1209; lH. Kavousi, H. Raissi, A. Rezaeifard, M. Jafarpour, *J. Porphyrins Phthalocyanines* 2015, *19*, 1130-1139; mH. Kavousi, A. Rezaeifard, H. Raissi, M. Jafarpour, *J. Porphyrins Phthalocyanines* 2015, *19*, 651-662; nR. A. P. Ribeiro, S. R. de Lazaro, C. Gatti, *RSC Advances* 2016, *6*, 101216-101225.

[30] aP. Chernev, I. Zaharieva, H. Dau, M. Haumann, *J. Biol. Chem.* 2011, *286*, 5368-5374; bW. Yi, L. Yuan, Y. Kun, H. Zhengwen, T. Jing, G. Hong, W. Yong, *J. Biol. Inorg. Chem.* 2015, *20*, 1123-1134; cC. Gómez-Casado, F. Roth-Walter, E. Jensen-Jarolim, A. Díaz-Perales, L. F. Pacios, *J. Mol. Graphics Modell.* 2013, *45*, 111-121; dJ. Overgaard, D. E. Hibbs, E. Rentschler, G. A. Timco, F. K. Larsen, *Inorg. Chem.* 2003, *42*, 7593-7601; eJ. Overgaard, F. K. Larsen, B. Schiøtt, B. B. Iversen, *J. Am. Chem. Soc.* 2003, *125*, 11088-11099; fJ. Overgaard, F. K. Larsen, G. A. Timco, B. B. Iversen, *Dalton Trans.* 2009, 664-671; gV. Berryman, PhD thesis, Dalhousie University 2015; hX. Yang, *Dalton Trans.* 2013, *42*, 11987-11991; iJ. Murgich, H. J. Franco, *J. Phys. Chem. A* 2009, *113*, 5205-5211; jF. A. La Porta, P. H. Ramos, E. C. de Resende, M. C. Guerreiro, J. O. Giacoppo, T. C. Ramalho, J. R. Sambrano, J. Andrés, E. Longo, *Inorg. Chim. Acta* 2014, *416*, 200-206; kZ. Tang, Y. Wang, P. Zhang, *J. Coord. Chem.* 2017, *70*, 417-430; lY. Ling, C. Mills, R. Weber, L. Yang, Y. Zhang, *J. Am. Chem. Soc.* 2010, *132*, 1583-1591; mS. Kavitha, P. Deepa, M. Karthika, R. Kanakaraju, *Polyhedron* 2016, *115*, 193-203; nB. Shin, K. D. Sutherlin, T. Ohta, T. Ogura, E. I. Solomon, J. Cho, *Inorg. Chem.* 2016, *55*, 12391-12399; oJ. Ortega-Castro, J. Frau, R. Casasnovas, D. Fernández, J. Donoso, F. Muñoz, *J. Phys. Chem.*

A 2012, *116*, 2961-2971; pK. K. Pandey, R. Vishwakarma, S. K. Patidar, P. K. Bariya, *Comput. Theor. Chem.* 2015, *1051*, 137-143.

[31] aJ. Overgaard, J. P. Walsh, V. R. Hathwar, M. R. Jørgensen, C. Hoffman, J. A. Platts, R. Piltz, R. E. Winpenny, *Inorg. Chem.* 2014, *53*, 11531-11539; bP. R. Varadwaj, H. M. Marques, *PCCP* 2010, *12*, 2126-2138; cI. Georgieva, N. Trendafilova, *Bulg. Chem. Commun.* 2014, *46*, 196-202; dJ. J. Lee, G. H. Lee, Y. Wang, *Chem. Eur. J.* 2002, *8*, 1821-1832; eL.-C. Wu, T.-C. Weng, I.-J. Hsu, Y.-H. Liu, G.-H. Lee, J.-F. Lee, Y. Wang, *Inorg. Chem.* 2013, *52*, 11023-11033; fC.-R. Lee, C.-C. Wang, K.-C. Chen, G.-H. Lee, Y. Wang, *J. Phys. Chem. A* 1999, *103*, 156-165; gR.-Z. Li, J. Liang, X.-L. Xu, H.-G. Xu, W.-J. Zheng, *Chem. Phys. Lett.* 2013, *575*, 12-17; hH. F. Clausen, PhD thesis, Aarhus University, Denmark 2009; iN. Ghadimi, C. B. Perry, M. A. Fernandes, P. P. Govender, H. M. Marques, *Inorg. Chim. Acta* 2015, *436*, 29-38; jQ.-W. Wang, M. Sun, Y.-N. Wang, X.-F. Qi, X.-M. Li, B. Liu, *Chin. J. Struct. Chem.* 2015, *34*, 393-400; kC. F. Zipp, J. P. Michael, M. A. Fernandes, S. Mathura, C. B. Perry, I. Navizet, P. P. Govender, H. M. Marques, *Inorg. Chem.* 2014, *53*, 4418-4429; lV. Velasco, D. Aguilà, L. Barrios, I. Borilovic, O. Roubeau, J. Ribas-Ariño, M. Fumanal, S. Teat, G. Aromí, *Chem. Sci.* 2015, *6*, 123-131; mJ. Overgaard, M. P. Waller, R. Piltz, J. A. Platts, P. Emseis, P. Leverett, P. A. Williams, D. E. Hibbs, *J. Phys. Chem. A* 2007, *111*, 10123-10133; nW. Jauch, M. Reehuis, *Phys. Rev. B.* 2004, *70*, 195121; oN. Ghadimi, C. B. Perry, P. P. Govender, H. M. Marques, *Inorg. Chim. Acta* 2016, *450*, 269-278; pY.-Z. Zheng, W. Xue, M.-L. Tong, X.-M. Chen, S.-L. Zheng, *Inorg. Chem.* 2008, *47*, 11202-11211; qD. Rutkowska-Zbik, M. Witko, G. Stochel, *Theor. Chem. Acc.* 2008, *120*, 411-419.

[32] aF. Hueso-Ureña, S. B. Jiménez-Pulido, M. P. Fernández-Liencres, M. Fernández-Gómez, M. N. Moreno-Carretero, *Dalton Trans.* 2008, 6461-6466; bJ. Poater, M. Sola, A. Rimola, L. Rodriguez-Santiago, M. Sodupe, *J. Phys. Chem. A* 2004, *108*, 6072-6078; cJ. Overgaard, I. Turel, D. E. Hibbs, *Dalton Trans.* 2007, 2171-2178; dN. Bouhmaida, M. A. Méndez-Rojas, A. n. Pérez-Benítez, G. Merino, B. Fraisse, N. E. Ghermani, *Inorg. Chem.* 2010, *49*, 6443-6452; eW. Jauch, M. Reehuis, *Acta Cryst. B.* 2014, *70*, 983-988; fF. Bertolotti, A. Forni, G. Gervasio, D. Marabello, E. Diana, *Polyhedron* 2012, *42*, 118-127; gZ. Yousefi, H. Eshtiagh-Hosseini, A. Salimi, J. Soleimannejad, *Acta Cryst. C* 2015, *71*, 386-393; hA. Baryshnikova, B. Minaev, G. Baryshnikov, W.-H. Sun, *Russ. J. Inorg. Chem.* 2016, *61*, 588-593; iH.-Y. Zhao, F.-Y. Bai, Y.-H. Xing, Z.-P. Li, Y.-Z. Cao, X.-Q. Zeng, M.-F. Ge, *J. Coord. Chem.* 2010, *63*, 435-447; jG. Zhang, X. Zhao, D. Chen, *J. Phys. Chem. A* 2013, *117*, 10944-10950; kS. Pillet, M. Souhassou, C. Lecomte, P. Rabu, M. Drillon, C. Massobrio, *Phys. Rev. B.* 2006, *73*, 115116; lY. V. Nelyubina, A. A. Korlyukov, I. V. Fedyanin, K. A. Lyssenko, *Inorg. Chem.* 2013, *52*, 14355-14363; mH. H. Monfared, M. Vahedpour, M. M. Yeganeh, M. Ghorbanloo, P. Mayer, C. Janiak, *Dalton Trans.* 2011, *40*, 1286-1294; nT. Lippmann, P. Blaha, N. H. Andersen, H. F. Poulsen, T. Wolf, J. R. Schneider, K.-H. Schwarz, *Acta Crystallogr. Sect. A: Found. Crystallogr.* 2003, *59*, 437-451; oT. Lippmann, J. R. Schneider, *Acta Crystallogr. Sect. A: Found. Crystallogr.* 2000, *56*, 575-584; pM. A. A. Azzellini, M. P. Abbott, A. Machado, M. T. M. Miranda, L. C. Garcia, G. F. Caramori, M. B. Gonçalves, H. M. Petrilli, A. Ferreira, *J. Brazilian Chem. Soc.* 2010, *21*, 1303-1317; qM. Ghambarian, M. Ghashghaee, Z. Azizi, *Physical Chemistry Research* 2017, *5*, 135-152; rR. Golbedaghi, S. Azimi, A. Molaei, M. Hatami, B. Notash, *J. Mol. Struct.* 2017; sF. Zapata, A. Caballero, A. Espinosa, A. Tárraga, P. Molina, *Dalton Trans.* 2010, *39*, 5429-5431; tL. J. Farrugia, D. S. Middlemiss, R. Sillanpää, P. Seppälä, *J. Phys. Chem. A* 2008, *112*, 9050-9067; uA. Fonari, E. S. Leonova, M. Y. Antipin, *Polyhedron* 2011, *30*, 1710-1717; vM. Gryl, *Acta Cryst. B.* 2015, *71*, 392-405; wA. Pakiari, Z. Jamshidi, *J. Phys. Chem. A* 2008, *112*, 7969-7975; xR. Kakkar, R. Grover, P. Gahlot, *Polyhedron* 2006, *25*, 759-766; yF. Turias, M. Solà, L. Falivene, L. Cavallo, A. Poater, *Struct. Chem.* 2016, *1*, 409-417; zN. Lu, Y. Bu, *Can. J. Chem.* 2017; aaJ. S. Woertink, P. J. Smeets, M. H. Groothaert, M. A. Vance, B. F. Sels, R. A. Schoonheydt, E. I. Solomon, *PNAS* 2009, *106*, 18908-18913; abS. Ghosh, S. I. Gorelsky, S. DeBeer George, J. M. Chan, I. Cabrito, D. M. Dooley, J. J. Moura, I. Moura, E. I. Solomon, *J. Am. Chem. Soc.* 2007, *129*, 3955-3965; acY. I. Bolgova, E. Belogolova, V. Belyaeva, E. Timofeeva, O. Trofimova, *Russ. Chem. Bull.* 2017, *4*, 1039-1046; adS. L. Birtles, PhD thesis, Witwatersrand University, Johannesburg, South Africa 2011; aeI. Bytheway, B. N. Figgis, A. N. Sobolev, *Dalton Trans.* 2001, 3285-3294; afM. M. Heravi, T. Hosseinnejad, N. Nazari, *Can. J. Chem.* 2017, *95*, 530-536; agA. Naeimi, S. Saeednia, M. Yoosefian, H. A. Rudbari, V. M. Nardo, *J. Chem. Sci* 2015, *126*, 1321-1328; ahY. Zhang, E. Oldfield, *J. Am. Chem. Soc.* 2008, *130*, 3814-3823; aiC.-H. Guo, H.-S. Wu, X.-M. Zhang, J.-Y. Song, X. Zhang, *J. Phys. Chem. A* 2009, *113*, 6710-6723; ajA. Poater, *J. Phys. Chem. A* 2009, *113*, 9030-9040; akJ. Kožíšek, N. K. Hansen, H. Fuess, *Acta Cryst. B.* 2002, *58*, 463-470; alL. Kucková, L. Bučinský, J. Kožíšek, *J. Mol. Struct.* 2017, *1135*, 186-196; amY. Yu, W. Shen, J. Zhang, R. He, M. Li, *J. Mol. Model.* 2008, *14*, 237-247.

[33] aM. Castillo, E. Romano, H. Lanús, S. Díaz, A. B. Altabef, S. Brandán, *J. Mol. Struct.* 2011, *994*, 202-208; bM. B. Pinto, A. L. Soares, A. Mella Orellana, H. A. Duarte, H. A. De Abreu, *J. Phys. Chem. A* 2017, *121*, 2399-2409; cX. L. Fernández, PhD thesis, Universitat Rovira i Virgili 2004; dJ. R.

Sambrano, L. Gracia, J. Andrés, S. Berski, A. Beltrán, *The Journal of Physical Chemistry A* 2004, *108*, 10850-10860; eE. Drozdz, A. Kolezynski, *RSC Advances* 2017, *7*, 28898-28908.

[34] aY. Ha, A. L. Tenderholt, R. H. Holm, B. Hedman, K. O. Hodgson, E. I. Solomon, *J. Am. Chem. Soc.* 2014, *136*, 9094-9105; bD. Zhou, D. Ma, Y. Wang, X. Liu, X. Bao, *Chem. Phys. Lett.* 2003, *373*, 46-51; cN. Fritsch, PhD thesis, Friedrich-Alexander-Universität Erlangen-Nürnberg 2014; dT.-a. Okamura, Y. Ushijima, Y. Omi, K. Onitsuka, *Inorg. Chem.* 2012, *52*, 381-394; eJ. F. Van der Maelen, J. A. Cabeza, *Inorg. Chem.* 2012, *51*, 7384-7391; fD. W. Brogden, Y. Turov, M. Nippe, G. Li Manni, E. A. Hillard, R. Clérac, L. Gagliardi, J. F. Berry, *Inorg. Chem.* 2014, *53*, 4777-4790; gB. Griffe, G. Agrifoglio, J. L. Brito, F. Ruette, *Catal. Today* 2005, *107–108*, 388-396; hJ. Hein, PhD thesis, Technische Universität München 2015; iS. Berski, F. R. Sensato, V. Polo, J. Andres, V. Safont, *J. Phys. Chem. A* 2010, *115*, 514-522; jA. Sierraalta, *Chem. Phys. Lett.* 1994, *227*, 557-560; kP. Macchi, A. Schultz, F. Larsen, B. Iversen, *J. Phys. Chem. A* 2001, *105*, 9231-9242; lP. Abramov, A. Y. Laricheva, E. Peresypkina, I. Mirzaeva, N. Moroz, M. Sokolov, *Inorg. Chim. Acta* 2012, *383*, 7-12; mA. J. Bridgeman, G. Cavigliasso, *Dalton Trans.* 2001, 3556-3563; nM. M. Cosper, F. Neese, A. V. Astashkin, M. D. Carducci, A. M. Raitsimring, J. H. Enemark, *Inorg. Chem.* 2005, *44*, 1290-1301; oT. A. Bazhenova, K. A. Lyssenko, D. A. Kuznetsov, N. V. Kovaleva, Y. V. Manakin, T. A. Savinykh, A. F. Shestakov, *Polyhedron* 2014, *76*, 108-116.

[35] aF. Miradji, F. Virot, S. Souvi, L. Cantrel, F. Louis, V. Vallet, *J. Phys. Chem. A* 2016, *120*, 606-614; bL. Bučinský, G. E. Büchel, R. Ponec, P. Rapta, M. Breza, J. Kožíšek, M. Gall, S. Biskupič, M. Fronc, K. Schiessl, *Eur. J. Inorg. Chem.* 2013, *2013*, 2505-2519; cA. A. Adeniyi, P. A. Ajibade, *J. Mol. Model.* 2013, *19*, 1325-1338; dM. R. Sundberg, R. Ponec, *Inorg. Chim. Acta* 2006, *359*, 899-906; eT. Zábojníková, R. Cajzl, J. Kljun, Z. Chval, I. Turel, J. V. Burda, *J. Comput. Chem.* 2016, *37*, 1766-1780.

[36] aA. Neuhaus, A. Veldkamp, G. Frenking, *Inorg. Chem.* 1994, *33*, 5278-5286; bU. Pidun, G. Frenking, *Organometallics* 1995, *14*, 5325-5336; cG. M. Chiarella, F. A. Cotton, C. A. Murillo, Q. Zhao, *Inorg. Chem.* 2014, *53*, 2288-2295; dM. Bortoluzzi, C. Evangelisti, F. Marchetti, G. Pampaloni, F. Piccinelli, S. Zacchini, *Dalton Trans.* 2016, *45*, 15342-15349.

[37] aL.-Z. Zhu, C.-Z. Wang, L. Mei, L. Wang, Y.-H. Liu, Z.-T. Zhu, Y.-L. Zhao, Z.-F. Chai, W.-Q. Shi, *CrystEngComm* 2015, *17*, 3031-3040; bV. r. Vallet, U. Wahlgren, I. Grenthe, *J. Phys. Chem. A* 2012, *116*, 12373-12380; cJ.-C. Tourneux, J.-C. Berthet, T. Cantat, P. Thuéry, N. Mézailles, M. Ephritikhine, *J. Am. Chem. Soc.* 2011, *133*, 6162-6165; dS. O. Odoh, G. Schreckenbach, *Inorg. Chem.* 2012, *52*, 245-257; eP. L. Arnold, G. M. Jones, S. O. Odoh, G. Schreckenbach, N. Magnani, J. B. Love, *Nat. Chem.* 2012, *4*, 221-227; fF. Chen, C.-z. Wang, Z.-j. Li, J.-h. Lan, Y.-q. Ji, Z.-f. Chai, *Inorg. Chem.* 2015, *54*, 3829-3834; gB. Teyar, L. Belkhiri, K. Costuas, A. Boucekkine, K. Meyer, *Inorg. Chem.* 2016, *55*, 2870-2881; hA. J. Lewis, H. Yin, P. J. Carroll, E. J. Schelter, *Dalton Trans.* 2014, *43*, 10844-10851; iP. L. Arnold, E. Hollis, G. S. Nichol, J. B. Love, J.-C. Griveau, R. Caciuffo, N. Magnani, L. Maron, L. Castro, A. Yahia, *J. Am. Chem. Soc.* 2013, *135*, 3841-3854; jD. E. Smiles, G. Wu, P. Hrobárik, T. W. Hayton, *J. Am. Chem. Soc.* 2016, *138*, 814-825; kG. A. Shamov, G. Schreckenbach, T. N. Vo, *Chem. Eur. J.* 2007, *13*, 4932-4947; lD. E. Smiles, G. Wu, N. Kaltsoyannis, T. W. Hayton, *Chem. Sci.* 2015, *6*, 3891-3899; mM. Šulka, L. Cantrel, V. Vallet, *J. Phys. Chem. A* 2014, *118*, 10073-10080; nY. Qiu, H. Hu, G. Chen, J. Li, *Sci. China Chem.* 2014, *57*, 426-434; oC.-L. Xiao, C.-Z. Wang, L. Mei, X.-R. Zhang, N. Wall, Y.-L. Zhao, Z.-F. Chai, W.-Q. Shi, *Dalton Trans.* 2015, *44*, 14376-14387; pA. F. F. de Lucena, PhD thesis, Universidade de Lisboa 2015; qD. D. Schnaars, A. J. Gaunt, T. W. Hayton, M. B. Jones, I. Kirker, N. Kaltsoyannis, I. May, S. D. Reilly, B. L. Scott, G. Wu, *Inorg. Chem.* 2012, *51*, 8557-8566; rG. M. Jones, PhD thesis, Edinburgh University 2013; sQ.-Y. Wu, F.-W. Zhai, Y. Liu, L.-Y. Yuan, Z.-F. Chai, W.-Q. Shi, *Dalton Trans.* 2016, *45*, 14988-14997; tA. V. Vologzhanina, A. V. Savchenkov, A. O. Dmitrienko, A. A. Korlyukov, I. S. Bushmarinov, D. V. Pushkin, L. B. Serezhkina, *J. Phys. Chem. A* 2014, *118*, 9745-9752; uV. V. Zhurov, E. A. Zhurova, A. I. Stash, A. A. Pinkerton, *J. Phys. Chem. A* 2011, *115*, 13016-13023; vQ. Huang, PhD thesis, UCL (University College London) 2016; wB. Kosog, PhD thesis, Friedrich-Alexander-Universität Erlangen-Nürnberg 2012; xK.-Q. Hu, L.-Z. Zhu, C.-Z. Wang, L. Mei, Y.-H. Liu, Z.-Q. Gao, Z.-F. Chai, W.-Q. Shi, *Cryst. Growth Des.* 2016, *16*, 4886-4896; yA. Prestianni, L. Joubert, A. Chagnes, G. Cote, C. Adamo, *PCCP* 2011, *13*, 19371-19377; zN. Jiang, W. E. Schwarz, J. Li, *Inorg. Chem.* 2015, *54*, 7171-7180; aaD. Rios, M. del Carmen Michelini, A. F. Lucena, J. Marçalo, J. K. Gibson, *J. Am. Chem. Soc.* 2012, *134*, 15488-15496; abP. Miró, J. Ling, J. Qiu, P. C. Burns, L. Gagliardi, C. J. Cramer, *Inorg. Chem.* 2012, *51*, 8784-8790; acB. Zhang, X. Guo, S. Xie, X. Liu, C. Ling, H. Ma, M. Yu, J. Li, *RSC Advances* 2016, *6*, 81995-82005; adX. Guo, X.-G. Xiong, C. Li, H. Gong, P. Huai, J. Hu, C. Jin, L. Huang, G. Wu, *Inorg. Chim. Acta* 2016, *441*, 117-125; aeH. Xiao, H.-S. Hu, W. H. E. Schwarz, J. Li, *J. Phys. Chem. A* 2010, *114*, 8837-8844.

[38] aI. Kirker, PhD thesis, UCL (University College London) 2013; bG. Gopakumar, B. Sreenivasulu, A. Suresh, C. Brahmmananda Rao, N. Sivaraman, M. Joseph, A. Anoop, *J. Phys. Chem. A* 2016, *120*,

4201-4210; cP. Li, W. Niu, T. Gao, *RSC Advances* 2017, *7*, 4291-4296; dN. Kaltsoyannis, *Dalton Trans.* 2016, *45*, 3158-3162.

[39] aC. W. Spicer, C. F. Lovitt, G. S. Girolami, *Organometallics* 2012, *31*, 4894-4903; bM. Zhang, K. Cheng, H. Yan, Q. Wei, B. Zheng, *Sci. Rep.* 2016, *6*; cD. J. Wolstenholme, K. T. Traboulsee, A. Decken, G. S. McGrady, *Organometallics* 2010, *29*, 5769-5772; dJ. Zhu, E.-L. Zins, M. E. Alikhani, *J. Mol. Model.* 2016, *22*, 294; eX. Wang, Y. Wang, S. Li, Y. Zhang, P. Ma, *J. Phys. Chem. A* 2016, *120*, 5457-5463; fJ. R. De Lile, T. Heine, S. Zhou, *Comput. Mater. Sci.* 2017, *129*, 24-36.

[40] aA. C. Filippou, S. Schneider, G. Schnakenburg, *Angew. Chem. Int. Ed.* 2003, *42*, 4486-4489; bC. J. Czerwinski, E. O. Fetisov, I. P. Gloriozov, Y. F. Oprunenko, *Dalton Trans.* 2013, *42*, 10487-10494; cL.-C. Wu, M. K. Thomsen, S. R. Madsen, M. Schmoekel, M. R. Jørgensen, M.-C. Cheng, S.-M. Peng, Y.-S. Chen, J. Overgaard, B. B. Iversen, *Inorg. Chem.* 2014, *53*, 12489-12498; dE. P. Beaumier, B. S. Billow, A. K. Singh, S. M. Biros, A. L. Odom, *Chem. Sci.* 2016, *7*, 2532-2536; eM. Palusiak, *J. Organomet. Chem.* 2007, *692*, 3866-3873; fJ. Liu, H. Liu, Y. Li, H. Wang, *J. Biol. Phys.* 2014, *40*, 313-323; gD. B. DuPré, *J. Phys. Chem. A* 2009, *113*, 1559-1563.

[41] aG. Vanko, A. Bordage, M. Papai, K. Haldrup, P. Glatzel, A. M. March, G. Doumy, A. Britz, A. Galler, T. Assefa, *J. Phys. Chem. C* 2015, *119*, 5888-5902; bM. P. Mehn, S. D. Brown, T. K. Paine, W. W. Brennessel, C. J. Cramer, J. C. Peters, L. Que Jr, *Dalton Trans.* 2006, 1347-1351; cS. A. Sulway, PhD thesis, Manchester University 2012; dD. Gallego, S. Inoue, B. Blom, M. Driess, *Organometallics* 2014, *33*, 6885-6897; eM.-E. Moret, J. C. Peters, *J. Am. Chem. Soc.* 2011, *133*, 18118-18121; fS. Mebs, B. Braun, R. Kositzki, C. Limberg, M. Haumann, *Inorg. Chem.* 2015, *54*, 11606-11624; gK. L. Fillman, J. A. Przyojski, M. H. Al-Afyouni, Z. J. Tonzetich, M. L. Neidig, *Chem. Sci.* 2015, *6*, 1178-1188; hS. Pillet, V. Legrand, H.-P. Weber, M. Souhassou, J.-F. Létard, P. Guionneau, C. Lecomte, *Z. Kristallogr.* 2008, *223*, 235-249; iR. Ghiasi, M. Daghighi Asl, S. Azmayesh, P. Makkipour, *IJNC* 2014, *1*, 85-96; jS. Ouanounou, W.-C. Shih, T.-G. Ong, G. P. Yap, I. Korobkov, S. Gorelsky, D. Richeson, *Dalton Trans.* 2016, *45*, 14327-14334; kN. Sanina, G. Kozub, T. Kondrat'eva, G. Shilov, D. Korchagin, N. Emel'yanova, O. K. Poleshchuk, A. Chernyak, A. Kulikov, F. Mushenok, *J. Mol. Struct.* 2013, *1041*, 183-189; lM. Ansari, N. Vyas, A. Ansari, G. Rajaraman, *Dalton Trans.* 2015, *44*, 15232-15243; mK. Mierzwicki, S. Berski, Z. Latajka, *Chem. Phys. Lett.* 2011, *507*, 29-36; nX. Sun, J. Du, *J. Theor. Comput. Chem.* 2017, *16*, 1750036; oN. L. Fry, PhD thesis, University of California, Santa Cruz 2012; pY. Luo, Y. Li, H. Yu, J. Zhao, Y. Chen, Z. Hou, J. Qu, *Organometallics* 2011, *31*, 335-344.

[42] aJ. A. Przyojski, M. L. Kiewit, K. L. Fillman, H. D. Arman, Z. J. Tonzetich, *Inorg. Chem.* 2015, *54*, 9637-9645; bA. S. Eisenberg, I. V. Likhtina, V. S. Znamenskiy, R. L. Birke, *J. Phys. Chem. A* 2012, *116*, 6851-6869; cT. Jurca, S. Ouanounou, W.-C. Shih, T.-G. Ong, G. P. A. Yap, I. Korobkov, S. Gorelsky, D. Richeson, *Dalton Trans.* 2016, *45*, 14327-14334; dS. Cong, L. K. Yan, S. Z. Wen, W. Guan, Z. M. Su, *Theor. Chem. Acc.* 2011, *130*, 1043-1053; eS. J. Bonyhady, J. M. Goldberg, N. Wedgwood, T. R. Dugan, A. G. Eklund, W. W. Brennessel, P. L. Holland, *Inorg. Chem.* 2015, *54*, 5148-5150; fL. Zhang, Y. Liu, L. Deng, *J. Am. Chem. Soc.* 2014, *136*, 15525-15528; gP. P. Govender, I. Navizet, C. B. Perry, H. M. Marques, *J. Phys. Chem. A* 2013, *117*, 3057-3068; hY. Jing, X. Chen, X. Yang, *Organometallics* 2015, *34*, 5716-5722; iS. Mebs, J. Henn, B. Dittrich, C. Paulmann, P. Luger, *J. Phys. Chem. A* 2009, *113*, 8366-8378; jS. Mathura, D. Sannasy, A. S. de Sousa, C. B. Perry, I. Navizet, H. M. Marques, *J. Inorg. Biochem.* 2013, *123*, 66-79; kT. Zhu, P. Ning, L. Tang, K. Li, S. Bao, X. Jin, X. Song, X. Zhang, S. Han, *AIP Advances* 2017, *7*, 025012.

[43] aC. J. C. Richter, PhD thesis, Swiss Federal Institute of Technology (ETH), Zurich 2006; bR.-Y. Huang, H. Xu, S.-Y. Ye, G.-H. Wu, X.-Q. Zhao, Y. Wang, G.-X. Liu, *J. Mol. Struct.* 2013, *1036*, 235-243; cT. C. Davenport, PhD thesis, University of California, Berkeley 2012; dZ. Mahdavifar, N. Abbasi, *Physica E* 2014, *56*, 268-276; eA. V. Vologzhanina, A. A. Korlyukov, V. V. Avdeeva, I. N. Polyakova, E. A. Malinina, N. T. Kuznetsov, *J. Phys. Chem. A* 2013, *117*, 13138-13150; fA. V. Vologzhanina, S. V. Kats, L. V. Penkova, V. A. Pavlenko, N. N. Efimov, V. V. Minin, I. L. Eremenko, *Acta Cryst. B.* 2015, *71*, 543-554; gF. Hueso-Urena, S. B. Jimenez-Pulido, M. P. Fernandez-Liencres, M. Fernandez-Gomez, M. N. Moreno-Carretero, *Dalton Trans.* 2008, 6461-6466; hA. Casitas, A. Poater, M. Sola, S. S. Stahl, M. Costas, X. Ribas, *Dalton Trans.* 2010, *39*, 10458-10463; iA. Luna, B. Amekraz, J. Tortajada, *Chem. Phys. Lett.* 1997, *266*, 31-37; jD. E. P. Fonseca, PhD thesis, Universidade Federal de Santa Catarina,Florianópolis 2013; kK. Kisowska, S. Berski, Z. Latajka, *J. Comput. Chem.* 2008, *29*, 2677-2692; lL. Guo, W. Dong, S. Zhang, *RSC advances* 2014, *4*, 41956-41967; mM. Wang, R.-Y. Huang, Z.-Q. Wang, G.-H. Wu, *Inorg. Chem. Commun.* 2015, *56*, 41-44.

[44] aL. Yan, M. Jin, P. Song, Z. Su, *J. Phys. Chem. B* 2010, *114*, 3754-3758; bJ. J. Curley, N. A. Piro, C. C. Cummins, *Inorg. Chem.* 2009, *48*, 9599-9601; cG. H. Timmer, J. F. Berry, *Chem. Sci.* 2012, *3*, 3038-3052; dA. Poater, X. Solans-Monfort, E. Clot, C. Copéret, O. Eisenstein, *Dalton Trans.* 2006, 3077-3087; eA. Eizawa, K. Arashiba, H. Tanaka, S. Kuriyama, Y. Matsuo, K. Nakajima, K. Yoshizawa, Y. Nishibayashi, *Nat. Commun.* 2017, *8*, 14874; fG. C. Fortman, PhD thesis, Miami University 2009; gT. Wagener, G. Frenking, *Inorg. Chem.* 1998, *37*, 1805-1811.

[45] aP. Spuhler, M. Lein, G. Frenking, *Z. Anorg. Allg. Chem.* 2003, *629*, 803-815; bS. Baskaran, P. Balu, C. Sivasankar, *J. Chem. Sci.* 2015, *127*, 83-94; cR. Ghiasi, M. Nemati, A. H. Hakimioun, *J. Chil. Chem. Soc.* 2016, *61*, 2921-2928; dJ. Song, M. B. Hall, *J. Am. Chem. Soc.* 1993, *115*, 327-336.

[46] aT. W. Hayton, *Chem. Commun.* 2013, *49*, 2956-2973; bA. R. Fox, C. C. Cummins, *J. Am. Chem. Soc.* 2009, *131*, 5716-5717; cB. Vlaisavljevich, L. Gagliardi, X. Wang, B. Liang, L. Andrews, I. Infante, *Inorg. Chem.* 2010, *49*, 9230-9235; dD. M. King, F. Tuna, E. J. McInnes, J. McMaster, W. Lewis, A. J. Blake, S. T. Liddle, *Nat. Chem.* 2013, *5*, 482-488; eB. M. Gardner, S. T. Liddle, *Eur. J. Inorg. Chem.* 2013, *2013*, 3753-3770; fQ.-Y. Wu, C.-Z. Wang, J.-H. Lan, C.-L. Xiao, X.-K. Wang, Y.-L. Zhao, Z.-F. Chai, W.-Q. Shi, *Inorg. Chem.* 2014, *53*, 9607-9614; gA. Hervé, Y. Bouzidi, J.-C. Berthet, L. Belkhiri, P. Thuéry, A. Boucekkine, M. Ephritikhine, *Inorg. Chem.* 2014, *53*, 6995-7013; hA. Hervé, Y. Bouzidi, J.-C. Berthet, L. Belkhiri, P. Thuéry, A. Boucekkine, M. Ephritikhine, *Inorg. Chem.* 2015, *54*, 2474-2490; iK. K. Pandey, *Inorg. Chem. Commun.* 2013, *37*, 4-6; jB. Vlaisavljevich, L. Andrews, X. Wang, Y. Gong, G. P. Kushto, B. E. Bursten, *J. Am. Chem. Soc.* 2016, *138*, 893-905; kM. Gregson, E. Lu, D. P. Mills, F. Tuna, E. J. L. McInnes, C. Hennig, A. C. Scheinost, J. McMaster, W. Lewis, A. J. Blake, A. Kerridge, S. T. Liddle, *Nat. Commun.* 2017, *8*, 14137; lB. M. Gardner, S. T. Liddle, *Chem. Commun.* 2015, *51*, 10589-10607; mA. Elkechai, F. Kias, F. Talbi, A. Boucekkine, *J. Mol. Model.* 2014, *20*, 2294; nP. Di Pietro, A. Kerridge, *Inorg. Chem.* 2015, *55*, 573-583; oP. Di Pietro, A. Kerridge, *PCCP* 2016, *18*, 16830-16839; pP. Di Pietro, A. Kerridge, *PCCP* 2017, *19*, 7546-7559; qZ. Huang, L. Sun, Y. Yuan, Y. Li, X. Wang, *Inorg. Chem.* 2016; rN. Qu, D.-M. Su, Q.-Y. Wu, W.-Q. Shi, Q.-J. Pan, *Comput. Theor. Chem.* 2017, *1108*, 29-39; sD. M. King, J. McMaster, F. Tuna, E. J. McInnes, W. Lewis, A. J. Blake, S. T. Liddle, *J. Am. Chem. Soc.* 2014, *136*, 5619-5622; tX. Guo, L. Huang, C. Li, J. Hu, G. Wu, P. Huai, *PCCP* 2015, *17*, 14662-14673.

[47] aD. Tiana, E. Francisco, M. Blanco, P. Macchi, A. Sironi, A. Martín Pendás, *J. Chem. Theory Comput.* 2010, *6*, 1064-1074; bC. L. Firme, D. d. L. Pontes, O. A. Antunes, *Chem. Phys. Lett.* 2010, *499*, 193-198; cO. J. Cooper, D. P. Mills, J. McMaster, F. Tuna, E. McInnes, W. Lewis, A. J. Blake, S. T. Liddle, *Chem. Eur. J.* 2013, *19*, 7071-7083; dB. C. Bailey, H. Fan, J. C. Huffman, M.-H. Baik, D. J. Mindiola, *J. Am. Chem. Soc.* 2007, *129*, 8781-8793; eM. Kamitani, B. Pinter, K. Searles, M. G. Crestani, A. Hickey, B. C. Manor, P. J. Carroll, D. J. Mindiola, *J. Am. Chem. Soc.* 2015, *137*, 11872-11875; fJ. G. Andino, H. Fan, A. R. Fout, B. C. Bailey, M.-H. Baik, D. J. Mindiola, *J. Organomet. Chem.* 2011, *696*, 4138-4146; gM. Lein, J. A. Harrison, A. J. Nielson, *Dalton Trans.* 2013, *42*, 10939-10951; hR. F. Bader, C. F. Matta, *Inorg. Chem.* 2001, *40*, 5603-5611; iH.-F. Li, X.-Y. Kuang, H.-Q. Wang, *Dalton Trans.* 2011, *40*, 4578-4589; jJ. Pilme, B. Silvi, M. E. Alikhani, *J. Phys. Chem. A* 2003, *107*, 4506-4514; kS. Roy, U. Rosenthal, E. D. Jemmis, *Acc. Chem. Res.* 2014, *47*, 2917-2930; lG. V. Oshovsky, B. Hessen, J. N. Reek, B. d. Bruin, *Organometallics* 2011, *30*, 6067-6070; mI. Vidal, S. Melchor, J. A. Dobado, *J. Phys. Chem. A* 2008, *112*, 3414-3423.

[48] aG. Frenking, M. Solà, S. F. Vyboishchikov, *J. Organomet. Chem.* 2005, *690*, 6178-6204; bB. S. Billow, R. D. Bemowski, S. A. DiFranco, R. J. Staples, A. L. Odom, *Organometallics* 2015, *34*, 4567-4573; cC. Makanjee, Master's thesis, Rhodes University 2012; dP. P. Samuel, R. Neufeld, K. C. Mondal, H. W. Roesky, R. Herbst-Irmer, D. Stalke, S. Demeshko, F. Meyer, V. C. Rojisha, S. De, *Chem. Sci.* 2015, *6*, 3148-3153; eC.-C. Wang, Y. Wang, H.-J. Liu, K.-J. Lin, L.-K. Chou, K.-S. Chan, *J. Phys. Chem. A* 1997, *101*, 8887-8901; fH. Li, H. Feng, W. Sun, Y. Xie, R. B. King, H. F. Schaefer III, *New J. Chem.* 2011, *35*, 1718-1729; gB. Boucher, J.-F. Halet, M. Kohout, *Comput. Theor. Chem.* 2015, *1068*, 134-141; hR. Ghiasi, *J. Theor. Comput. Chem.* 2015, *14*, 1550022; iI. Fernández, M. J. Mancheño, R. Vicente, L. A. López, M. A. Sierra, *Chem. Eur. J.* 2008, *14*, 11222-11230; jU. Flierler, PhD thesis, Niedersächsische Staats-und Universitätsbibliothek Göttingen 2009.

[49] aC. Gatti, D. Lasi, *Faraday Discuss.* 2007, *135*, 55-78; bK. Götz, M. Kaupp, H. Braunschweig, D. Stalke, *Chem. Eur. J.* 2009, *15*, 623-632; cJ. F. Van der Maelen, J. A. Cabeza, *Theor. Chem. Acc.* 2016, *135*, 1-11; dX. Yan, X. Li, Z. Sun, Q. Li, L. Meng, *New J. Chem.* 2016, *40*, 1988-1996; eJ. Brugos, J. A. Cabeza, P. Garcia-Alvarez, E. Perez-Carreno, J. F. Van der Maelen, *Dalton Trans.* 2017, *46*, 4009-4017; fD. A. Valyaev, R. Brousses, N. Lugan, I. Fernández, M. A. Sierra, *Chem. Eur. J.* 2011, *17*, 6602-6605; gY. Ortin, N. Lugan, S. Pillet, M. Souhassou, C. Lecomte, K. Costuas, J.-Y. Saillard, *Inorg. Chem.* 2005, *44*, 9607-9609.

[50] aY. Chen, PhD thesis, Philipps-Universität Marburg 2000; bT. R. Kégl, L. Kollár, T. Kégl, *J. Quantum Chem.* 2014, *2014*; cJ. Uddin, C. Boehme, G. Frenking, *Organometallics* 2000, *19*, 571-582; dP. Parameswaran, G. Frenking, *Chem. Eur. J.* 2009, *15*, 8807-8816; eA. I. Konovalov, PhD thesis, Institut Català d'Investigació Química (ICIQ) 2015; fA. Rossin, PhD thesis, Cardiff University 2004; gB. Rohrmoser, G. Eickerling, M. Presnitz, W. Scherer, V. Eyert, R.-D. Hoffmann, U. C. Rodewald, C. Vogt, R. Pöttgen, *J. Am. Chem. Soc.* 2007, *129*, 9356-9365; hS. A. Decker, PhD thesis, University of Alberta 2000; iM. A. Alvarez, M. E. García, D. García-Vivó, A. Ramos, M. A. Ruiz, *Inorg. Chem.* 2012, *51*, 3698-3706; jH. Chevreau, C. Martinsky, A. Sevin, C. Minot, B. Silvi, *New J. Chem.* 2003, *27*, 1049-1053; kL. J. Farrugia, C. Evans, M. Tegel, *J. Phys. Chem. A* 2006, *110*, 7952-7961; lP. Macchi, L.

Garlaschelli, A. Sironi, *J. Am. Chem. Soc.* 2002, *124*, 14173-14184; mH. Braunschweig, Q. Ye, A. Vargas, K. Radacki, A. Damme, *Angew. Chem. Int. Ed.* 2013, *52*, 10657-10660; nE. P. Malar, *Theor. Chem. Acc.* 2005, *114*, 213-221.

[51] aJ. Overgaard, J. A. Platts, B. B. Iversen, *Acta Cryst. B.* 2009, *65*, 715-723; bL. J. Farrugia, C. Evans, *C.R. Chim.* 2005, *8*, 1566-1583; cC. B. Perry, N. Shin, M. A. Fernandes, H. M. Marques, *Dalton Trans.* 2013, *42*, 7555-7561; dT. Kégl, F. Ungváry, *J. Organomet. Chem.* 2007, *692*, 1825-1833; eS. P. George, PhD thesis, California University, San Diego 2014.

[52] aT. Kégl, R. Ponec, L. Kollár, *J. Phys. Chem. A* 2011, *115*, 12463-12473; bR. Ghiasi, A. H. Hakimyoon, *J. Mex. Chem. Soc.* 2012, *56*, 100-104; cG. Eickerling, R. Mastalerz, V. Herz, W. Scherer, H.-J. Himmel, M. Reiher, *J. Chem. Theory Comput.* 2007, *3*, 2182-2197; dF. Koch, H. Schubert, P. Sirsch, A. Berkefeld, *Dalton Trans.* 2015, *44*, 13315-13324; eW. Scherer, G. Eickerling, D. Shorokhov, E. Gullo, G. S. McGrady, P. Sirsch, *New J. Chem.* 2006, *30*, 309-312; fJ. Du, X. Sun, J. Chen, G. Jiang, *RSC Advances* 2014, *4*, 44786-44794; gR. Ghiasi, S. Hashemian, O. Irajee, *J. Korean Chem. Soc.* 2011, *55*; hW. Scherer, C. Hauf, M. Presnitz, E. W. Scheidt, G. Eickerling, V. Eyert, R. D. Hoffmann, U. C. Rodewald, A. Hammerschmidt, C. Vogt, *Angew. Chem. Int. Ed.* 2010, *49*, 1578-1582; iW. H. Harman, T. P. Lin, J. C. Peters, *Angew. Chem. Int. Ed.* 2014, *53*, 1081-1086; jP. Macchi, D. M. Proserpio, A. Sironi, *J. Am. Chem. Soc.* 1998, *120*, 1447-1455; kN. Hebben, H. J. Himmel, G. Eickerling, C. Herrmann, M. Reiher, V. Herz, M. Presnitz, W. Scherer, *Chem. Eur. J.* 2007, *13*, 10078-10087; lL. S. Jongbloed, PhD thesis, Amsterdam University 2016.

[53] aC. Boehme, G. Frenking, *Organometallics* 1998, *17*, 5801-5809; bM. Boehme, G. Frenking, M. T. Reetz, *Organometallics* 1994, *13*, 4237-4245; cP. Balu, V. Kannappan, R. Kumar, *Advances in Chemistry* 2016, *2016*; dA. Poater, M. Duran, P. Jaque, A. Toro-Labbe, M. Sola, *J. Phys. Chem. B* 2006, *110*, 6526-6536; eC. M. Wyss, PhD thesis, Georgia Institute of Technology 2015; fG. Wu, Y.-W. Li, H.-W. Xiang, Y.-Y. Xu, Y.-H. Sun, H. Jiao, *J. Mol. Struct. THEOCHEM* 2003, *637*, 101-107; gC. Calle, A. Schweiger, G. Mitrikas, *Inorg. Chem.* 2007, *46*, 1847-1855; hQ. Meng, M. Li, *J. Mol. Struct. THEOCHEM* 2006, *765*, 13-20; iM. E. Alikhani, L. Manceron, *J. Mol. Spectrosc.* 2015, *310*, 32-38; jZ. Huang, Y. Yuan, L. Sun, X. Wang, Y. Li, *RSC Advances* 2016, *6*, 84016-84024; kG. Sánchez-Sanz, I. Alkorta, J. Elguero, M. Yáñez, O. Mó, *PCCP* 2012, *14*, 11468-11477; lK. Freitag, H. Banh, C. Gemel, R. W. Seidel, S. Kahlal, J.-Y. Saillard, R. A. Fischer, *Chem. Commun.* 2014, *50*, 8681-8684; mC. Geng, J. Li, T. Weiske, M. Schlangen, S. Shaik, H. Schwarz, *J. Am. Chem. Soc.* 2017, *139*, 1684-1689; nJ. R. Shakirova, E. V. Grachova, V. V. Gurzhiy, I. O. Koshevoy, A. S. Melnikov, O. V. Sizova, S. P. Tunik, A. Laguna, *Dalton Trans.* 2012, *41*, 2941-2949; oX.-B. Wang, Y.-L. Wang, J. Yang, X.-P. Xing, J. Li, L.-S. Wang, *J. Am. Chem. Soc.* 2009, *131*, 16368-16370; pP. I. Dem 'yanov, R. M. Gschwind, *Organometallics* 2006, *25*, 5709-5723; qK. Lammertsma, A. W. Ehlers, M. L. McKee, *J. Am. Chem. Soc.* 2003, *125*, 14750-14759; rY. Liu, Z. Luo, J. Z. Zhang, F. Xia, *J. Phys. Chem. A* 2016, *120*, 6485-6492; sA. C. Tsipis, D. N. Gkarmpounis, *J. Comput. Chem.* 2012, *33*, 2318-2331; tA. C. Tsipis, *Organometallics* 2012, *31*, 7206-7212; uN. Grzegorzek, M. Pawlicki, L. Szterenberg, L. Latos-Grażyński, *J. Am. Chem. Soc.* 2009, *131*, 7224-7225; vU. Jayarathne, T. J. Mazzacano, S. Bagherzadeh, N. P. Mankad, *Organometallics* 2013, *32*, 3986-3992; wN. Grzegorzek, E. b. Nojman, L. Szterenberg, L. Latos-Grażyński, *Inorg. Chem.* 2013, *52*, 2599-2606; xR. Grande-Aztatzi, J. M. Mercero, E. Matito, G. Frenking, J. M. Ugalde, *PCCP* 2017, *19*, 9669-9675; yG. Huang, Y. Xia, *ACS Catalysis* 2015, *5*, 859-868.

[54] aX. Liu, D. R. Salahub, *Can. J. Chem.* 2015, *94*, 282-292; bH.-S. Hu, Y.-H. Qiu, X.-G. Xiong, W. H. E. Schwarz, J. Li, *Chem. Sci.* 2012, *3*, 2786-2796; cM. E. García, D. García-Vivó, M. A. Ruiz, S. Alvarez, G. Aullón, *Organometallics* 2007, *26*, 5912-5921; dN. Curado, M. Carrasco, E. Álvarez, C. Maya, R. Peloso, A. Rodríguez, J. López-Serrano, E. Carmona, *J. Am. Chem. Soc.* 2015, *137*, 12378-12387; eT. R. Cundari, M. S. Gordon, *Organometallics* 1992, *11*, 55-63; fR. Stegmann, A. Neuhaus, G. Frenking, *J. Am. Chem. Soc.* 1993, *115*, 11930-11938; gM. Carrasco, M. Faust, R. Peloso, A. Rodríguez, J. López-Serrano, E. Álvarez, C. Maya, P. P. Power, E. Carmona, *Chem. Commun.* 2012, *48*, 3954-3956; hT. A. N. Nguyen, T. P. L. Huynh, V. T. Pham, *J. Vietnam. Environ.* 2014, *6*, 142-148; iR. Ramalakshmi, K. Maheswari, D. Sharmila, A. Paul, T. Roisnel, J.-F. Halet, S. Ghosh, *Dalton Trans.* 2016, *45*, 16317-16324; jV. Caliman, P. B. Hitchcock, J. F. Nixon, L. Nyulaszi, N. Sakarya, *Chem. Commun.* 1997, 1305-1306; kM. a. Alonso, M. A. Alvarez, M. E. García, D. García-Vivó, M. A. Ruiz, *Inorg. Chem.* 2010, *49*, 8962-8976; lK. K. Pandey, M. Lein, G. Frenking, *J. Am. Chem. Soc.* 2003, *125*, 1660-1668; mM. Ray, PhD thesis, Kyoto University 2009; nM. Alvarez, M. E. García, D. García-Vivó, S. Menéndez, M. A. Ruiz, *Chem. Eur. J.* 2016, *22*, 8763-8767; oM. A. Alvarez, M. Casado-Ruano, M. E. García, D. García-Vivó, M. A. Ruiz, *Inorg. Chem.* 2017, *56*, 11336-11351.

[55] aJ. A. Cabeza, J. F. Van der Maelen, S. García-Granda, *Organometallics* 2009, *28*, 3666-3672; bJ. Małecki, A. Maroń, M. Serda, M. Dolezal, J. Polański, *Polyhedron* 2012, *41*, 104-114; cA. May, N. Ouddai, *J. Struct. Chem.* 2012, *53*, 220-227; dR. Ghiasi, M. Manochehri, N. Yadegari, *Russ. J. Phys. Chem. A* 2013, *87*, 1506-1514; eG. Occhipinti, H.-R. Bjørsvik, V. R. Jensen, *J. Am. Chem. Soc.* 2006,

128, 6952-6964; fP. R. Remya, C. H. Suresh, *Dalton Trans.* 2015, *44*, 17660-17672; gA. O. Borissova, M. Y. Antipin, D. S. Perekalin, K. A. Lyssenko, *CrystEngComm* 2008, *10*, 827-832; hG. Gervasio, R. Bianchi, D. Marabello, *Chem. Phys. Lett.* 2005, *407*, 18-22.

[56] aS. F. Vyboishchikov, G. Frenking, *Chem. Eur. J.* 1998, *4*, 1428-1438; bR. Stegmann, G. Frenking, *Organometallics* 1995, *14*, 5308-5315; cP. R. Remya, C. H. Suresh, *Dalton Trans.* 2016, *45*, 1769-1778; dS. F. Vyboishchikov, G. Frenking, *Chem. Eur. J.* 1998, *4*, 1439-1448; eE. D. Jemmis, K. T. Giju, *Organometallics* 1997, *16*, 1425-1429; fG. Frenking, U. Pidun, *Dalton Trans.* 1997, 1653-1662; gM. A. Alvarez, M. E. García, D. García-Vivó, M. A. Ruiz, M. F. Vega, *Dalton Trans.* 2014, *43*, 16044-16055; hM. A. Alvarez, M. E. García, D. García-Vivó, M. A. Ruiz, M. F. Vega, *Organometallics* 2010, *29*, 512-515; iX. Sun, J. Du, *Eur. Phys. J. D* 2014, *68*, 211; jT. Chen, X.-H. Zhang, C. Wang, S. Chen, Z. Wu, L. Li, K. R. Sorasaenee, J. B. Diminnie, H. Pan, I. A. Guzei, *Organometallics* 2005, *24*, 1214-1224; kA. C. Filippou, A. I. Philippopoulos, G. Schnakenburg, *Organometallics* 2003, *22*, 3339-3341.

[57] aL. Petit, L. Joubert, P. Maldivi, C. Adamo, *J. Am. Chem. Soc.* 2006, *128*, 2190-2191; bJ. T. Lyon, H.-S. Hu, L. Andrews, J. Li, *PNAS* 2007, *104*, 18919-18924; cZ. R. Turner, PhD thesis, Edinburgh University 2011; dC.-Z. Wang, J. K. Gibson, J.-H. Lan, Q.-Y. Wu, Y.-L. Zhao, J. Li, Z.-F. Chai, W.-Q. Shi, *Dalton Trans.* 2015, *44*, 17045-17053; eE. Di Santo, M. d. C. Michelini, N. Russo, *Organometallics* 2009, *28*, 3716-3726; fI. Kirker, N. Kaltsoyannis, *Dalton Trans.* 2011, *40*, 124-131; gP. L. Arnold, Z. R. Turner, N. Kaltsoyannis, P. Pelekanaki, R. M. Bellabarba, R. P. Tooze, *Chem. Eur. J.* 2010, *16*, 9623-9629; hA. H. Greif, P. Hrobárik, J. Autschbach, M. Kaupp, *PCCP* 2016, *18*, 30462-30474; iM. F. Zalazar, V. M. Rayón, A. Largo, *J. Chem. Phys.* 2013, *138*, 114307.

[58] aA. Oppermann, R. Dick, C. Wehrhahn, U. Flörke, S. Herres-Pawlis, G. Henkel, *Eur. J. Inorg. Chem.* 2016, *2016*, 3744-3755; bS. A. Sadjadi, PhD thesis, Hong Kong University 2013; cS. Dinda, A. G. Samuelson, *Chem. Eur. J.* 2012, *18*, 3032-3042; dP. I. Dem'yanov, P. M. Polestshuk, V. V. Kostin, *Chem. Eur. J.* 2017, *23*, 3257-3261; eX. Li, J. Cai, *Mol. Phys.* 2017, *115*, 1544-1554.

[59] aH. F. Dos Santos, D. Paschoal, J. V. Burda, *Chem. Phys. Lett.* 2012, *548*, 64-70; bX. Li, *J. Comput. Chem.* 2014, *35*, 923-931; cP. Jerabek, PhD thesis, Philipps-Universität Marburg 2015; dT. B. Anisimova, M. A. Kinzhalov, M. F. C. G. da Silva, A. S. Novikov, V. Y. Kukushkin, A. J. Pombeiro, K. V. Luzyanin, *New J. Chem.* 2017, *41*, 3246-3250; eW.-L. Li, Y. Li, C.-Q. Xu, X.-B. Wang, E. Vorpagel, J. Li, *Inorg. Chem.* 2015, *54*, 11157-11167; fH.-T. Liu, X.-G. Xiong, P. D. Dau, Y.-L. Wang, J. Li, L.-S. Wang, *Chem. Sci.* 2011, *2*, 2101-2108.

[60] aY. Sunada, S. Ishida, F. Hirakawa, Y. Shiota, K. Yoshizawa, S. Kanegawa, O. Sato, H. Nagashima, T. Iwamoto, *Chem. Sci.* 2016, *7*, 191-198; bJ. L. Kneebone, V. E. Fleischauer, S. L. Daifuku, A. A. Shaps, J. M. Bailey, T. E. Iannuzzi, M. L. Neidig, *Inorg. Chem.* 2015, *55*, 272-282; cB. Stadelmann, J. Bender, D. Förster, W. Frey, M. Nieger, D. Gudat, *Dalton Trans.* 2015, *44*, 6023-6031; dS. Cenedese, PhD thesis, Milan University 2011.

[61] aM. Feliz, R. Llusar, J. Andrés, S. Berski, B. Silvi, *New J. Chem.* 2002, *26*, 844-850; bM. A. Alvarez, M. E. García, D. García-Vivó, R. Lozano, A. Ramos, M. A. Ruiz, *Inorg. Chem.* 2014, *53*, 11261-11273; cM. Fleischmann, PhD thesis, Universität Regensburg 2016.

[62] aG. Gibbs, R. T. Downs, C. Prewitt, K. M. Rosso, N. Ross, D. F. Cox, *J. Phys. Chem. B* 2005, *109*, 21788-21795; bS. A. Vedha, R. V. Solomon, P. Venuvanalingam, *PCCP* 2014, *16*, 10698-10707; cY. Aray, D. Vega, J. Rodriguez, A. B. Vidal, M. E. Grillo, S. Coll, *J. Phys. Chem. B* 2009, *113*, 3058-3070; dD. C. Onwudiwe, M. M. Kabanda, E. E. Ebenso, E. Hosten, *J. Chem. Sci.* 2016, *128*, 1081-1093.

[63] aA. Morales-García, A. L. Soares Jr, E. C. Dos Santos, H. A. de Abreu, H. l. A. Duarte, *J. Phys. Chem. A* 2014, *118*, 5823-5831; bR. Ghiasi, *J. Sulfur. Chem.* 2012, *33*, 93-100; cK. M. Rosso, M. Hochella, *Surf. Sci.* 1999, *423*, 364-374; dX. Zhang, X. Li, Y. Zeng, S. Zheng, L. Meng, *Dalton Trans.* 2015, *44*, 1283-1291; eJ. Andrés, S. Berski, M. Feliz, R. Llusar, F. Sensato, B. Silvi, *C.R. Chim.* 2005, *8*, 1400-1412; fB. Ni, J. R. Kramer, N. H. Werstiuk, *J. Phys. Chem. A* 2003, *107*, 2890-2897; gA. Pakiari, Z. Jamshidi, *J. Phys. Chem. A* 2010, *114*, 9212-9221.

[64] aT. Li, PhD thesis, The Ohio State University 2016; bS. I. Gorelsky, *J. Chem. Theory Comput.* 2012, *8*, 908-914; cH. S. Kang, *J. Phys. Chem. A* 2005, *109*, 9292-9298; dS. Ilango, B. Vidjayacoumar, S. Gambarotta, S. I. Gorelsky, *Inorg. Chem.* 2008, *47*, 3265-3273; eH. Wang, Z. Sun, Y. Xie, R. B. King, H. F. Schaefer III, *Inorg. Chem.* 2011, *50*, 9256-9265; fH. Wang, H. Wang, R. B. King, H. F. Schaefer, *J. Comput. Chem.* 2016, *37*, 250-260; gF. R. Wagner, A. I. Baranov, Y. Grin, M. Kohout, *Z. Anorg. Allg. Chem.* 2013, *639*, 2025-2035; hS. G. Kozlova, M. R. Ryzhikov, V. A. Slepkov, S. P. Gabuda, *Polyhedron* 2014, *70*, 59-64.

[65] aL. J. Farrugia, P. Macchi, in *Electron Density and Chemical Bonding I*, Springer, 2010, pp. 127-158; bL.-C. Wu, C.-W. Hsu, Y.-C. Chuang, G.-H. Lee, Y.-C. Tsai, Y. Wang, *J. Phys. Chem. A* 2011, *115*, 12602-12615; cF.-Y. Li, L. Chen, C.-Y. Mou, S.-M. Peng, Y. Wang, *Int. J. Mol. Sci.* 2002, *3*, 38-55; dG. La Macchia, G. Li Manni, T. Todorova, M. Brynda, F. Aquilante, B. Roos, L. Gagliardi, *Inorg. Chem.* 2010, *49*, 5216-5222; eK. A. Kreisel, G. P. Yap, O. Dmitrenko, C. R. Landis, K. H. Theopold, *J. Am. Chem. Soc.* 2007, *129*, 14162-14163; fG. La Macchia, F. Aquilante, V. Veryazov, B. O. Roos, L.

Gagliardi, *Inorg. Chem.* 2008, *47*, 11455-11457; gG. La Macchia, PhD thesis, Geneva University 2010; hA. Noor, G. Glatz, R. Müller, M. Kaupp, S. Demeshko, R. Kempe, *Nat. Chem.* 2009, *1*, 322-325; iH. Wang, D. Die, H. Wang, Y. Xie, R. B. King, H. F. Schaefer, *Theor. Chem. Acc.* 2014, *133*, 1459; jY. L. Huang, D. Y. Lu, H. C. Yu, J. S. K. Yu, C. W. Hsu, T. S. Kuo, G. H. Lee, Y. Wang, Y. C. Tsai, *Angew. Chem. Int. Ed.* 2012, *51*, 7781-7785; kA. Mitschler, B. Rees, R. Wiest, M. Benard, *J. Am. Chem. Soc.* 1982, *104*, 7501-7509; lB. O. Roos, A. C. Borin, L. Gagliardi, *Angew. Chem.* 2007, *119*, 1491-1494; mY. I. Kurokawa, Y. Nakao, S. Sakaki, *J. Phys. Chem. A* 2009, *113*, 3202-3209; nF. Ferrante, L. Gagliardi, B. E. Bursten, A. P. Sattelberger, *Inorg. Chem.* 2005, *44*, 8476-8480; oR. J. Eisenhart, R. K. Carlson, K. M. Boyle, L. Gagliardi, C. C. Lu, *Inorg. Chim. Acta* 2015, *424*, 336-344; pP. Atha, I. Hillier, M. Guest, *Mol. Phys.* 1982, *46*, 437-448; qP. Macchi, D. Donghi, A. Sironi, *J. Am. Chem. Soc.* 2005, *127*, 16494-16504; rL. Gagliardi, in *The Chemical Bond: Chemical Bonding Across the Periodic Table*, Wiley-VCH Verlag GmbH & Co. KGaA, 2014, pp. 253-268; sR. J. Eisenhart, P. A. Rudd, N. Planas, D. W. Boyce, R. K. Carlson, W. B. Tolman, E. Bill, L. Gagliardi, C. C. Lu, *Inorg. Chem.* 2015, *54*, 7579-7592; tM. Brynda, L. Gagliardi, P. O. Widmark, P. P. Power, B. O. Roos, *Angew. Chem. Int. Ed.* 2006, *45*, 3804-3807; uA. Noor, T. Bauer, T. K. Todorova, B. Weber, L. Gagliardi, R. Kempe, *Chem. Eur. J.* 2013, *19*, 9825-9832; vG. Merino, K. J. Donald, J. S. D'Acchioli, R. Hoffmann, *J. Am. Chem. Soc.* 2007, *129*, 15295-15302; wN. Bouchakri, PhD thesis, Universite de Constantine 1 2014; xZ. Sun, H. F. Schaefer III, Y. Xie, Y. Liu, R. Zhong, *Mol. Phys.* 2013, *111*, 2523-2535; yM. Brynda, L. Gagliardi, B. O. Roos, *Chem. Phys. Lett.* 2009, *471*, 1-10; zG. Li Manni, A. L. Dzubak, A. Mulla, D. W. Brogden, J. F. Berry, L. Gagliardi, *Chem. Eur. J.* 2012, *18*, 1737-1749; aaK. Albahily, V. Fomitcheva, S. Gambarotta, I. Korobkov, M. Murugesu, S. I. Gorelsky, *J. Am. Chem. Soc.* 2011, *133*, 6380-6387; abC. Schwarzmaier, PhD thesis, Universität Regensburg 2014; acS. Gong, Q. Luo, X. Feng, Q.-s. Li, Y. Xie, R. B. King, H. F. Schaefer III, *PCCP* 2015, *17*, 20100-20113.

[66] aL. Dahl, R. Rundle, *Acta Cryst.* 1963, *16*, 419-426; bH. Li, H. Feng, W. Sun, Q. Fan, Y. Xie, R. B. King, H. F. Schaefer III, *Mol. Phys.* 2012, *110*, 1637-1650; cX. Chen, Q. Du, R. Jin, H. Feng, Y. Xie, R. Bruce King, *New J. Chem.* 2011, *35*, 1117-1127; dT. Gribanova, O. Gapurenko, R. Minyaev, V. Minkin, *Russ. Chem. Bull.* 2015, *64*, 540-550; eL. Xu, Q.-s. Li, Y. Xie, R. B. King, H. F. Schaefer, *Inorg. Chim. Acta* 2010, *363*, 3538-3549; fL. Fohlmeister, S. Liu, C. Schulten, B. Moubaraki, A. Stasch, J. D. Cashion, K. S. Murray, L. Gagliardi, C. Jones, *Angew. Chem. Int. Ed.* 2012, *51*, 8294-8298; gZ. Sun, H. Wang, Y. Xie, R. B. King, H. F. Schaefer Iii, *Dalton Trans.* 2010, *39*, 10702-10711.

[67] aC. M. Zall, D. Zherebetskyy, A. L. Dzubak, E. Bill, L. Gagliardi, C. C. Lu, *Inorg. Chem.* 2011, *51*, 728-736; bG. La Macchia, L. Gagliardi, P. P. Power, M. Brynda, *J. Am. Chem. Soc.* 2008, *130*, 5104-5114; cC. Gatti, *Phys. Scr.* 2013, *87*, 048102; dR. Ponec, G. Lendvay, J. Chaves, *J. Comput. Chem.* 2008, *29*, 1387-1398; eH. Wang, Y. Xie, R. B. King, H. F. Schaefer, *J. Am. Chem. Soc.* 2006, *128*, 11376-11384; fS. Gong, C. Wang, Q.-S. Li, Y. Xie, R. B. King, *J. Coord. Chem.* 2012, *65*, 2459-2477; gJ. Deng, Q.-s. Li, Y. Xie, R. B. King, H. Schaefer III, *New J. Chem.* 2013, *37*, 2902-2910; hX. Gao, S. Gong, N. Li, R. B. King, *New J. Chem.* 2015, *39*, 4939-4947; iL. Zhou, G. Li, Q.-S. Li, Y. Xie, R. B. King, *Can. J. Chem.* 2014, *92*, 750-757; jS. T. Liddle, *Molecular Metal-Metal Bonds: Compounds, Synthesis, Properties*, Wiley, 2015; kY. Zeng, S. Wang, H. Feng, Y. Xie, R. B. King, *Int. J. Mol. Sci.* 2011, *12*, 2216-2231; lH. Lei, J.-D. Guo, J. C. Fettinger, S. Nagase, P. P. Power, *J. Am. Chem. Soc.* 2010, *132*, 17399-17401; mG. Li, L. Liu, J. Wang, Q.-s. Li, Y. Xie, R. B. King, *Transition Met. Chem.* 2013, *38*, 617-625; nL. Bertini, C. Greco, L. De Gioia, P. Fantucci, *J. Phys. Chem. A* 2009, *113*, 5657-5670; oA. D. Phillips, A. Ienco, J. Reinhold, H. C. Böttcher, C. Mealli, *Chem. Eur. J.* 2006, *12*, 4691-4701; pJ. Zhang, J. Liu, *Russ. J. Phys. Chem. A* 2016, *90*, 2564-2570; qP. Mori-Sánchez, A. Costales, M. Blanco, A. M. Pendás, V. Luaña, Departamento de Química Física y Analítica. Universidad de Oviedo, 1999; rJ. C. Silva, H. A. De Abreu, H. A. Duarte, *RSC Advances* 2015, *5*, 2013-2023; sC. E. Hoyer, G. L. Manni, D. G. Truhlar, L. Gagliardi, *J. Chem. Phys.* 2014, *141*, 204309.

[68] aN. K. Shee, R. Verma, D. Kumar, D. Datta, *Comput. Theor. Chem.* 2015, *1061*, 1-5; bC.-N. Lin, W.-T. Jhu, M. Shieh, *Chem. Commun.* 2014, *50*, 1134-1136; cS. Torelli, M. Orio, J. Pécaut, H. Jamet, L. Le Pape, S. Ménage, *Angew. Chem.* 2010, *122*, 8425-8428; dJ. Zhou, W.-N. Wang, K.-N. Fan, *Chem. Phys. Lett.* 2006, *424*, 247-251; eS. I. Gorelsky, X. Xie, Y. Chen, J. A. Fee, E. I. Solomon, *J. Am. Chem. Soc.* 2006, *128*, 16452-16453; fJ. M. Goodwin, P.-C. Chiang, M. Brynda, K. Penkina, M. M. Olmstead, T. E. Patten, *Dalton Trans.* 2007, 3086-3092; gF. Lu, X. Li, Y. Zeng, X. Zhang, L. Meng, *New J. Chem.* 2014, *38*, 5786-5792; hS. Pan, R. Saha, S. Mandal, P. K. Chattaraj, *PCCP* 2016, *18*, 11661-11676.

[69] aM. Tayebani, K. Feghali, S. Gambarotta, G. P. Yap, *Inorg. Chem.* 2001, *40*, 1399-1401; bR. Llusar, A. Beltrán, J. Andrés, F. Fuster, B. Silvi, *J. Phys. Chem. A* 2001, *105*, 9460-9466; cL. Tang, Q. Luo, Q.-s. Li, Y. Xie, R. B. King, H. F. Schaefer III, *J. Chem. Theory Comput.* 2012, *8*, 862-874; dX. Feng, Y. Xie, R. B. King, *J. Organomet. Chem.* 2011, *696*, 2702-2710; eS.-J. Lu, H.-G. Xu, X.-L. Xu, W.-J. Zheng, *J. Phys. Chem. C* 2017, *121*, 11851-11861; fD.-S. Yang, M. Z. Zgierski, A. Bérces, P. A. Hackett, A. Martinez, D. R. Salahub, *Chem. Phys. Lett.* 1997, *277*, 71-78.

[70] aM. A. Alvarez, D. García-Vivó, M. E. García, M. E. Martínez, A. Ramos, M. A. Ruiz, *Organometallics* 2008, *27*, 1973-1975; bM. A. Alvarez, M. E. García, D. García-Vivó, S. Menéndez, M. A. Ruiz, *Organometallics* 2012, *32*, 218-231; cR. Ponec, G. Yuzhakov, R. Carbó-Dorca, *J. Comput. Chem.* 2003, *24*, 1829-1838; dS. C. Liu, W. L. Ke, J. S. K. Yu, T. S. Kuo, Y. C. Tsai, *Angew. Chem.* 2012, *124*, 6500-6503; eI. Y. Chang, J. S. K. Yu, *Chem. Eur. J.* 2012, *18*, 9189-9193; fY. Chen, S. Sakaki, *Dalton Trans.* 2014, *43*, 11478-11492; gH. Orita, K. Uchida, N. Itoh, *J. Mol. Catal. A: Chem.* 2003, *195*, 173-180; hM. Carrasco, N. Curado, C. Maya, R. Peloso, A. Rodríguez, E. Ruiz, S. Alvarez, E. Carmona, *Angew. Chem. Int. Ed.* 2013, *52*, 3227-3231; iM.-H. Baik, R. A. Friesner, G. Parkin, *Polyhedron* 2004, *23*, 2879-2900; jY. Zhao, Y. Xue, W. Xu, J. H. Su, B. Wu, X. J. Yang, *Eur. J. Inorg. Chem.* 2016, *2016*, 5411-5417; kA. R. Mountain, N. Kaltsoyannis, *Dalton Trans.* 2013, *42*, 13477-13486; lN. Takagi, A. Krapp, G. Frenking, *Inorg. Chem.* 2011, *50*, 819-826; mW. Zou, Y. Liu, W. Liu, T. Wang, J. E. Boggs, *J. Phys. Chem. A* 2009, *114*, 646-651; nY. Zhao, X. Feng, Y. Xie, R. B. King, H. Schaefer III, *J. Phys. Chem. A* 2012, *116*, 5698-5706; oM. C. Neary, G. Parkin, *Inorg. Chem.* 2017, *56*, 1511-1523; pB. Mondal, M. Bhattacharyya, B. Varghese, S. Ghosh, *Dalton Trans.* 2016, *45*, 10999-11007; qP. Petrović, J. P. Djukic, A. Hansen, C. Bannwarth, S. Grimme, A. Maharromov, K. Mahmudov, M. Kopylovich, *Non-covalent stabilization in transition metal coordination and organometallic complexes*, Wiley-VCH: Weinheim, Germany, 2016; rK. K. Chakrahari, R. Ramalakshmi, D. Sharmila, S. Ghosh, *J. Chem. Sci.* 2014, *126*, 1597-1603; sX. Xu, Q. Peng, M.-X. Xie, *Chin. J. Struct. Chem.* 2012, *31*, 1287-1294; tM. R. Haque, S. Ghosh, G. Hogarth, M. G. Richmond, S. E. Kabir, *Inorg. Chim. Acta* 2015, *434*, 150-157; uY. Nishimoto, PhD thesis, 2015; vL. Jonasson, PhD thesis, UCL (University College London) 2009; wJ. Andrés, V. S. Safont, L. Gracia, R. Llusar, E. Longo, in *Recent Advances in Complex Functional Materials: From Design to Application* (Eds.: E. Longo, F. d. A. La Porta), Springer International Publishing, Cham, 2017, pp. 427-454; xS. K. Bose, K. Geetharani, S. Sahoo, K. H. K. Reddy, B. Varghese, E. D. Jemmis, S. Ghosh, *Inorg. Chem.* 2011, *50*, 9414-9422.

[71] aL. J. Farrugia, H. M. Senn, *J. Phys. Chem. A* 2011, *116*, 738-746; bM. Niskanen, P. Hirva, M. Haukka, *J. Mol. Model.* 2012, *18*, 1961-1968; cM. Niskanen, P. Hirva, M. Haukka, *J. Chem. Theory Comput.* 2009, *5*, 1084-1090; dM. Niskanen, P. Hirva, M. Haukka, *PCCP* 2010, *12*, 9777-9782; eE. P. Fowe, B. Therrien, G. Süss-Fink, C. Daul, *Inorg. Chem.* 2008, *47*, 42-48; fM. Shieh, M. H. Hsu, W. S. Sheu, L. F. Jang, S. F. Lin, Y. Y. Chu, C. Y. Miu, Y. W. Lai, H. L. Liu, J. L. Her, *Chem. Eur. J.* 2007, *13*, 6605-6616; gO. V. Sizova, A. Y. Sokolov, L. V. Skripnikov, V. I. Baranovski, *Polyhedron* 2007, *26*, 4680-4690.

[72] M. A. Alvarez, M. E. García, D. Garcia-Vivo, S. Melon, M. A. Ruiz, A. Toyos, *Inorg. Chem.* 2014, *53*, 4739-4750.

[73] aF. Poineau, L. Gagliardi, P. M. Forster, A. P. Sattelberger, K. R. Czerwinski, *Dalton Trans.* 2009, 5954-5959; bL. Gagliardi, B. O. Roos, *Inorg. Chem.* 2003, *42*, 1599-1603; cR. Ponec, L. s. Bučinský, C. Gatti, *J. Chem. Theory Comput.* 2010, *6*, 3113-3121; dX. Gao, N. Li, R. B. King, H. F. Schaefer, *J. Mol. Model.* 2015, *21*, 7; eR. D. Adams, Y. O. Wong, Q. Zhang, *Organometallics* 2013, *32*, 7540-7546; fY. O. Wong, 2014; gM. Costas, J. M. Poblet, M.-M. Rohmer, M. Benard, *Inorg. Chem.* 1995, *34*, 176-183; hB. Xu, Q.-S. Li, Y. Xie, R. B. King, *J. Mol. Model.* 2016, *22*, 157.

[74] aS. Khan, S. Pal, N. Kathewad, I. Purushothaman, S. De, P. Parameswaran, *Chem. Commun.* 2016, *52*, 3880-3882; bD. I. Bezuidenhout, B. van der Westhuizen, A. J. Rosenthal, M. Wörle, D. C. Liles, I. Fernández, *Dalton Trans.* 2014, *43*, 398-401; cX.-R. You, W.-J. Tian, D.-Z. Li, Y.-J. Wang, R. Li, L.-Y. Feng, H.-J. Zhai, *PCCP* 2016, *18*, 13423-13431; dD. Bhattacharjee, B. K. Mishra, A. K. Chakrabartty, R. C. Deka, *Comput. Theor. Chem.* 2014, *1034*, 61-72; eL. Belpassi, F. Tarantelli, A. Sgamellotti, H. M. Quiney, *J. Phys. Chem. A* 2006, *110*, 4543-4554; fX. Li, D. Zhang, *Eur. Phys. J. D* 2013, *67*; gD. Bhattacharjee, B. K. Mishra, R. C. Deka, *J. Mater. Sci. - Mater. Electron.* 2015, *50*, 4586-4599; hK. L. Vikse, A. Zavras, T. H. Thomas, A. Ariafard, G. N. Khairallah, A. J. Canty, B. F. Yates, R. A. O'Hair, *Organometallics* 2015, *34*, 3255-3263; iD.-Z. Li, S.-D. Li, *J. Cluster Sci.* 2011, *22*, 331-341.

[75] aD.-M. Su, X.-J. Zheng, G. Schreckenbach, Q.-J. Pan, *Organometallics* 2015, *34*, 5225-5232; bB. O. Roos, L. Gagliardi, *Inorg. Chem.* 2006, *45*, 803-807; cG. La Macchia, M. Brynda, L. Gagliardi, *Angew. Chem. Int. Ed.* 2006, *45*, 6210-6213.

[76] T. Yoshimura, K. Umakoshi, Y. Sasaki, A. G. Sykes, *Inorg. Chem.* 1999, *38*, 5557-5564.

[77] N. Naumov, E. Ostanina, A. Virovets, M. Schmidtman, A. Müller, V. Fedorov, *Russ. Chem. Bull.* 2002, *51*, 866-871.

[78] W. Bronger, T. Schuster, *Z. Anorg. Allg. Chem.* 1990, *587*, 74-79.

[79] J. R. Long, L. S. McCarty, R. Holm, *J. Am. Chem. Soc.* 1996, *118*, 4603-4616.

[80] M. W. Willer, J. R. Long, C. C. McLauchlan, R. Holm, *Inorg. Chem.* 1998, *37*, 328-333.

[81] L. G. Beauvais, M. P. Shores, J. R. Long, *Chem. Mater.* 1998, *10*, 3783-3786.

[82] S. A. Baudron, A. Deluzet, K. Boubekeur, P. Batail, *Chem. Commun.* 2002, 2124-2125.

[83] T. Yoshimura, C. Suo, K. Tsuge, S. Ishizaka, K. Nozaki, Y. Sasaki, N. Kitamura, A. Shinohara, *Inorg. Chem.* 2010, *49*, 531-540.
[84] W. Bronger, C. Koppe, D. Schmitz, *Z. Anorg. Allg. Chem.* 1997, *623*, 239-242.
[85] Z. Zheng, J. R. Long, R. Holm, *J. Am. Chem. Soc.* 1997, *119*, 2163-2171.
[86] T. G. Gray, R. Holm, *Inorg. Chem.* 2002, *41*, 4211-4216.
[87] P. J. Orto, G. S. Nichol, N. Okumura, D. H. Evans, R. Arratia-Pérez, R. Ramirez-Tagle, R. Wang, Z. Zheng, *Dalton Trans.* 2008, 4247-4253.
[88] F. Klaiber, W. Petter, F. Hulliger, *J. Solid State Chem.* 1983, *46*, 112-120.
[89] Y. Kim, S.-M. Park, W. Nam, S.-J. Kim, *Chem. Commun.* 2001, 1470-1471.
[90] M. Pyziak, J. Pyziak, M. Hoffmann, M. Kubicki, *Crystal Growth & Design* 2015, *15*, 5223-5232.
[91] M. Pizzonero, L. Keller, F. Dumas, M. Ourevitch, G. Morgant, A. Spasojević-de Biré, G. Bogdanović, N. E. Ghermani, J. d'Angelo, *J. Org. Chem.* 2004, *69*, 4336-4350.
[92] N. Lugan, I. Fernandez, R. Brousses, D. A. Valyaev, G. Lavigne, N. A. Ustynyuk, *Dalton Trans.* 2013, *42*, 898-901.
[93] A. C. Tsipis, C. E. Kefalidis, C. A. Tsipis, *J. Am. Chem. Soc.* 2008, *130*, 9144-9155.
